# Juvenile Gadoid Distributions Are Driven by Patch Boundaries and Habitat Combinations

**DOI:** 10.1002/ece3.73032

**Published:** 2026-02-16

**Authors:** Graeme Cullen, David M. Bailey, Charlotte R. Hopkins, Neil M. Burns

**Affiliations:** ^1^ School of Biodiversity, One Health and Veterinary Medicine University of Glasgow Glasgow UK; ^2^ Department of Rural Economy, Environment and Society SRUC Edinburgh UK; ^3^ School of Environmental and Life Sciences, Faculty of Science and Engineering University of Hull Hull UK; ^4^ School of Social & Environmental Sustainability University of Glasgow Dumfries UK

**Keywords:** Atlantic cod ( *Gadus morhua* ), haddock ( *Melanogrammus aeglefinus* ), joint species distribution model (JSDM), seascape, whiting ( *Merlangius merlangus* )

## Abstract

Fish nursery areas need to be determined at the appropriate spatial scale with an understanding of how juvenile fish are distributed across combinations and arrangements of habitat types within seascapes. A seascape approach allows the influence of seabed type, patch sizes, boundaries, and habitat combinations on species distributions to be understood. This study investigated the influence of seascape ecology and species co‐occurrence patterns on the distribution of three juvenile gadoids, Atlantic cod ( *Gadus morhua* ), haddock ( *Melanogrammus aeglefinus* ), and whiting ( *Merlangius merlangus* ), while also accounting for the interactions between species as latent variables. We used 757 stereo baited remote underwater video (SBRUV) deployments between 2021 and 2023 across two sea lochs and adjacent bays on the north and west coasts of Scotland to gather relative abundance data. A joint species distribution model was used to determine the seascape drivers of 10 fish species as well as using an unobserved random variable to understand how species co‐occurrences influence individual species distributions. Atlantic cod, haddock, and whiting distributions were driven by distinct ecological factors yet had limited areas of overlap. Atlantic cod and haddock were most abundant in areas with a diverse mix of habitats. However, whiting were most abundant in areas with lower habitat diversity. Consistently higher relative abundance of all species near habitat patch boundaries indicates that edge effects and access to multiple habitat types are critical determinants of nursery habitat. Despite their distinct distributions explained by environmental variables, species‐to‐species co‐occurrence patterns were very similar across the three species. This may be indicative of similar ecological roles and biological interactions or similar response to an unrecorded variable. The inclusion of how species respond to seascape structures is valuable because it gives a greater level of understanding of what juvenile fish need in nursery areas at the relevant spatial scale.

## Graphical abstract

This study used 757 stereo baited remote underwater video deployments across two Scottish sea lochs and adjacent bays (2021–2023) to investigate how seascape structure and species co‐occurrence shape the distributions of juvenile Atlantic cod ( *Gadus morhua* ), haddock ( *Melanogrammus aeglefinus* ), and whiting ( *Merlangius merlangus* ). A joint species distribution model revealed that cod and haddock were most abundant in areas with diverse habitats, whiting in less diverse areas, and that all three species were consistently associated with habitat patch boundaries. These findings highlight the importance of edge effects, habitat diversity, and species interactions in defining nursery areas at ecologically relevant spatial scales.

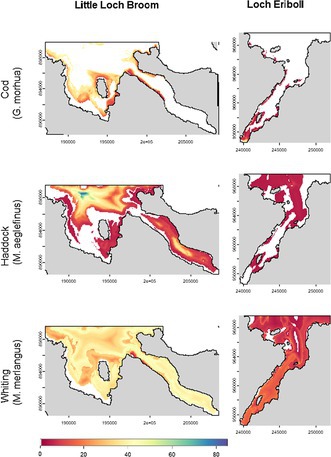

## 1 Introduction

Temperate fish species are threatened by over‐exploitation and declining habitat availability and quality (Fernandez‐Arcaya et al. 2024; Wernberg et al. 2016). The juvenile, young, and growing fish of many temperate species are dependent on nursery areas (Elliott, Sabatino, et al. 2017; Ikpewe et al. 2021), and the availability and carrying capacity of these nursery areas can act as a limiting factor influencing the success of individuals recruiting to adult populations (Able 1999; Beck et al. 2001; Gillanders et al. 2003; Nagelkerken 2009; Nagelkerken et al. 2000). Understanding of essential fish habitat for young, growing fish of most species is limited (Cooke et al. 2022; Pessanha et al. 2021) (James et al. 2019; Ooi and Chong 2011; Sheaves et al. 2015). To ensure essential fish habitats are determined at the appropriate scale, investigations must apply ecological concepts and incorporate spatial resolutions that can best capture the ecological processes of fish (Cooke et al. 2022; Pessanha et al. 2021; Pittman et al. 2011, 2021).

Seascape ecology seeks to understand how the configuration and structure of a mosaic of habitats affects marine species at defined spatial and temporal scales relevant to the species being investigated (Pittman et al. 2021). An example of this is inshore demersal seascapes in temperate regions, which consist of a mosaic of habitats defined by their substratum type (such as rock, sand, or mud) with varying characteristics (such as water turbidity and topography), which are ecologically and hydrologically connected (Boswarva et al. 2018; Burns et al. 2024; Micallef et al. 2012; Sheaves et al. 2015). Seascape ecology can help identify important habitat combinations and give an improved understanding of individual species' ecology at scales relevant to the processes being investigated (Borland et al. 2021; Lacharité and Brown 2019; Pittman et al. 2011). For example, in tropical environments, there is understanding of how fish assemblages are affected by the scale of structural complexity and connectivity in coral, macroalgal, and mangrove habitats within seascapes (Berkström et al. 2020; Moustaka, Evans, et al. 2024). Understanding how fish use habitats to meet needs, such as foraging and refuge, requires examining how species distributions respond to environmental drivers and seascape composition. This depends on determining the influence of seascape features such as patch boundaries and habitat diversity on species distributions at the appropriate scale (Luo et al. 2009; Moustaka, Evans, et al. 2024; Vaslet et al. 2015).

Additionally, species co‐occurrence patterns are valuable to include in an analysis, and understanding of species distributions as environmental influences alone are not sufficient to explain distribution patterns given species often interact with others on a daily basis (Astarloa et al. 2019; da Silva et al. 2023; Ovaskainen, Abrego, et al. 2016; Pollock et al. 2014). Interacting species can have a positive or negative influence on a species' distribution, for example, through facilitation or competition respectively (Tikhonov et al. 2020). Ecological interactions between species play a crucial role in determining a species' distribution and by not incorporating them into analysis the full picture may be missed (Kraan et al. 2020; Pollock et al. 2014). For example, in an experimental setting, juvenile roach ( *Rutilus rutilus* ) exhibited differing habitat use and behaviours when exposed to predator cues, indicating that the presence of predators can influence their distribution and habitat selection (Martin et al. 2010). Therefore, a better understanding of fish nursery areas can be gained by incorporating seascape ecology and species co‐occurrences into analysis.

Atlantic cod ( *Gadus morhua* ), haddock ( *Melanogrammus aeglefinus* ) and whiting ( *Merlangius merlangus* ) are three commercially important gadoid species which have had low stocks since the early 1980s on the West coast of Scotland (ICES division 6.a). For example, in 1982, the spawning stock biomass of Atlantic cod was estimated at 45,000 t, but has declined to approximately 5000 t since 2002, which is substantially lower than the 20,000 t maximum sustainable yield reference point (Alexander et al. 2014; Ellis et al. 2024; Fernandes and Cook 2013; ICES 2013, 2022a, 2022b, 2024a). Haddock stocks in Division 6.a have shown episodic recruitment variability, with strong year classes (e.g., 2006 and 2019) providing only temporary increases in biomass. Haddock spawning stock biomass has generally remained below the maximum sustainable yield, as measured by biomass, in recent years, despite reductions in fishing mortality (ICES 2024b). Whiting stocks have remained persistently low, with ICES assessments classifying the stock as having reduced reproductive capacity and subject to high fishing pressure, prompting recommendations for zero or minimal catch in several recent years (ICES 2024c).

Success in the juvenile life stage is crucial for recruitment to adult populations and therefore the recovery of gadoid stocks (Caddy 2014; Laurel et al. 2016; Lough 2010). Juvenile survival is heavily influenced by the quality of the habitats they occupy. The quality of nursery areas is dependent on the availability of species‐specific shelter, foraging opportunities, environmental conditions (such as temperature and salinity), currents, and interactions with other species, all of which are facilitated by favourable seascape structure (Dunne et al. 2023; Moustaka, Robbins, et al. 2024; Nagelkerken et al. 2000).

Gadoids are a useful group of study species for seascape ecology as they are similar in size and functional group, and are all demersal; however, they differ in behaviour and habitat preferences (Elliott, Turrell, et al. 2017). These behaviours and habitat preferences make them ideal candidates for examining how seascape arrangements and features or interspecific interactions may affect fish distributions. Yet, it is still not fully understood how species‐to‐species co‐occurrences between juvenile gadoids and other fish influence gadoid distributions at the seascape scale (Elliott, Turrell, et al. 2017). Filling this gap is important, as the presence or absence of competitors, predators, or facilitators could determine how nursery areas support recruitment. Improved understanding of seascape drivers and species co‐occurrences during the juvenile stage of gadoids could therefore help identify the key limiting factors affecting their early survival and the recovery of their populations.

The present study aimed to (a) understand the spatial distribution of three juvenile gadoid species (Atlantic cod ( *G. morhua* ), haddock ( *M. aeglefinus* ), and whiting ( *M. merlangus* )) within seascapes and (b) explain the differences in spatial distribution between the three study focal species. Stereo baited remote underwater video (SBRUV) was used to gather fish relative abundance data in two sea lochs and adjacent bays on the North and West coasts of Scotland. This data was used to build a joint species distribution model (JSDM) that included seascape metrics.

## 2 Methods

### 2.1 Study Area and Sampling Regime

Data were collected at two fjordic sea lochs and adjacent bays on the north and west coasts of Scotland. Loch Eriboll and Sango Bay, hereafter referred to as Loch Eriboll, have a surface area of 63 km^2^. Little Loch Broom and Grunyard Bay, hereafter referred to as Little Loch Broom, have a surface area of 98 km^2^. Data collection was carried out from 2021 to 2023, during summer to coincide with the post‐settlement of juvenile gadoids (Table 1).

**TABLE 1:** Stereo Baited Remote Underwater Video (SBRUV) deployments by study site, year and field season timings. 780 SBRUV deployments total.

Site	Year	Start date	End date	Number of SBRUV deployments
Eriboll	2021	04/08/2021	24/08/2021	216
Eriboll	2022	27/07/2022	08/08/2022	116
Little Loch Broom	2022	22/08/2022	07/09/2022	216
Little Loch Broom	2023	17/07/2023	26/07/2023	116
Eriboll	2023	07/08/2023	14/08/2023	116

**FIGURE 1:**
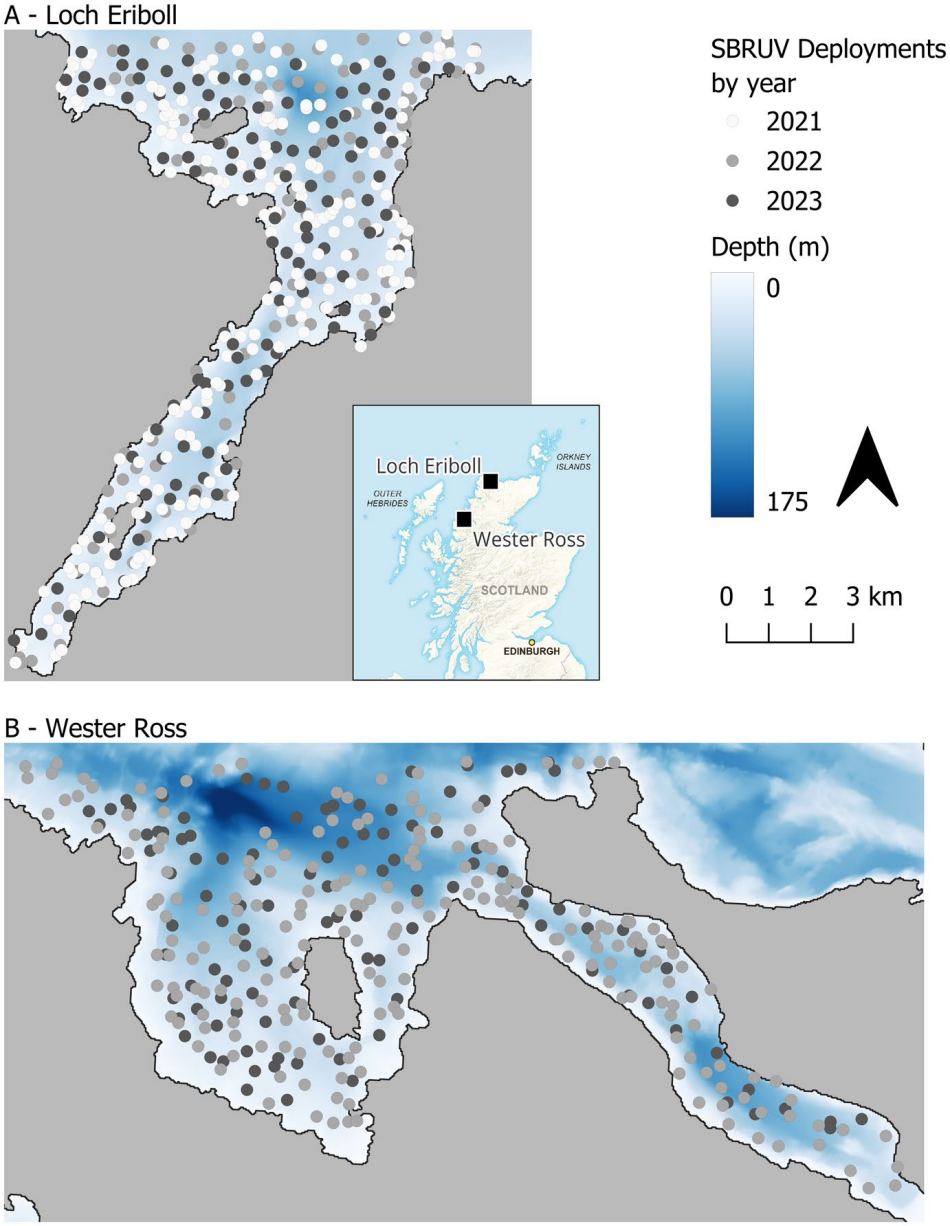
Study sites and SBRUV deployment locations by year. Panel A shows Loch Eriboll and Panel B shows Little Loch Broom. Dots show the location of each SBRUV deployment, with the colour of the dot representing the year of the deployment. Bathymetric data are also presented. Panels A and B are at the same spatial scale. Inset: black squares show the study site locations in Scotland, United Kingdom. Contains OS data Crown copyright and database right 2020. Figure created using QGIS version 3.28 (QGIS.org 2024).

Fish relative abundance data were collected by demersal SBRUV (equipment specifications below). A depth‐stratified sampling design was implemented to ensure comparable sampling effort across depth bands. Six depth strata were defined as 0 to 9.9 m, 10 to 18.9 m, 19 to 29.9 m, 30 to 37.9 m, 38 to 49.9 m and 50 to 116 m (Figure 1), each representing 10.4 km^2^ of seabed within the study areas. We standardised effort by randomly assigning an equal number of SBRUV deployments to each depth stratum. SBRUV units were deployed on the seabed and sampled a fixed‐point location. This ensured equal sampling effort per depth stratum rather than estimating a spatial footprint of observation. This design ensured balanced representation of depth zones while avoiding assumptions about the effective sampling area of individual SBRUV deployments.

### 2.2 Species Relative Abundance Data

Three SBRUV systems were used, one per deployment, each consisting of two GoPro Hero 9 cameras in waterproof housings on a metal frame, with two Scubapro Nova 850 (wide) dive torches. For each SBRUV deployment, a perforated bait box was filled with approximately 100 g of mackerel ( *Scomber scombrus* ). The video footage was analysed using EventMeasure software (SeaGIS 2022), which was used to record MaxN (the highest number of individuals seen in one frame for each mobile animal species) (Langlois et al. 2020; SeaGIS 2022; Watson et al. 2010). The EventMeasure software (SeaGIS 2022) also allows measurements to be taken. Measurements were taken at or as close to MaxN as possible to prevent double measurement of fish. Fish were measured, and all gadoids less than 200 mm in length were counted as juvenile (Elliott, Turrell, et al. 2017; Walker‐Milne et al. 2025). Of the 780 SBRUV deployments, 757 SBRUV deployments were included in the analysis, with 23 SBRUV deployments removed because visibility was impaired by debris covering > 50% of the field of view or a lack of light.

To make model fitting computationally feasible and avoid making vague inferences about infrequently encountered species, any species which were not fish and occurred in fewer than 10% of the SBRUV deployments at each site each year (e.g., present in fewer than 16 drops, out of a total 116, for Loch Eriboll in 2022) was removed from modelling (74 species, 88% of species were removed). A species accumulation curve (Appendix A1.3) was created with the R package Vegan (Oksanen et al. 2025) to assess sampling adequacy. The curve's gradient represents the rate of new species discovery per sample.

### 2.3 Environmental Data

The bathymetry data was derived from the General Bathymetric Chart of the Oceans (British Crown and OceanWise 2018). Slope gradient and slope aspect were derived from the bathymetry data. The immediate substratum type at each SBRUV deployment was determined by two observers. In cases where there were multiple seabed types visible, the most dominant seabed type was recorded (Burns et al. 2024). Seascape metrics (see below) were derived from seabed maps for the Loch Eriboll (Burns et al. 2024) and Little Loch Broom study sites.

All analyses used the software R version 4.2.3 (R Core Team 2022) with spatial data processing and analysis using the sf (Pebesma and Bivand 2023) and terra (Hijmans 2023) R packages. To understand the effect of the wider seascape on fish distribution, Shannon diversity was calculated for the number and proportion of surrounding substratum types. The Shannon diversity metric was chosen over other diversity metrics to allow the relative proportions (evenness) of the different substratum types as well as the number of different substratum (diversity) types to be incorporated in the analysis (Ngcobo et al. 2025). The Shannon index of substratum diversity (hereafter referred to as surrounding patch diversity) was tested in model selection at 500, 1000 and 1500 m radius circle sizes around each SBRUV deployment. The radii were chosen based on the plausible range of juvenile gadoid movement per day, using the calculations of Elliott, Sabatino, et al. (2017). Elliott, Sabatino, et al. (2017) measured the cruising speed of juvenile gadoids (less than 20 cm long) using SBRUV data, in addition to data from other studies, to approximate the possible distance a juvenile gadoid can move in a day (Cote et al. 2004; Grant and Brown 1998; Laurel et al. 2004; Løkkeborg et al. 2002; Tobin et al. 2010; Wright et al. 2006). This gave a maximum movement radius for age 0 gadoids of 1500 m (Elliott, Sabatino, et al. 2017). When a surrounding patch diversity circle comprised land or areas outside of the study area (where the substratum map was not available), only the area of the circle within the study area was included in the Shannon diversity index calculation.

**FIGURE 2:**
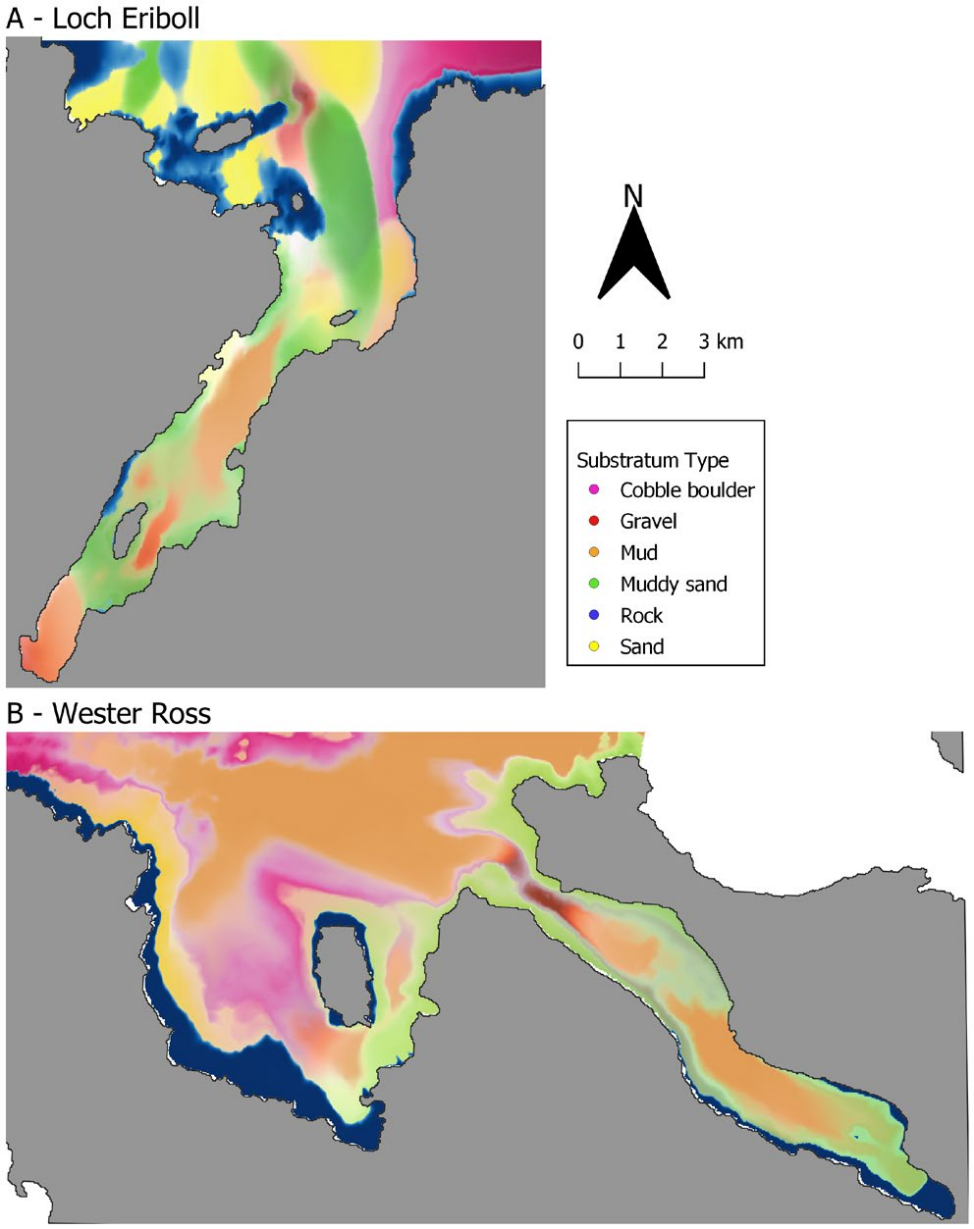
Substratum maps for the two study sites: Loch Eriboll (A) and Little Loch Broom (B). Map created in QGIS version 3.34 (QGIS.org 2024). Substratum types are shown with different probability cutoffs. Higher probability cutoffs (0.25) were used for substratum types with hard edges (rock and cobble boulder substratum types). A lower probability cutoff (0.1) was used for all other substratum types (mud, muddy sand, sand and gravel substratum types) to allow for their ‘fuzzy’ boundaries. Little Loch Broom (in prep) and Loch Eriboll data sourced from Burns et al. (2024).

To understand if and how fish distributions are affected by patch boundaries, the distance to the nearest habitat patch boundary was measured for each SBRUV deployment. The substratum maps provided by Burns et al. (2024) (see Figure 2) provide separate raster layers for each substratum type with the values of the probability of each substratum type's presence. As different substratum types have probabilities above zero in overlapping areas, patch boundaries were defined by removing low probabilities from each substratum raster. This differed by substratum type to allow for ‘fuzzy’ boundaries (Burns et al. 2024). Higher probability cutoffs (0.25) were used for substratum types with hard edges (rock and cobble boulder substratum types). Lower probability cutoffs (0.1) were used for all other substratum types (mud, muddy sand, sand and gravel substratum types) to allow for their ‘fuzzy’ boundaries. Additionally, distance to shoreline was tested in model selection to understand if the physical boundary impacts gadoid distribution. The shoreline was not used as a patch boundary. The coordinates of each SBRUV deployment were also included in model selection to test for spatial auto correlation. In The hierarchical modelling of species communities (Hmsc) R package, spatial autocorrelation is accounted for by including the spatial coordinates of each sampling location as a random effect with a spatially structured covariance matrix. Coordinates are supplied as spatial random levels, which Hmsc models using a Gaussian structure that assumes sites closer together are more similar than those farther apart (Tikhonov et al. 2020). See Table A3.1 in Data S1 for a summary of environmental variables.

### 2.4 Species Distribution Modelling

The Hmsc R package, version 3.0–13, was used for the joint species distribution modelling (JSDM) (Tikhonov et al. 2020). Markov chain Monte Carlo (MCMC) models were run with a Poisson distribution specified for the response variable (MaxN—species relative abundance).

JSDMs allow for the modelling of the abundances of multiple species so that species co‐occurrences can be estimated using the residual variation once environmental variables have been accounted for. The Hmsc framework was used to include an unobserved (or latent) variable to utilise residual variation to model how species co‐occurrences, or an unrecorded variable, affect the spatial distribution of the species included in the analysis (Ovaskainen, Abrego, et al. 2016; Ovaskainen, Roy, et al. 2016; Ovaskainen and Abrego 2020). Species co‐occurrences are modelled in Hmsc through the inclusion of a species covariance matrix which increases confidence that results can be interpreted as species co‐occurrence associations or shared response to an unrecorded environmental variable (Ovaskainen, Abrego, et al. 2016; Ovaskainen and Abrego 2020; Tikhonov et al. 2020). However, species co‐occurrence associations modelled from a latent variable cannot determine ecological relationships between species as they are not being modelled directly (Blanchet et al. 2020).

The default Hmsc flat priors were used. Fixed effects (environmental covariates) were assigned multivariate normal distribution priors with a mean of zero and a variance of 10^4^. Variance parameters for the random effects (site, year, and species co‐occurrences) were assigned non‐informative Gamma distribution priors with shape and rate parameters both set to 0.1 (Ovaskainen, Roy, et al. 2016; Ovaskainen and Abrego 2020; Tikhonov et al. 2020). Models were run with 8 chains for 30,000 iterations each, out of which 10,000 were transient (burn‐in). The remaining samples were evenly thinned by 10 samples to produce 2000 samples for each chain (16,000 per model).

Model convergence was assessed for the fixed effects (*β*) and random effect variance components parameter groups in the HMSC model. Convergence was evaluated using both visual inspection of MCMC trace plots and quantitative diagnostics (see Appendix 4 in Data S1). Trace plots were examined to ensure good mixing and the absence of trends across chains, while the potential scale‐reduction factor (R̂) was required to be close to 1 (typically < 1.1) and the effective sample size large enough to indicate adequate independent sampling (see Appendix 4 in Data S1). These combined criteria were used to confirm that all monitored parameters had converged (Ovaskainen and Abrego 2020).

Widely Applicable Information Criterion (WAIC) was used in forward backward stepwise model selection. Final model performance was assessed using 5‐fold cross‐validation using root‐mean‐square error (RMSE) as the metric of model predictive power (Tikhonov et al. 2020; Wilkinson et al. 2021; Zhang et al. 2021).

Variables tested in model selection were substratum type, depth, slope, aspect, distance to shore, distance to patch boundary, and surrounding patch diversity (Shannon index tested at 500, 1000, and 1500 m radius circles).

Distance to shore was found to correlate with depth through variance inflation factor testing, producing a value of 8.423. Depth was chosen to be included in the model instead of distance to shore as depth was found to have a greater influence on model performance, as measured by WAIC, during model selection.

### 2.5 The Final Model

The model formula for the final model was as follows:

The response variable relative abundance (MaxN):

$$y_{\mathrm{ij}}\sim \text{Poisson}\left(\lambda_{\mathrm{ij}}\right)$$

with a log link function:

$$\begin{array}{c} \log\left(\lambda_{\mathrm{ij}}\right)=a_{j}+\beta_{1j}\text{Substratu}m_{i}+\beta_{2j}\text{Dept}h_{i}+\beta_{3j}\text{Dist}.\;\text{to nearest patch boundar}y_{i}+\beta_{4j}\text{Surrounding patch diversit}y_{i}+\\ \;\beta_{5j}\left(\text{Dept}h_{i}\times \text{Substratum}\;\mathrm{typ}e_{i}\right)+\beta_{6j}\left(\text{Substratum}\;\mathrm{typ}e_{i}\times \text{Dist}.\;\text{nearest patch boundar}y_{i}\right)+\\ \beta_{7j}\left(\text{Dept}h_{i}\times \text{surrounding patch diversit}y_{i}\right)+\varepsilon_{\mathrm{ij}}^{S}+\varepsilon_{\mathrm{ij}}^{Y}+u_{i}v_{j} \end{array}$$

where *y* is the MaxN for species *j*, *α* and *β* are the intercept and slope parameters for covariates. The ε parameters are the random effects at the site (*S*) and year (*Y*) levels to account for differences between the study sites and years. *U* is the value of the latent variable at each deployment (*i*) and *V* is the strength and direction of the association of each species (*j*) with the latent variable (Gomo et al. 2020; Tikhonov et al. 2020).

## 3 Results

### 3.1 Species Assemblage Characterisation

In the analysed SBRUV footage species diversity ranged from 0 to 13 (mean 5.1 ± 2.3 SD, see Figure A1.1 in Data S1). Mean mobile animal species diversity at Loch Eriboll and Little Loch Broom study sites was similar as measured by Shannon diversity index for all species recorded (Loch Eriboll mean 1.123 ± 0.548 SD; Little Loch Broom mean = 1.144 ± 0.431 SD).

The 10 species included in the analysis were: whiting ( *Merlangius merlangus* ), poor cod (*Trispoteris minutus*), haddock (*Melanorammus aeglefinus*), plaice ( *Pleuronectes platessa* ), flounder ( *Platichthys flesus* ), Atlantic cod ( *Gadus morhua* ), pollock ( *Pollachius pollachius* ), lesser spotted dogfish ( *Scyliorhinus canicula* ), grey gurnard ( *Eutrigla gurnardus* ) and painted goby ( *Pomatoschistus pictus* ) (see Table A2.1 in Data S1 for full species list).

Sampling adequacy, as measured by the species accumulation curve, gave a gradient representing the rate of new species discovered per sample. After 101 samples, the gradient was < 0.1, indicating sufficient effort to approximate community diversity and support ecological inference (see Figure A1.3 in Data S1). The final gradient was 0.018 new species per sample (Thompson et al. 2003; Thompson and Withers 2003).

### 3.2 Model Selection

Surrounding patch diversity at a 500 m radius circle decreased model WAIC, the most and was therefore included in the model over 1000 and 1500 m radius circle sizes. Slope and aspect were also removed during model selection as they did not improve model WAIC. The coordinates for each deployment were not kept in the model during model selection and therefore spatial autocorrelation was not deemed to be an issue that needed to be accounted for (see Table A3.2 in Data S1) (Ovaskainen and Abrego 2020; Tikhonov et al. 2020; Zuur et al. 2009).

### 3.3 Model Performance

The potential scale reduction factor (R̂) was centred around 1.01 indicating the chains gave consistent results and the model converged (Ovaskainen and Abrego 2020; Tikhonov et al. 2020). The effective sample size was 3677.805, which is lower than the maximum posterior samples drawn (16,000). Posterior distributions for all parameters were examined to ensure that sampling adequately explored parameter space (see Appendix 5 in Data S1). Within the Hmsc framework, a relatively low effective sample size is accepted when using non‐normally distributed data. As the potential scale reduction factor was close to 1.01, and all posterior estimates were stable with no divergent transitions, the ESS values were considered sufficient to support valid inference (see Figure A4.1 in Data S1) (Tikhonov et al. 2020).

The final model's predictive power, assessed using 5‐fold cross‐validation, resulted in Root mean square error (RMSE) values of 1.875 for haddock ( *M. aeglefinus* ) (haddock MaxN ranged from 0 to 25), 1.633 for Atlantic cod ( *G. morhua* ) (cod MaxN varied from 0 to 24), and 6.596 for whiting ( *M. merlangus* ) (whiting MaxN ranged from 0 to 48). These RMSE values correspond to 7.5%, 6.8%, and 13.7% of the respective response ranges, indicating moderate to good predictive accuracy relative to the scale of the data (Janssen and Heuberger 1995). Results for all species are reported in Table 2.

**TABLE 2:** Root mean square error (RMSE) from 5‐fold cross validation for all species included in the analysis. Response range is the range of MaxN values for each species.

Species	Response range	RMSE
Atlantic cod ( *G. morhua* )	0–24	1.633
Haddock ( *M. aeglefinus* )	0–25	1.910
Whiting ( *M. merlangus* )	0–48	9.587
Poor cod ( *T. minutus* )	0–123	9.811
Plaice ( *P. platessa* )	0–30	2.313
Flounder ( *P. flesus* )	0–22	2.157
Pollock ( *P. pollachius* )	0–89	16.162
Lesser spotted dogfish ( *S. canicula* )	0–4	0.659
Grey gurnard ( *E. gurnardus* )	0–2	0.692
Painted goby ( *P. pictus* )	0–4	0.703

### 3.4 Seascape Drivers of Gadoid Distribution

The inclusion of surrounding patch diversity, dependent on depth, and distance to the nearest patch boundary, dependent on substratum type, in the final model indicates that the combination and arrangement of habitats (seascape ecology) influenced the distribution of juvenile gadoids (see Figure A1.2 in Data S1).

For all species included in the model, the interaction between depth and substratum type (35.9% of variance explained), substratum type (24.9% of variance explained), and the interaction between distance to nearest patch boundary and substratum type (21.1% of variance explained) had the greatest influence on the distribution of all species (See Figure A3.1 in Data S1). The random effects of site and year accounted for a mean of 2% and 3.9% of the variance respectively for all species.

#### 3.4.1 Atlantic Cod ( *Gadus morhua* )

Atlantic cod ( *G. morhua* ) were predicted by the model to be most abundant on rock and gravel substratum types. Atlantic cod were predicted to be more abundant further from patch boundaries when on rock substratum type but more abundant closer to patch boundaries when on gravel. Atlantic cod were also predicted to be more abundant in areas with a mix of different substratum patches. The interaction between depth and substratum had the greatest influence in predicting Atlantic cod abundances, accounting for 74% of the variance explained by the model.

#### 3.4.2 Haddock ( *Melanogrammus aeglefinus* )

Haddock ( *M. aeglefinus* ) were predicted by the model to be most abundant on deeper patches of sand, further from patch boundaries in areas with a diverse mix of different habitat types. Additionally, haddock were predicted to be abundant on gravel and muddy sand substratum types. Haddock abundance tended to decrease with distance to patch boundary on gravel and cobble boulder habitats, while it remains relatively constant or even increases slightly on mud and sandy substrates. The interaction between distance to patch boundary and substratum type was the most important variable in explaining haddock relative abundance, accounting for 35% of the explained variance. Additionally, substratum type explained 18% of the explained variance; the interaction between depth and substratum type explained 17% of the explained variance; and distance to patch boundary explained 14% of the explained variance.

#### 3.4.3 Whiting ( *Merlangius merlangus* )

Whiting ( *M. merlangus* ) was predicted by the model to occur at relatively high abundance on most substratum types in shallower areas, apart from cobble boulder. However, in deeper areas, whiting were predicted only to be highly abundant on rock. Whiting were also predicted to be abundant closer to patch boundaries, with the exception of mud substratum type, in areas with fewer habitat types. Substratum type was the most important variable in explaining whiting relative abundance, accounting for 56% of the explained variance. The interaction between depth and substratum explained 16% of the explained variance, and the random effect of site explained 12% of the explained variance.

#### 3.4.4 Overall Species Environmental Trends

**FIGURE 3:**
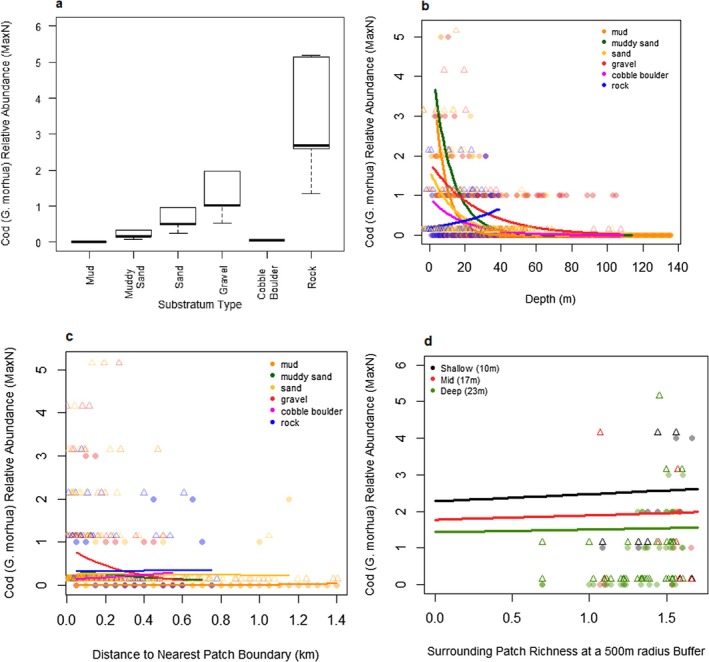
Atlantic Cod ( *G. morhua* )—Predicted Atlantic cod responses (relative abundance—MaxN) to fixed environmental effects. Predictions were made with non‐plotted variables set to the observed mean and limited to observed range (see Table A3.3 in Data S1) and random effects were set to site = Loch Eriboll and year = 2022. Circles are data predicted by the final model from observed environmental data. Triangles are observed data points jittered by +0.15 to make them visible and comparable to the predicted data. (a) predicted Atlantic cod MaxN for substratum type with all other variables set to observed mean for each substratum type. (b) predicted Atlantic cod MaxN for depth by substratum type, with depths limited to observed ranges and all other variables set to observed mean. (c) predicted Atlantic cod ( *G. morhua* ) MaxN for distance to patch boundary by substratum type with all other variables set to observed mean. (d) predicted Atlantic cod MaxN for surrounding patch diversity (Shannon diversity index at a 500 m radius circle) with varying depths and all other variables set to observed mean and substratum set to gravel.

**FIGURE 4:**
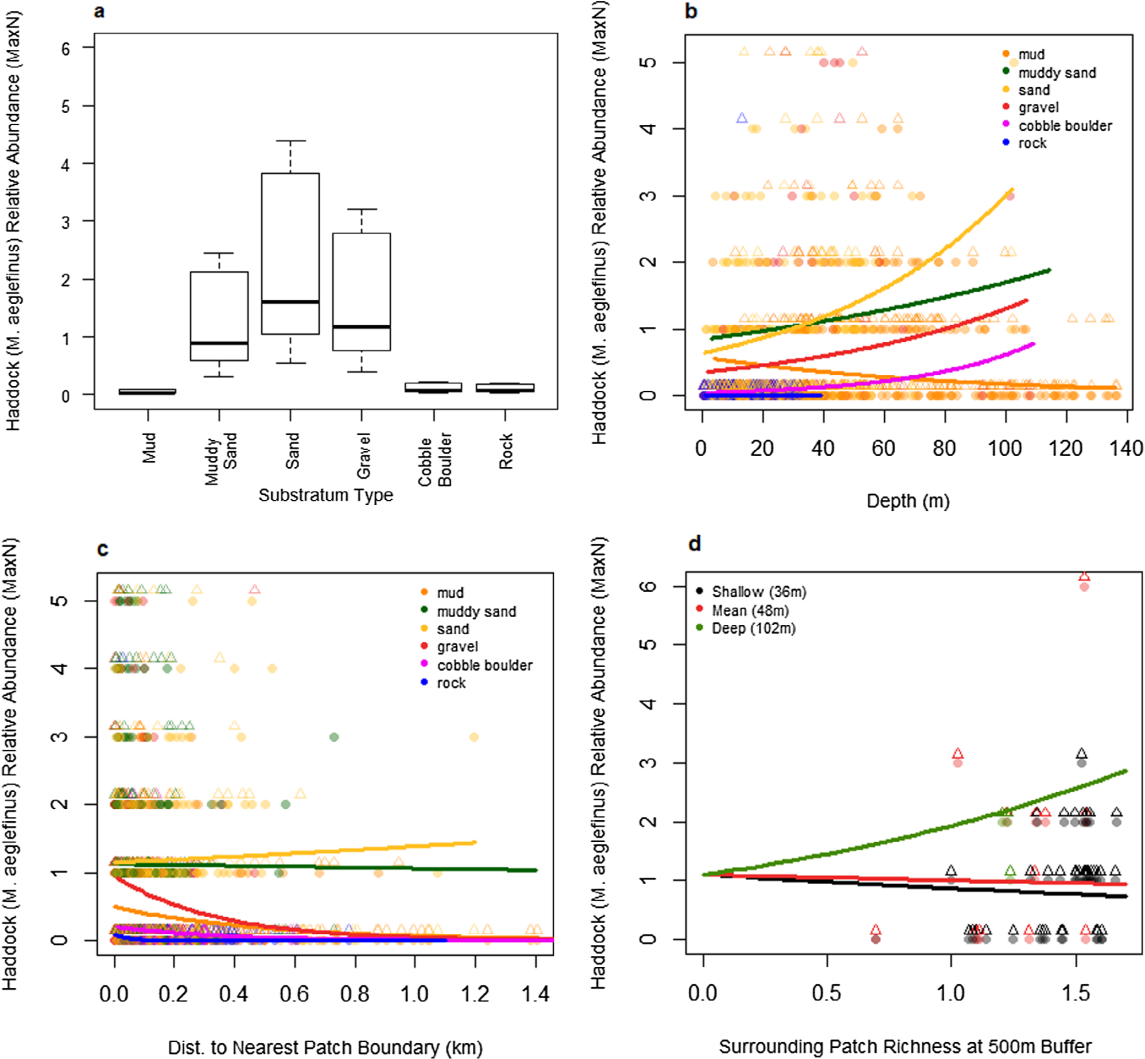
Haddock ( *M. aeglefinus* )—Predicted model haddock ( *M. aeglefinus* ) responses (relative abundance—MaxN) to fixed environmental effects. Predictions were made with non‐plotted variables set to the observed means for haddock ( *M. aeglefinus* ) presences (see Table A3.3 in Data S1) and random effects were set to site = Loch Eriboll and year = 2022. Circles are data predicted by the final model from observed environmental data. Triangles are observed data points jittered by +0.15 to make them visible and comparable to the predicted data. (a) predicted haddock ( *M. aeglefinus* ) MaxN for substratum type. (b) predicted haddock ( *M. aeglefinus* ) MaxN for depth by substratum type. (c) predicted haddock ( *M. aeglefinus* ) MaxN for distance to patch boundary by substratum type. (d) predicted haddock ( *M. aeglefinus* ) MaxN for surrounding patch diversity (Shannon diversity index at a 500 m radius circle) with varying depths and all other variables set to observed mean with substratum set to gravel.

**FIGURE 5:**
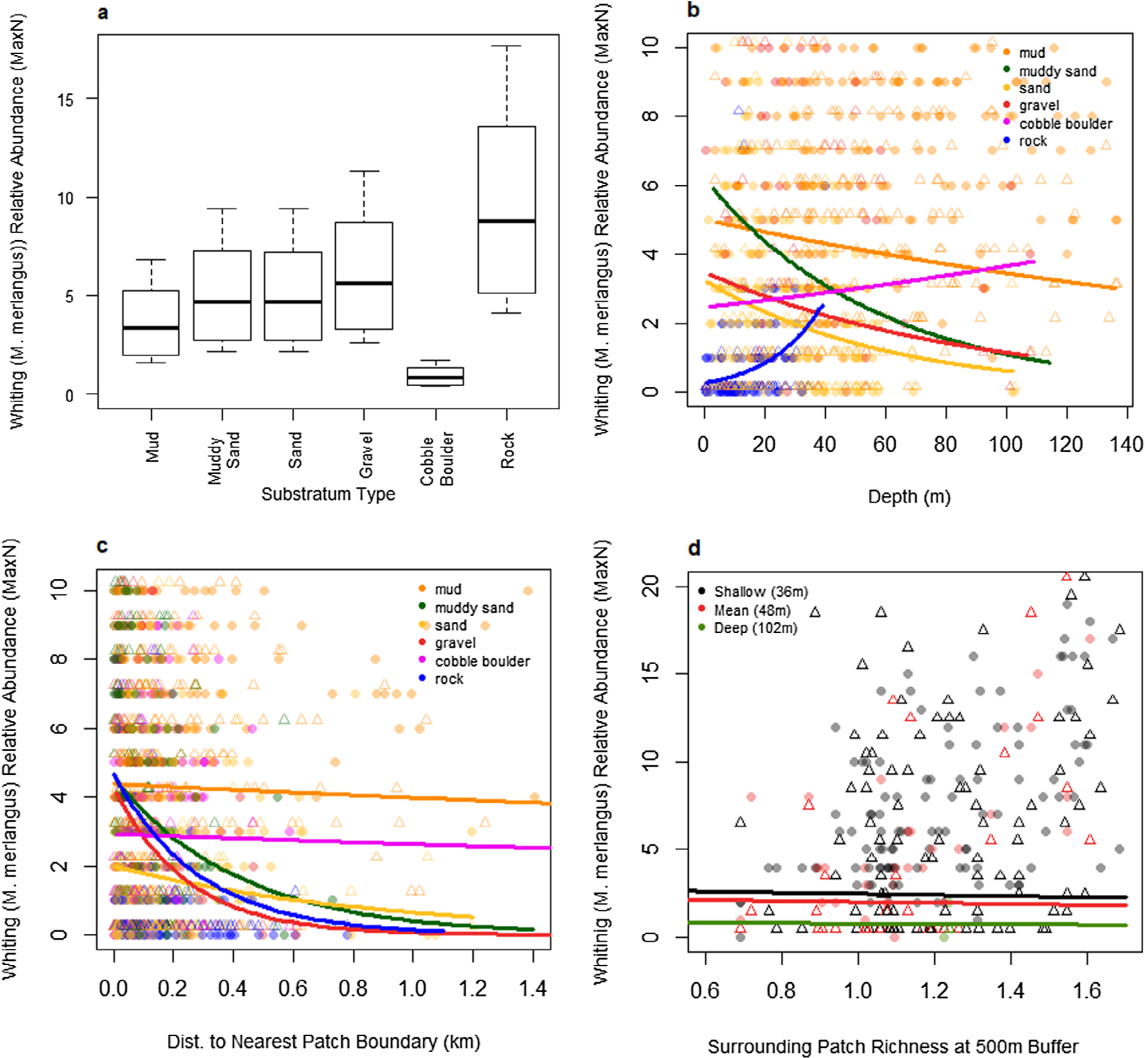
Whiting ( *M. merlangus* )—Predicted model whiting responses (relative abundance—MaxN) to fixed environmental effects. Predictions were made with non‐plotted variables set to the observed mean for whiting presences (see Table A3.3 in Data S1) and random effects were set to site = Loch Eriboll and year = 2022. Circles are data predicted by the final model from observed environmental data. Triangles are observed data points jittered by +0.15 to make them visible and comparable to the predicted data. (a) predicted whiting MaxN for substratum type. (b) predicted whiting MaxN for depth (made positive) by substratum type, with the depth ranges for each substratum type limited to the observed ranges. (c) predicted whiting MaxN for distance to patch boundary by substratum type. (d) predicted whiting MaxN for surrounding patch diversity (Shannon diversity index at a 500 m radius circle) at varying depths with all other variables set to observed mean and substratum set to muddy sand.

All three focal species showed relatively distinct, but in some areas overlapping, distributions within the seascape. Atlantic cod and haddock were most abundant in areas with a diverse mix of different habitat types on a few specific substratum types for each species (see Figures 3 and 4). However, whiting were most abundant in areas with lower habitat diversity but on most substratum types (see Figure 5). Notably, all species showed higher relative abundance near habitat patch boundaries on some substratum types.

The remaining demersal fish species showed varied responses to environmental variables (see Figure A1.4 in Data S1), suggesting species‐specific habitat preferences across the seascape. Pollack and painted goby were both positively associated with patch diversity and negatively associated with depth and most substratum types, indicating a preference for shallower areas with different habitats available. Plaice showed positive associations with gravel and muddy sand and was negatively associated with distance to patch boundaries. Poor cod and flounder displayed strong responses to depth and sediment type, with both positively associated with mud and gravel and negatively with sand. Grey gurnard was negatively associated with many substrata and patch diversity but positively associated with mud and muddy sand near patch boundaries, which may be indicative of use of fuzzy patch boundaries only where mud and muddy sand share a boundary. Lesser spotted dogfish showed more limited associations but responded positively to sand and negatively to rocky substrates. Overall, while species like pollack and painted goby appear to favour patchy, shallow, and diverse areas, others like plaice and poor cod show stronger affinities for specific sediment types. Edge effects were evident in several species, suggesting that habitat boundaries play an important role in structuring fish distributions even beyond the three focal species.

### 3.5 Predicted Distribution Maps

**FIGURE 6:**
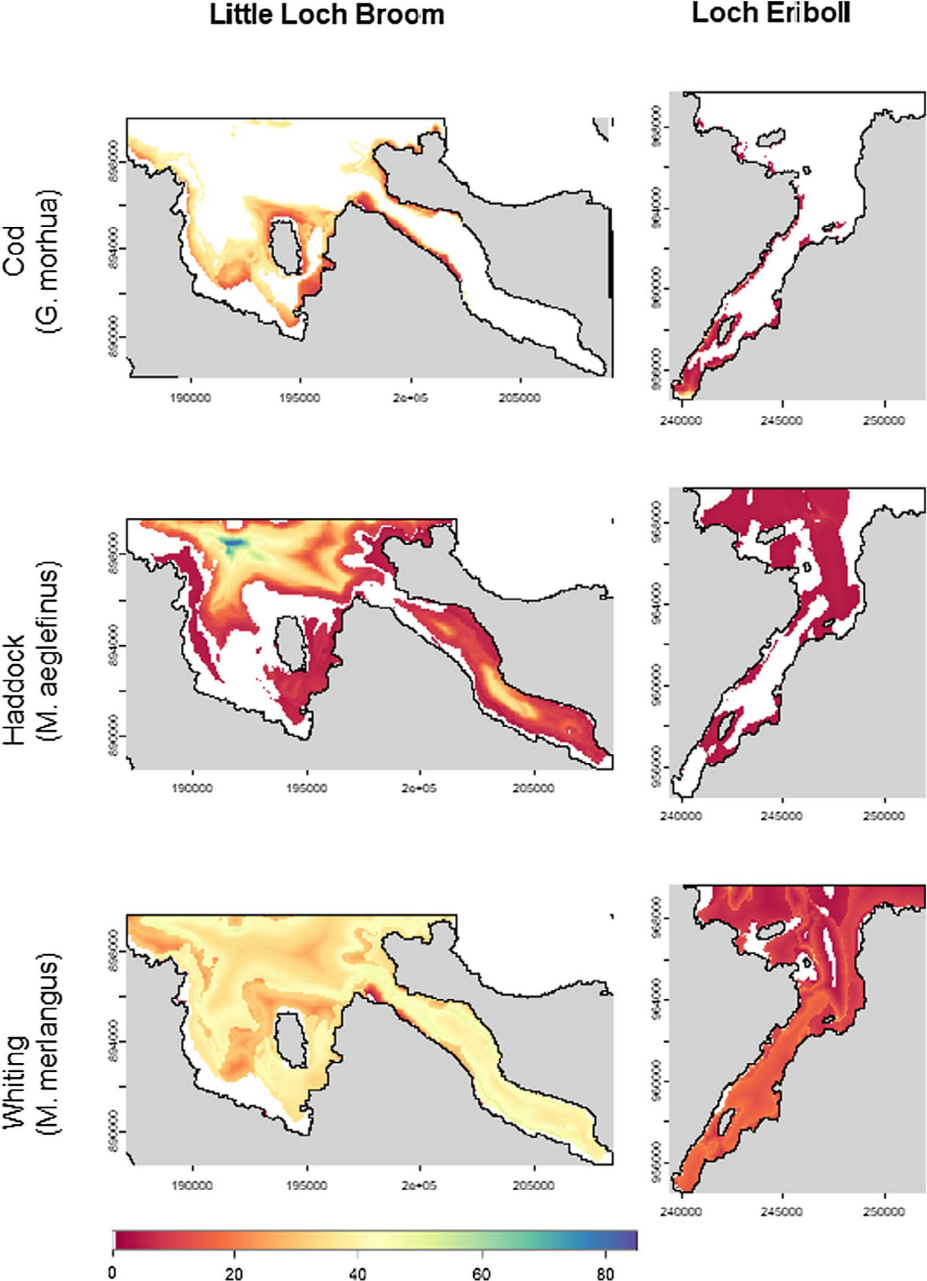
Predicted spatial distribution heat maps for Atlantic cod ( *G. morhua* ), haddock ( *M. aeglefinus* ) and whiting ( *M. merlangus* ) for each study site; left‐hand panels show Loch Eriboll and right‐hand panels show Little Loch Broom. All maps were predicted with the random effect year set to 2022 as this was the year with the most data points (SBRUV deployments).

The preference of the three focal species for patch boundaries and multiple habitat use can be seen in the distribution patterns as relative abundances peak and change sharply along patch boundaries (see Figure 6). The habitat preferences and differences between species are also visible. Atlantic cod ( *G. morhua* ) were predicted to be the most spatially restricted of the focal species, predicted to be present in 26.9% of the Little Loch Broom study site and 10% of the Loch Eriboll study site. Haddock ( *M. aeglefinus* ) are predicted to have a larger extent, predicted to be present in 66.2% of the Little Loch Broom study site and 49.8% of the Loch Eriboll study site. Whiting ( *M. merlangus* ) is predicted to be abundant throughout almost all of the seascapes, being predicted to be present in 88.9% of the Little Loch Broom study site and 89.9% of the Loch Eriboll study site.

### 3.6 Species Co‐Occurrences

**FIGURE 7:**
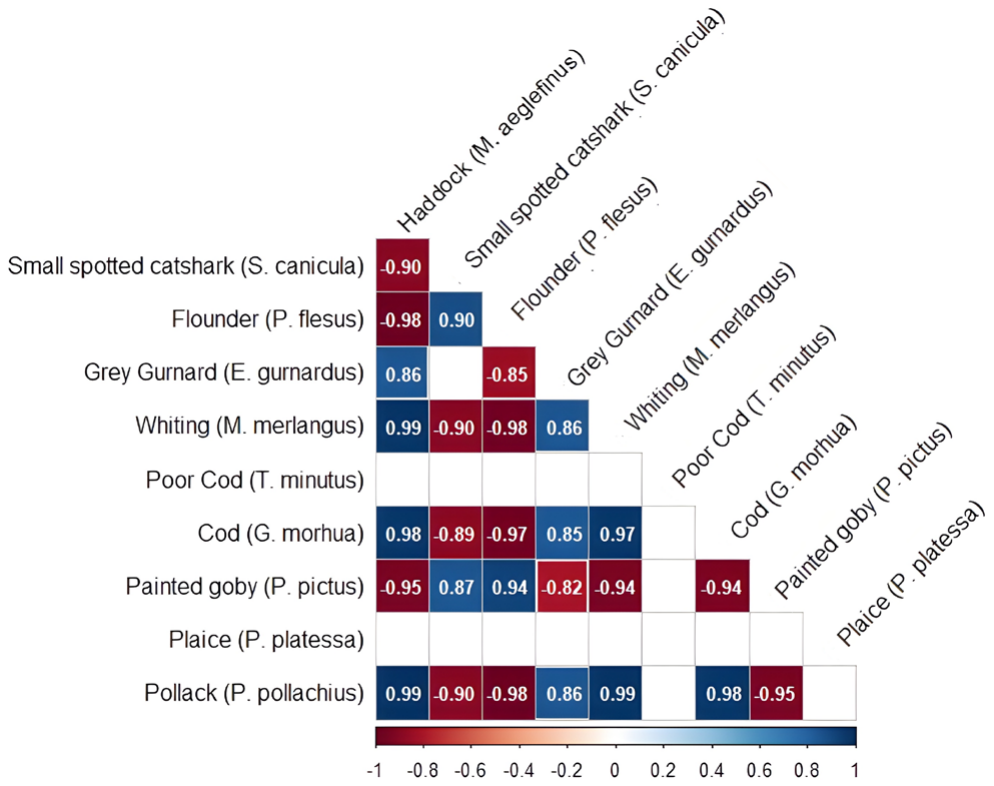
Correlation plot showing species occurrence correlations modelled using an unobserved latent variable. Blank boxes represent no species co‐occurrences at a 95% credible interval. Co‐occurrence associations shown are at the 95% credible interval (posterior credible interval does not overlap zero) with the mean presented in each box and colours representing positive (blue) or negative (red) associations.

We found strong species co‐occurrences that were explained by the unobserved (latent) variable included in the model (see Figure 7). We found that poor cod ( *T. minutus* ) and plaice ( *P. platessa* ) did not show co‐occurrence associations with other species at a 95% credible interval (the 95% credible intervals for their correlation coefficients overlapped zero). All other species showed species co‐occurrence associations at a 95% credible interval apart from between lesser spotted dogfish ( *S. canicula* ) and grey gurnard (*E. gurnadus*). Atlantic cod ( *G. morhua* ), haddock ( *M. aeglefinus* ), and whiting ( *M. merlangus* ) all have a positive co‐occurrence association with each other. This indicates that when one of these species is abundant, the others are also likely to be more abundant than expected based on the fixed environmental variables and random effects alone. Atlantic cod, haddock, and whiting also all have a positive co‐occurrence association with grey gurnard and pollack, and all have a negative co‐occurrence association with lesser spotted dogfish, flounder ( *P. flesus* ), and painted goby ( *P. pictus* ).

## 4 Discussion

The present study modelled the distributions and species co‐occurrence associations of gadoids in two sea lochs and adjacent bays in Northwest Scotland. The differences in distributions between Atlantic cod ( *G. morhua* ), haddock ( *M. aeglefinus* ), and whiting ( *M. merlangus* ) were explained by relationships with seascape and environmental factors. Atlantic cod were most abundant in shallower areas on rock and gravel substrata, with cod favouring areas further from patch edges on rock but closer to edges on gravel, in areas with high surrounding patch diversity. Haddock were predicted to be most abundant on deeper patches of sand, further from patch boundaries in areas with high surrounding patch diversity. Haddock were also predicted to be abundant on gravel and muddy sand substratum types close to patch boundaries. Whiting were predicted to be abundant on all substratum types, generally being more abundant in shallower areas, closer to patch boundaries in areas of lower surrounding patch diversity.

Whiting were abundant on all habitat types which indicates this species does not rely on specific habitats to meet its needs (Henderson 2019). This ecological versatility may enhance resilience against environmental changes and anthropogenic impacts such as habitat degradation or shifting benthic community structures. In contrast to more habitat‐specialist species, generalists like whiting can exploit a wide range of resources and conditions, allowing them to maintain stable populations even if specific habitats are not available (Devictor et al. 2008). This may help explain why whiting, compared to other similar demersal fish such as Atlantic cod, appears to be relatively more resilient under recent pressures, including fishing pressure and climate‐driven habitat shifts (Bastrikin et al. 2014; Engelhard et al. 2014; Henderson 2019; ICES 2022a).

Higher Atlantic cod and haddock relative abundance in more substrata diverse areas may be indicative of multiple habitat use (Bracewell et al. 2018; Kostylev et al. 2005; Smith et al. 2014). Atlantic cod were predicted to be more abundant in areas of higher surrounding patch diversity which aligns with Elliott, Sabatino, et al. (2017), Elliott, Turrell, et al. (2017) who also found increased abundances with surrounding patch diversity. Atlantic cod have been recorded in situ in the Western Atlantic and in experimental conditions, using multiple habitats in order to grow and survive (by avoiding predators) (Laurel et al. 2003; Lunzmann‐Cooke et al. 2021). Our results therefore build on the findings that juvenile gadoids, like other juvenile fish species, need multiple habitats to be able to meet their needs (Cooke et al. 2022; Elliott, Sabatino, et al. 2017; Pessanha et al. 2021), and here we have identified the specific combinations and arrangements of habitats for the study focal species. These specific combinations and arrangements of habitats for Atlantic cod and haddock may be limiting factors affecting the recruitment of individuals to adult populations, where previously abundance limitations due to habitat constraints for these species have been found at larger scales (Asjes et al. 2016; Bastrikin et al. 2014; Blanchard et al. 2005).

The distance to patch boundary was retained during model selection indicating edge effects. Edge effects occur when habitat conditions differ between the centre and edge of a patch. In muddy habitats, turbidity is typically higher in the core, where fine sediments are resuspended by tides, waves, or burrowing fauna, and lower at the edges where coarser sediments dominate (Grabowski et al. 2011). Elevated turbidity reduces light penetration, limiting both primary productivity and visual predation. Animals may select inner patches to avoid predation (Barbier et al. 2011; Besterman and Pace 2021; Colomer and Serra 2021; Colvin and Snelgrove 2025; Matias et al. 2013; Montie and Thomsen 2023). Edge effects are dependent on the two substratum types that share the patch boundary. For example, how gradual the transition is between the two patches (or patch boundary ‘fuzziness’) is dependent on the substratum type, with fine mixed sediments (such as between mud and sand) having more gradual patch boundaries and harder substratum types (such as rock) having clearly defined patch boundaries (Burns et al. 2024; Matias et al. 2013). The present study's results show that the effect of substratum type on relative abundance is modulated by distance to patch boundary. For Atlantic cod, haddock and whiting, there were edge effects between the habitat patches where each species was most abundant. Atlantic cod was most abundant on rock and gravel substratum types, resulting in peak abundances at their boundaries. The edge effects observed in the present study are comparable to those documented in terrestrial systems, such as birds, where ecological boundaries influence predation risk, foraging efficiency, and species distributions (Howell et al. 2021; Vallejos et al. 2024).

Here, we found strong species co‐occurrences explained by an unobserved (latent) variable. Despite relatively distinct distributions, Atlantic cod, haddock, and whiting showed very similar species co‐occurrences with each other and the seven other species included in the analysis. Patterns of species association inferred from Joint Species Distribution Models (JSDMs) using co‐occurrence data cannot determine ecological relationships between species. This is because JSDMs do not explicitly model ecological interactions; they infer residual species associations only after accounting for shared environmental responses. As a result, any apparent co‐occurrence patterns may reflect unmeasured environmental factors or shared responses to the same conditions, rather than direct biotic interactions between species (Blanchet et al. 2020). The associations from residual variation may be indicative of potential species co‐occurrences (Araújo and Rozenfeld 2014; Ovaskainen, Abrego, et al. 2016; Ovaskainen and Abrego 2020). For example, positive relationships observed in co‐occurrence data may be indicative of predation, as predators would be expected to be found close to their prey (Montanyès et al. 2023).

Pollack ( *P. pollachius* ) is known to predate juvenile gadoids (Howson and Picton 1997), therefore, the strong positive co‐occurrence pattern reported in the present study likely reflects pollack associating with areas of high juvenile gadoid abundance, resulting in a positive co‐occurrence pattern driven by predator behaviour rather than prey habitat choice. Similarly, positive associations among Atlantic cod, haddock, and whiting, despite documented conspecific predation (Elliott et al. 2018; Köster et al. 2014), are more plausibly explained by shared habitat preferences or a common response to unmeasured environmental drivers. Importantly, predator–prey interactions can also produce negative correlations in JSDMs when predation pressure is strong enough to create spatial separation between predators and prey (Montanyès et al. 2023), highlighting that a co‐occurrence pattern should not be interpreted mechanically without ecological context. Atlantic cod, haddock, and whiting's negative relationship with lesser spotted dogfish ( *Scyliorhinus canicula* ) may be indicative of competition, as they exploit similar prey resources such as benthic invertebrates and small bony fishes (Köhlmann 1987; Scott 1988). If one of these species was more abundant in a particular area, it could indicate that the species was out‐competing the other species for prey. However, here, the use of bait may influence the likelihood of co‐occurrence, and our results differ from those reported by Montanyès et al. (2023) Variability in habitat heterogeneity and methodological approaches (e.g., baited vs. trawl sampling) can significantly influence observed interspecific associations, particularly when comparing juvenile and adult life stages (Haak et al. 2020; Isaksson et al. 2025; Laegdsgaard and Johnson 2001; Nielsen et al. 2021; Zhang et al. 2023). Further research investigating juvenile gadoid species co‐occurrences directly, in addition to the influence of bait, is needed to clarify the species co‐occurrence results of the present and other studies.

The results of this study add to our understanding of juvenile gadoid nursery habitat by defining seascape scale distributions of juvenile gadoids at a local scale. Whiting nursery habitats have previously been determined on a regional scale for the west coast of Scotland using depth, sediment type and temperature (Burns et al. 2019). Specific, fine scale juvenile gadoid distributions in relation to seascape metrics build on this understanding by identifying juvenile whiting distributions in more specific areas and habitat combinations. Previous studies at a regional scale for haddock could not identify distinguished nursery areas (Asjes et al. 2016). The present study has identified potential areas where juvenile haddock distributions may be limited by environmental factors and the availability of specific combinations and arrangements of habitats. The juvenile gadoid substratum and depth associations found in the present study were similar to the findings of Elliott, Sabatino, et al. (2017) and Elliott, Turrell, et al. (2017) depth associations, but did not report occurrences on rock substrata, as this substratum type was not included in the analysis. Differences in substratum classification and seascape context likely account for variation in fine‐scale patterns, though consistent depth and substratum preferences suggest stable habitat associations across the west coast of Scotland. Therefore the present study has identified distributions in relation to specific seascape structures and areas where juvenile gadoid distributions concentrate. The present study also incorporated species co‐occurrence patterns into our understanding of juvenile gadoid distributions.

## 5 Conclusion

Atlantic cod ( *G. morhua* ), haddock ( *M. aeglefinus* ) and whiting ( *M. merlangus* ) distributions are all influenced by the configuration of the seascape. The present study has shown that including seascape variables enhances species distribution modelling and develops a better understanding of fish habitat requirements than by seabed type alone. Species co‐occurrence between juvenile gadoids and other fish species plays a role in influencing their seascape distribution. The consistent species co‐occurrences for juvenile gadoids with other fish species indicate that changes in other fish species abundances could have an effect on the abundances of Atlantic cod, haddock and whiting. Furthermore, changes in the abundances of either Atlantic cod, haddock or whiting may impact the other gadoid species. These findings have given us greater insight into the nursery areas of juvenile gadoids by identifying potential limiting factors to their survival at a relevant scale.

## Author Contributions

**Graeme Cullen:** data curation (equal), formal analysis (equal), investigation (equal), methodology (equal), software (equal), writing – original draft (lead), writing – review and editing (equal). **David M. Bailey:** conceptualization (equal), funding acquisition (equal), project administration (equal), supervision (equal), writing – review and editing (equal). **Charlotte R. Hopkins:** funding acquisition (equal), supervision (equal), writing – review and editing (equal). **Neil M. Burns:** conceptualization (equal), data curation (equal), funding acquisition (equal), project administration (equal), supervision (equal), writing – review and editing (equal).

## Funding

Graeme Cullen was supported by a PhD scholarship from Scotland's Rural College (SRUC). This study was funded in 2021 by Wildland Ltd. Through the Sustainable Inshore Fisheries Trust (SIFT) this work was further supported by the Scottish Government's Rural & Environment Science & Analytical Services Division (RESAS), through their Strategic Research Programmes (2022–2027) to collect data from 2022. Bathymetric data is used courtesy of the Maritime and Coastguard Agency (MCA) and UK Hydrographic Office, collected as part of the INIS Hydro Interreg IVa Cross border programme managed by the Special EU Programmes Body.

## Conflicts of Interest

The authors declare no conflicts of interest.

## Supporting information


**Data S1:** Supporting Information.

## Data Availability

All code and data used to produce the current manuscript will be available at the author's Git hub for publication and will have a DOI (https://doi.org/10.5281/zenodo.18503486) associated for publication through Fig Share after review. All the required data are uploaded as Supporting Information.
