# Supplementary material for: Juvenile Gadoid Distributions Are Driven by Patch Boundaries and Habitat Combinations
Source: Ecol Evol. 2026 Feb 16;16(2):e73032. doi: 10.1002/ece3.73032 (PMC12909611; doi:10.1002/ece3.73032)
Supplement: Supplementary file 1 — Data S1: Supporting Information. [file ECE3-16-e73032-s001.pdf]

Appendices

Appendix 1 – Summary Statistics Figures

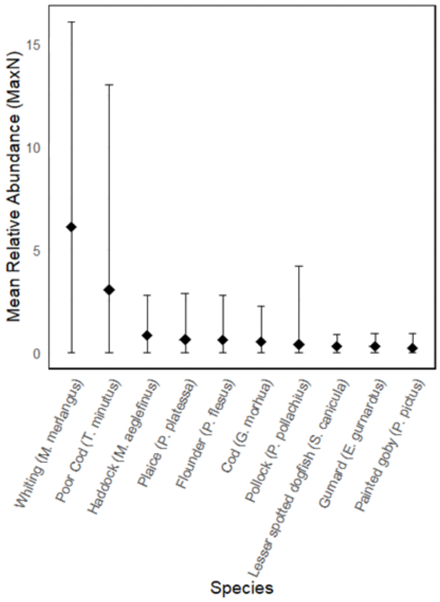

Figure A1.1 – Mean Relative abundance (MaxN) for the 10 species included in analysis. Whiting (*M. Merlangus*) had the highest mean (6.113 ± 9.960 s.d) and painted goby (*P. pictus*) had the lowest mean (0.195 ± 0.702 s.d).

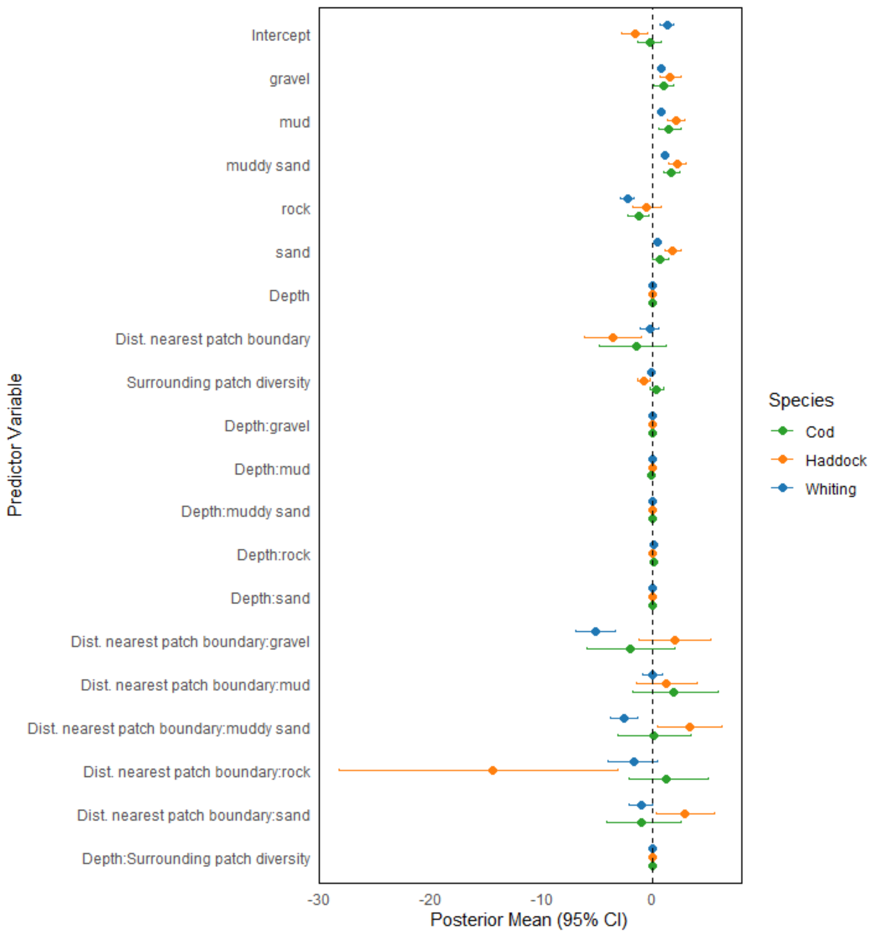

Figure A1.2 – Posterior mean estimates (points) and 95% credible intervals (horizontal lines) for the effects of habitat and environmental predictors on the occurrence of Atlantic cod (*Gadus morhua*), Haddock (*Melanogrammus aeglefinus*), and Whiting (*Merlangius merlangus*). Predictor variables include substrate type, depth, distance to nearest patch boundary, surrounding patch diversity, and their interactions. Estimates are shown separately for each species (green: Cod; orange: Haddock; blue: Whiting). The dashed vertical line at zero indicates no effect.

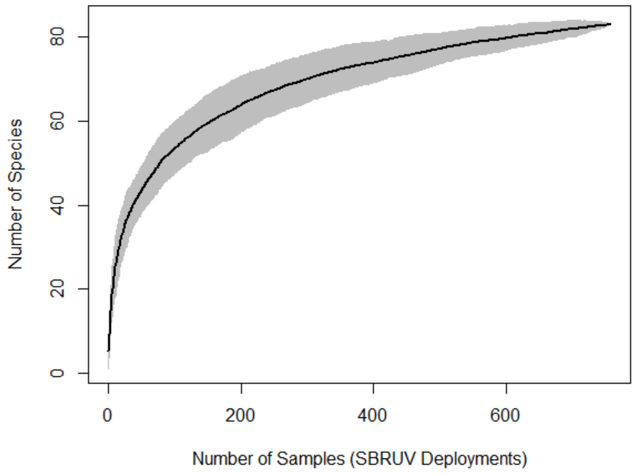

Figure A1.3 – Species accumulation curve We obtained a gradient value below 0.1 after 101 samples (values < 0.1 indicate sampling effort sufficiently captures the community to approximate species diversity and allow for confident ecological inference) and a final gradient value of 0.018 new species per additional sample (Thompson et al., 2003; Thompson & Withers, 2003). The species accumulation curve was created using the R package ‘Vegan’ (Oksanen et al., 2025).

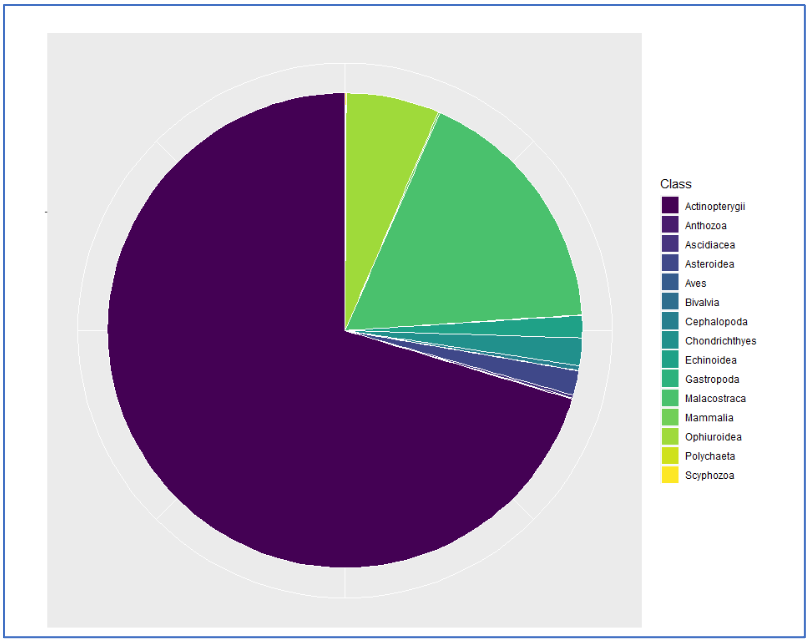

Figure A1.4 – Pie chart of species counts (summed MaxN) grouped by class.

Appendix 2 – Species List

Table A2.1 – Species recorded in the SBRUV data and whether or not they were included (5th column) in the joint species distribution models (JSDMs).

Family	Genus	Species	Included
Squalidae	*Squalus*	*acanthias*	n
Aphroditidae	*Aphrodita*	*aculeata*	n
Gadidae	*Melanogrammus*	*aeglefinus*	**Y**
Stichaeidae	*Chirolophis*	*ascanii*	n
Rajidae	*Dipturus*	*batis*	n
Labridae	*Labrus*	*bergylta*	n
Paguridae	*Pagurus*	*bernhardus*	n
Cottidae	*Taurulus*	*bubalis*	n
Scyliorhinidae	*Scyliorhinus*	*canicula*	**Y**
Semaeostomeae	*Cyanea*	*capillata*	n
Phalacrocoracidae	*Phalacrocorax*	*carbo*	n
Corystidae	*Corystes*	*cassivelaunus*	n
Luidiidae	*Luidia*	*ciliaris*	n
Eledonidae	*Eledone*	*cirrhosa*	n
Rajidae	*Raja*	*clavata*	n
Congridae	*Conger*	*conger*	n
Portunidae	*Liocarcinus*	*depurator*	n
Echinidae	*Echinus*	*esculentus*	n
Gadidae	*Trispoterus*	*esmarkii*	n
Labridae	*Centrolabrus*	*exoletus*	n
Gobiidae	*Gobiusculus*	*flavescens*	n
Pleuronectiformes	*Platichthys*	*flesus*	**Y**
Ophiothricidae	*Ophiothrix*	*fragilis*	n
Nephropidae	*Homarus*	*gammarus*	n
Blenniidae	*Parablennius*	*gattorugine*	n
Asterinidae	*Asterina*	*gibbosa*	n
Asteriidae	*Marthasterias*	*glacialis*	n
Phocidae	*Halichoerus*	*grypus*	n
Triglidae	*Eutrigla*	*gurnardus*	**Y**
Ophiuridae	*Clupea*	*harengus*	n
Rajidae	*Dipturu*	*intermedius*	n
Astropectinidae	*Astropecten*	*irregularis*	n
Pleuronectidae	*microstomus*	*kitt*	n
Cyaneidae	*Cyanea*	*lamarckii*	n
Ammodytidae	*Hyperoplus*	*lanceolatus*	n
Clavelinidae	*Clavelina*	*lepadiformis*	n
Pleuronectidae	*Limanda*	*limanda*	n
Gadidae	*trisopterus*	*luscus*	n
Callionmidae	*Callionymus*	*lyra*	n
Portunidae	*Carcinus*	*maenas*	n

Rajidae	*Raja*	*mantagui*	n
Labridae	*Symphodus*	*melops*	n
Gadidae	*Merlangius*	*merlangus*	**Y**
Gadidae	*Trispoterus*	*minutus*	**Y**
Virgulariidae	*Virgularia*	*mirabilis*	n
Phyllophoridae	*Neopentadctyla*	*mixta*	n
Labridae	*Labrus*	*mixtus*	n
Gadidae	*Molva*	*molva*	n
Gadidae	*Gadus*	*morhua*	**Y**
Rajidae	*Leucoraja*	*naevus*	n
Lamnidae	*Lamna*	*nasus*	n
Ophiotomidae	*Ophiocomina*	*nigra*	n
Nephropidae	*Nephrops*	*norvegicus*	n
Gobiidae	*Pomatoschistus*	*norvegicus*	n
Pectinidae	*Aequipecten*	*opercularis*	n
Ophiuridae	*Ophiura*	*ophiura*	n
Cancridae	*Cancer*	*pagurus*	n
Solasteridae	*Crossaster*	*papposus*	n
Inachidae	*Inachus*	*phalangium*	n
Phocoenidae	*Phocoena*	*phocoena*	n
Pennatulacea	*Pennatula*	*phosphorea*	n
Gobiidae	*Pomatoschistus*	*pictus*	**Y**
Pleuronectidae	*Pleuronectes*	*platessa*	**Y**
Gadidae	*Pollachius*	*pollachius*	**Y**
Portunidae	*Necora*	*puber*	n
Goneplacidae	*Genoplax*	*rhomboides*	n
Inachidae	*Macropodia*	*rostrata*	n
Asteriidae	*Asterias*	*rubens*	n
Galatheidae	*Munida*	*rugosa*	n
Labridae	*Ctenolabrus*	*rupestris*	n
Scombridae	*Scomber*	*scombrus*	n
Cottidae	*Myoxocephalus*	*scorpius*	n
Palaemonidae	*Palaemon*	*serratus*	n
Gasterosteidae	*Spinachia*	*spinachia*	n
Majidae	*Maja*	*squinado*	n
Scyliorhinidae	*Scyliorhinus*	*stellaris*	n
Loliginidae	*Alloteuthis*	*subulata*	n
Carangidae	*Trachurus*	*trachurus*	n
Buccinidae	*Buccinum*	*undatum*	n
Trachinidae	*Echiichthys*	*vipera*	n
Gadidae	*Pollachius*	*virens*	n
Phocidae	*Phoca*	*vitulina*	n
Scorpaenidae	*Sebestes*	*viviparus*	n
Octopodidae	*Octopus*	*vulgaris*	n

33

Appendix 3 – Environmental Data and model selection

Table A3.1 – Summary of tested environmental predictors. * Indicates random effect. Blue rows are those used in the final model.

Predictor	Description	Unit	Range	Source
Sample*	Sample level random effect.	1 – number of samples		
Year*		2021 to 2023 (as a factor)		
Site*		Eriboll (1) or Wester Ross (2) (as a factor)		
Coordinates	Spatial autocorrelation mitigation	EPSG:27700		
Substratum		Mud (1), muddy sand (2), Sand (3), Gravel (4), Cobble Boulder (5), Rock (6)		(N. Burns et al., 2020)
Depth		meters		
Slope		Degrees	0 - 20	
Aspect	Split into Northness and Eastness	Degrees		
Seascape Diversity	Shannon Diversity for Substratum.		0 – 1.5	
Distance to Patch Boundary		Km		

Table A3.2 – Table showing widely applicable information criterion (WAIC) for forward backward stepwise model selection. Random effects of site and year included in all models.

Model	Model Formula	Description	WAIC
0	~ Substratum<br>+ Depth<br>+ Dist. nearest patch boundary<br>+ Surrounding patch diversity<br>+ Coordinates	Full model – 1500 m radius	25.55

	+ Slope<br>+ Aspect<br>+ Substratum:Depth<br>+ Substratum:Dist. nearest patch boundary<br>+ Depth:Surrounding patch diversity<br>+ Dist. nearest patch boundary:Surrounding patch diversity<br>+ Depth:Dist. nearest patch boundary<br>+ Slope:Substratum<br>+ Slope:Depth<br>+ Slope:Dist. nearest patch boundary<br>+ Slope:Surrounding patch diversity<br>+ Slope:Aspect<br>+ Aspect:Substratum<br>+ Aspect:Depth<br>+ Aspect:Dist. nearest patch boundary<br>+ Aspect:Surrounding patch diversity		
0<br>Spatial	~ Substratum<br>+ Depth<br>+ Dist. nearest patch boundary<br>+ Surrounding patch diversity<br>+ Slope<br>+ Aspect<br>+ Substratum:Depth<br>+ Substratum:Dist. nearest patch boundary<br>+ Depth:Surrounding patch diversity<br>+ Dist. nearest patch boundary:Surrounding patch diversity<br>+ Depth:Dist. nearest patch boundary<br>+ Slope:Substratum<br>+ Slope:Depth<br>+ Slope:Dist. nearest patch boundary<br>+ Slope:Surrounding patch diversity<br>+ Slope:Aspect<br>+ Aspect:Substratum<br>+ Aspect:Depth<br>+ Aspect:Dist. nearest patch boundary<br>+ Aspect:Surrounding patch diversity	Removed coordinates	25.27
0.1	~ Substratum<br>+ Depth<br>+ Dist. nearest patch boundary<br>+ Surrounding patch diversity<br>+ Aspect<br>+ Substratum:Depth	Removed slope	26.92

	+ Substratum:Dist. nearest patch boundary<br>+ Depth:Surrounding patch diversity<br>+ Dist. nearest patch boundary:Surrounding patch diversity<br>+ Depth:Dist. nearest patch boundary<br>+ Aspect:Substratum<br>+ Aspect:Depth<br>+ Aspect:Dist. nearest patch boundary<br>+ Aspect:Surrounding patch diversity		
0.11	~ Substratum<br>+ Depth<br>+ Dist. nearest patch boundary<br>+ Surrounding patch diversity<br>+ Substratum:Depth<br>+ Substratum:Dist. nearest patch boundary<br>+ Depth:Surrounding patch diversity<br>+ Dist. nearest patch boundary:Surrounding patch diversity<br>+ Depth:Dist. nearest patch boundary	Removed Aspect	27.03
0.12	~ Substratum<br>+ Depth<br>+ Dist. nearest patch boundary<br>+ Substratum:Depth<br>+ Substratum:Dist. nearest patch boundary + Depth:Dist. nearest patch boundary	Removed surrounding patch diversity 1500 m radius	28.81
0.13	~ Substratum<br>+ Depth<br>+ Dist. nearest patch boundary<br>+ Surrounding patch diversity<br>+ Substratum:Depth<br>+ Substratum:Dist. nearest patch boundary + Depth:Surrounding patch diversity<br>+ Dist. nearest patch boundary:Surrounding patch diversity<br>+ Depth:Dist. nearest patch boundary	1000 m radius	26.30
0.14	~ Substratum<br>+ Depth<br>+ Dist. nearest patch boundary<br>+ Substratum:Depth<br>+ Substratum:Dist. nearest patch boundary + Depth:Dist. nearest patch boundary	Removed 1000 m radius	28.81

0.15	~ Substratum<br>+ Depth<br>+ Dist. nearest patch boundary<br>+ Surrounding patch diversity<br>+ Substratum:Depth<br>+ Substratum:Dist. nearest patch boundary + Depth:Surrounding patch diversity<br>+ Dist. nearest patch boundary:Surrounding patch diversity<br>+ Depth:Dist. nearest patch boundary	500 m radius	22.60
0.16	~ Substratum<br>+ Depth<br>+ Dist. nearest patch boundary<br>+ Substratum:Depth<br>+ Substratum:Dist. nearest patch boundary + Depth:Dist. nearest patch boundary	Removed 500 m radius	28.81
0.2	~ Substratum<br>+ Depth<br>+ Dist. nearest patch boundary<br>+ Surrounding patch diversity<br>+ Substratum:Depth<br>+ Substratum:Dist. nearest patch boundary<br>+ Depth:Surrounding patch diversity<br>+ Dist. nearest patch boundary:Surrounding patch diversity	Removed Depth:Dist. nearest patch boundary	23.54
0.3	~ Substratum<br>+ Depth<br>+ Dist. nearest patch boundary<br>+ Surrounding patch diversity<br>+ Substratum:Depth<br>+ Substratum:Dist. nearest patch boundary<br>+ Depth:Surrounding patch diversity	Removed Dist. nearest patch boundary:Surrounding patch diversity	22.60
0.4	~ Substratum<br>+ Depth<br>+ Dist. nearest patch boundary<br>+ Surrounding patch diversity<br>+ Substratum:Depth<br>+ Substratum:Dist. nearest patch boundary	Removed Depth:Surrounding patch diversity	24.68
0.5	~ Substratum<br>+ Depth<br>+ Dist. nearest patch boundary<br>+ Surrounding patch diversity	Removed Substratum:Dist. nearest patch boundary	24.72

	+ Substratum:Depth		
0.6	~ Substratum<br>+ Depth<br>+ Dist. nearest patch boundary<br>+ Surrounding patch diversity	Removed interaction terms	24.80
0.7	~ Substratum<br>+ Depth<br>+ Surrounding patch diversity	Removed Dist. nearest patch boundary	24.96
0.8	~ Substratum<br>+ Depth	Removed Surrounding patch diversity	23.12
0.9	~ Depth<br>+ Surrounding patch diversity	Removed Substratum	33.25
1.0	~ Depth	Single predictor	35.47

Table A3.3 – Observed means for environmental variables

Variable	Mean	Standard Deviation
Depth (m)	36.35	26.97211
Distance to nearest patch boundary (Km)	0.1596	0.1939
Seascape Substratum Diversity (Shannon Index)	1.0911	0.3782

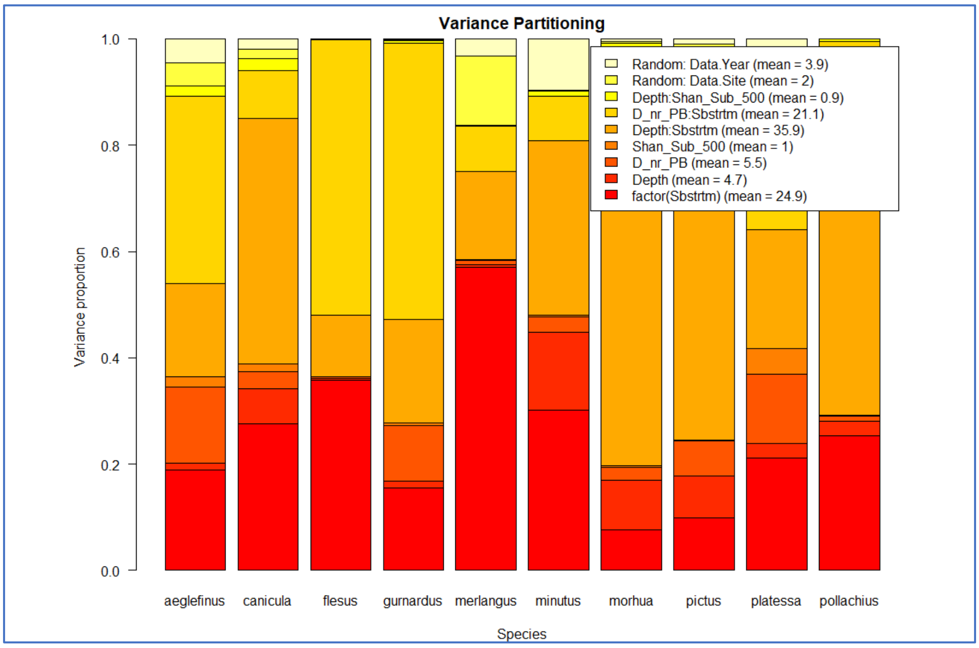

Figure A3.1 – Variance partitioning graph.

Appendix 4 – Model Convergence Plots

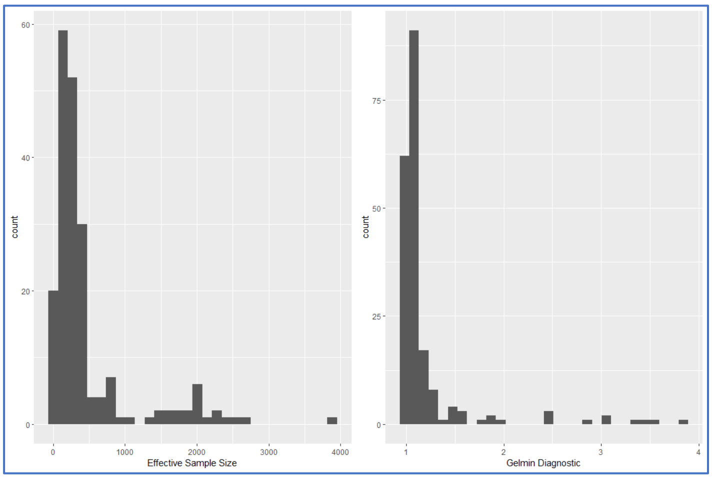

Figure A4.1 – Effective sample size (ESS) and Gelmin Diagnostic (GD) plots. As can be seen the effective sample size is clustered far from the desired sample size; this is acceptable for non-normally distributed data (Tikhonov et al., 2020). The potential scale reduction factor (Gelmin Diagnostic) is centred around 1.01 which indicated the chains gave consistent results.

Appendix 5 – Model Density Plots

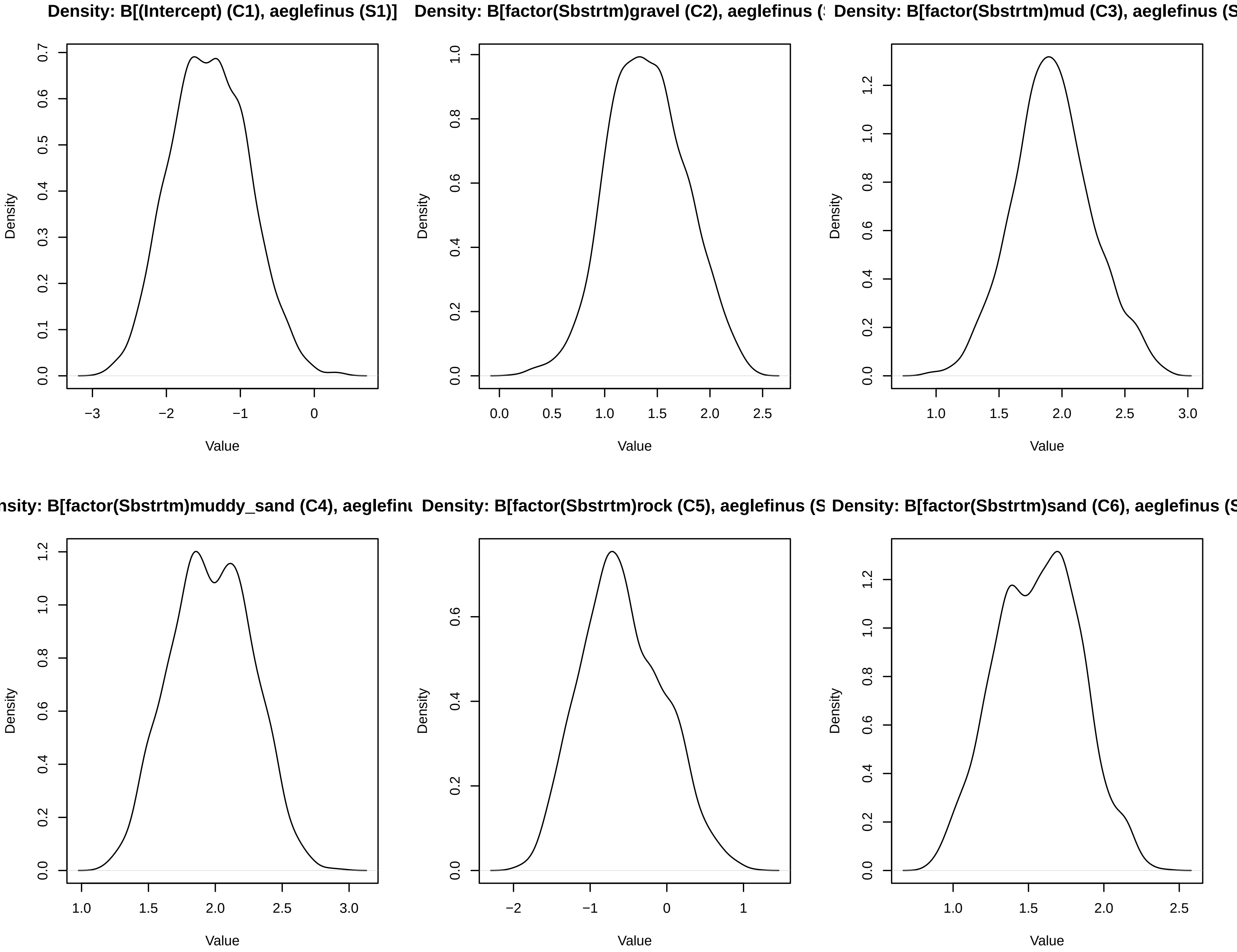

Density: B[(Intercept) (C1), aeglefinus (S1)]
Density: B[factor(Sbstrtm)gravel (C2), aeglefinus (
Density: B[factor(Sbstrtm)mud (C3), aeglefinus (S
nsity: B[factor(Sbstrtm)muddy_sand (C4), aeglefin
Density: B[factor(Sbstrtm)rock (C5), aeglefinus (S
Density: B[factor(Sbstrtm)sand (C6), aeglefinus (S
Density
Value

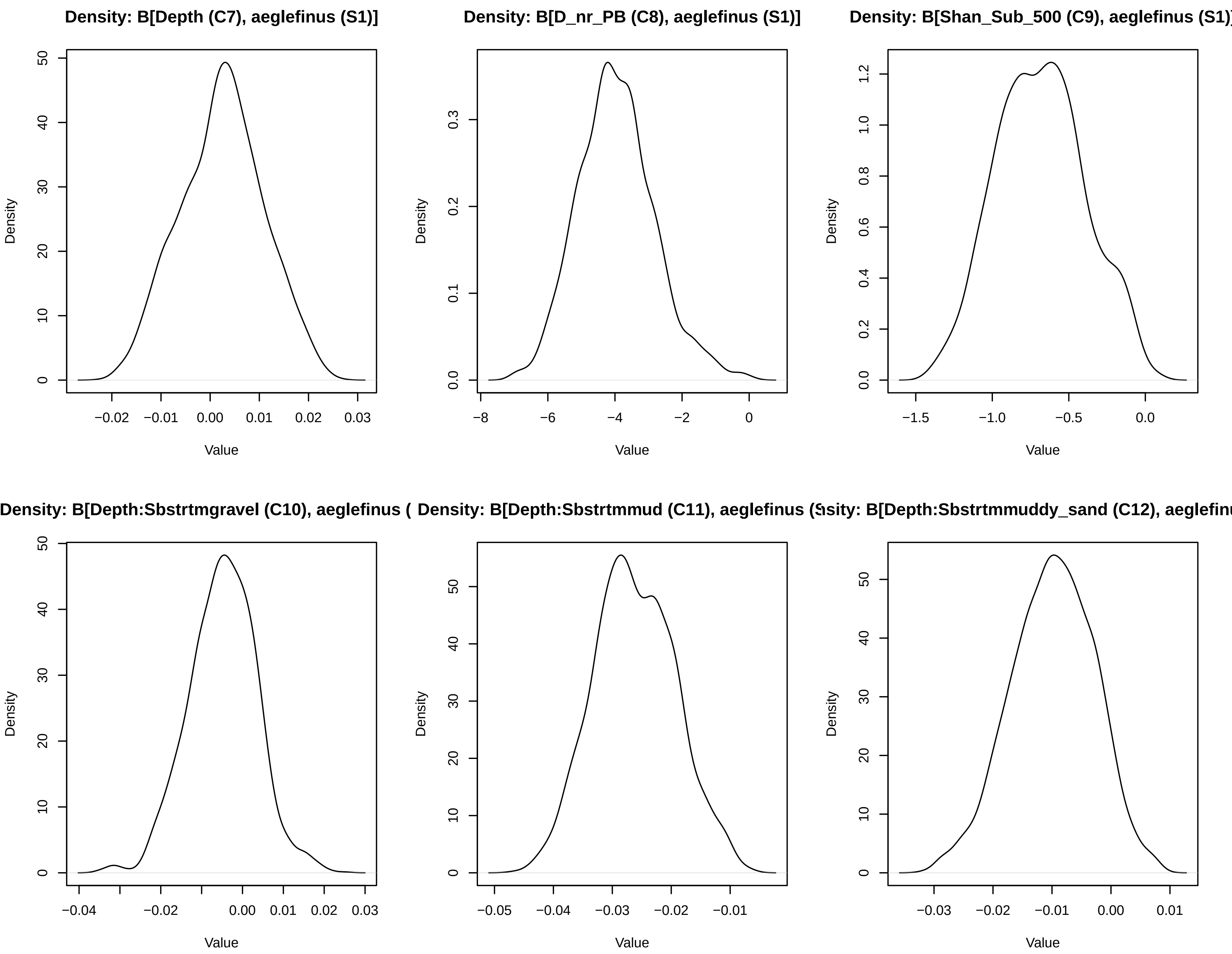

Density: B[Depth (C7), aeglefinus (S1)]
Density: B[D_nr_PB (C8), aeglefinus (S1)]
Density: B[Shan_Sub_500 (C9), aeglefinus (S1)
Density: B[Depth:Sbstrtmgravel (C10), aeglefinus (
Density: B[Depth:Sbstrtmmud (C11), aeglefinus (S
sity: B[Depth:Sbstrtmmuddy_sand (C12), aeglefinu
Density
Value

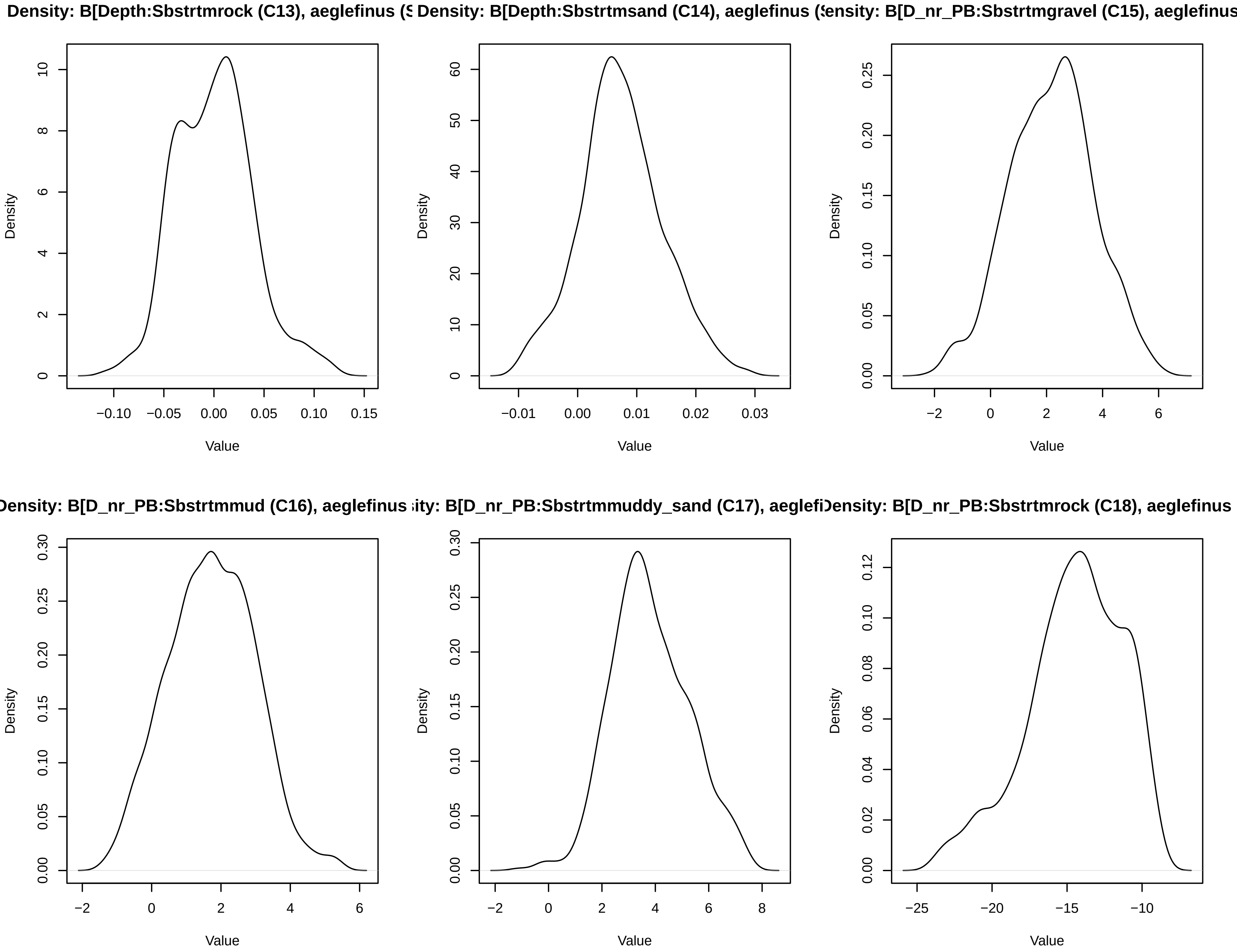
Density: B[Depth:Sbstrtmrock (C13), aeglefinus (S
Density: B[Depth:Sbstrtmsand (C14), aeglefinus (S
ensity: B[D_nr_PB:Sbstrtmgravel (C15), aeglefinus
Density: B[D_nr_PB:Sbstrtmmud (C16), aeglefinus
sity: B[D_nr_PB:Sbstrtmmuddy_sand (C17), aeglefi
Density: B[D_nr_PB:Sbstrtmrock (C18), aeglefinus
Density
Value

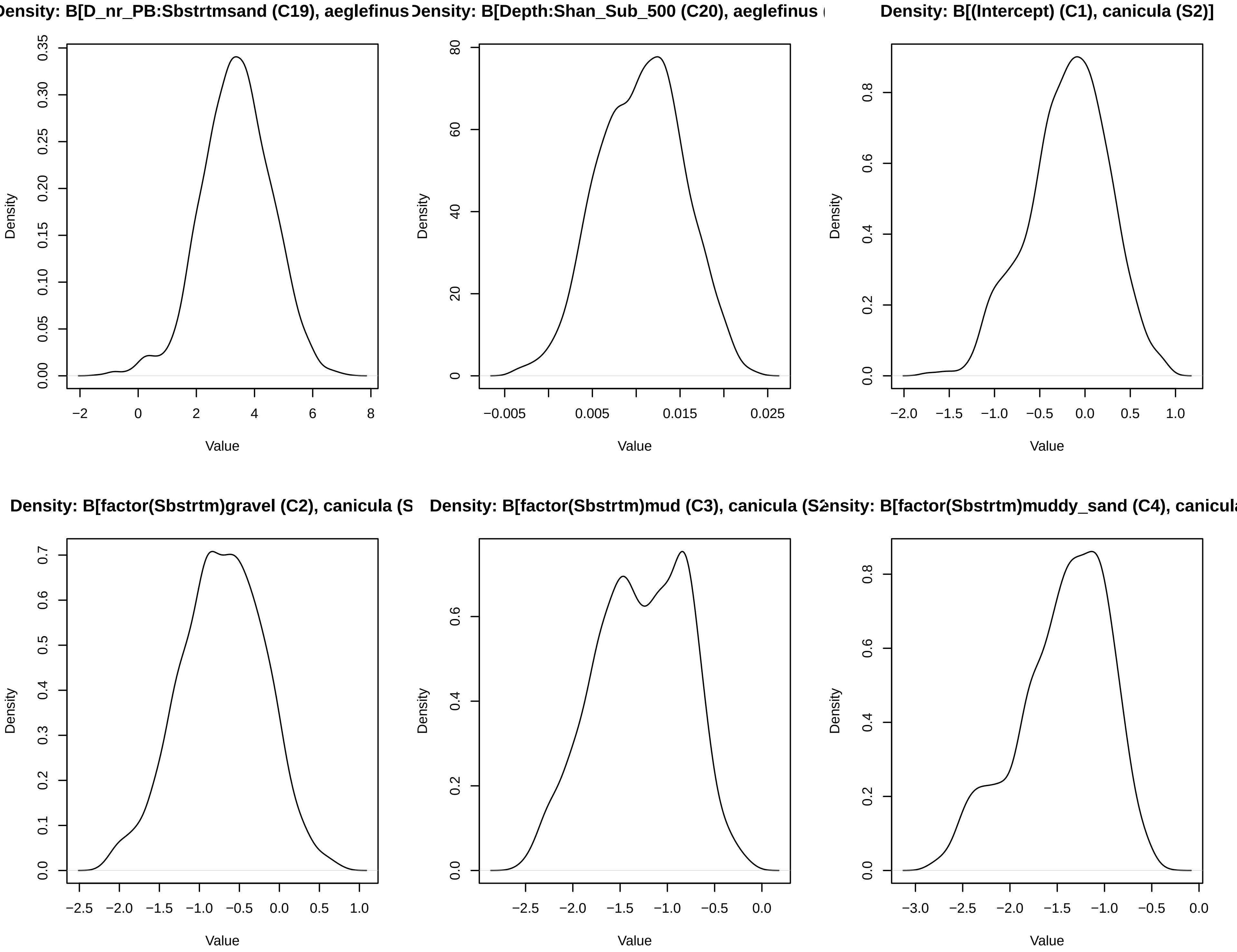

Density: B[D_nr_PB:Sbstrtmsand (C19), aeglefinus
Density: B[Depth:Shan_Sub_500 (C20), aeglefinus (
Density: B[(Intercept) (C1), canicula (S2)]
Density: B[factor(Sbstrtm)gravel (C2), canicula (S
Density: B[factor(Sbstrtm)mud (C3), canicula (S2
nsity: B[factor(Sbstrtm)muddy_sand (C4), canicula
Density
Value

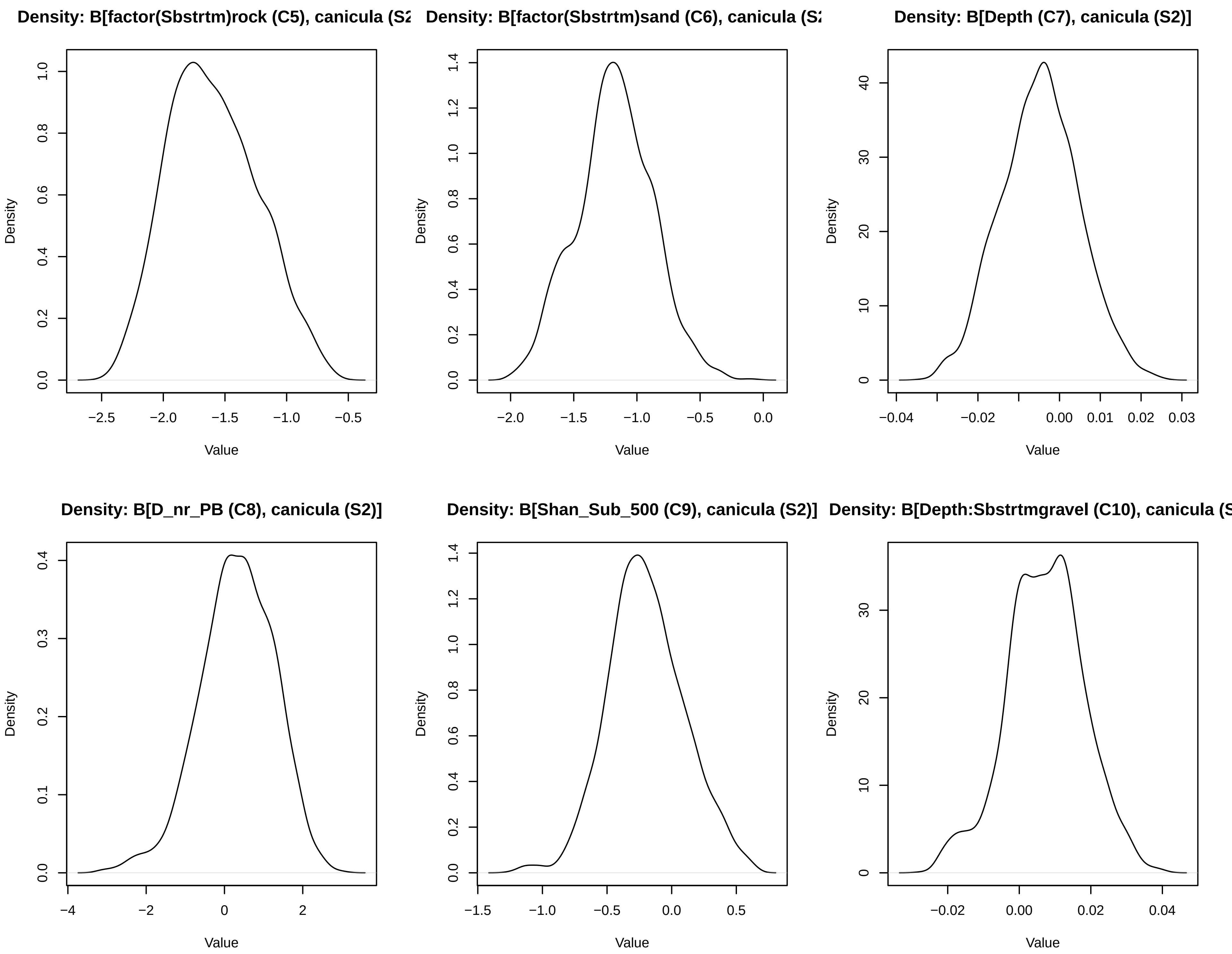
Density: B[factor(Sbstrtm)rock (C5), canicula (S2
Density: B[factor(Sbstrtm)sand (C6), canicula (S2
Density: B[Depth (C7), canicula (S2)]
Density: B[D_nr_PB (C8), canicula (S2)]
Density: B[Shan_Sub_500 (C9), canicula (S2)]
Density: B[Depth:Sbstrtmgravel (C10), canicula (S
Density
Value
0.0
0.2
0.4
0.6
0.8
1.0
1.2
1.4
−2.5
−2.0
−1.5
−1.0
−0.5
0.0
0
10
20
30
40
−0.04
−0.02
0.00
0.01
0.02
0.03
0.1
0.3
−4
−2
2
0.5
0.04

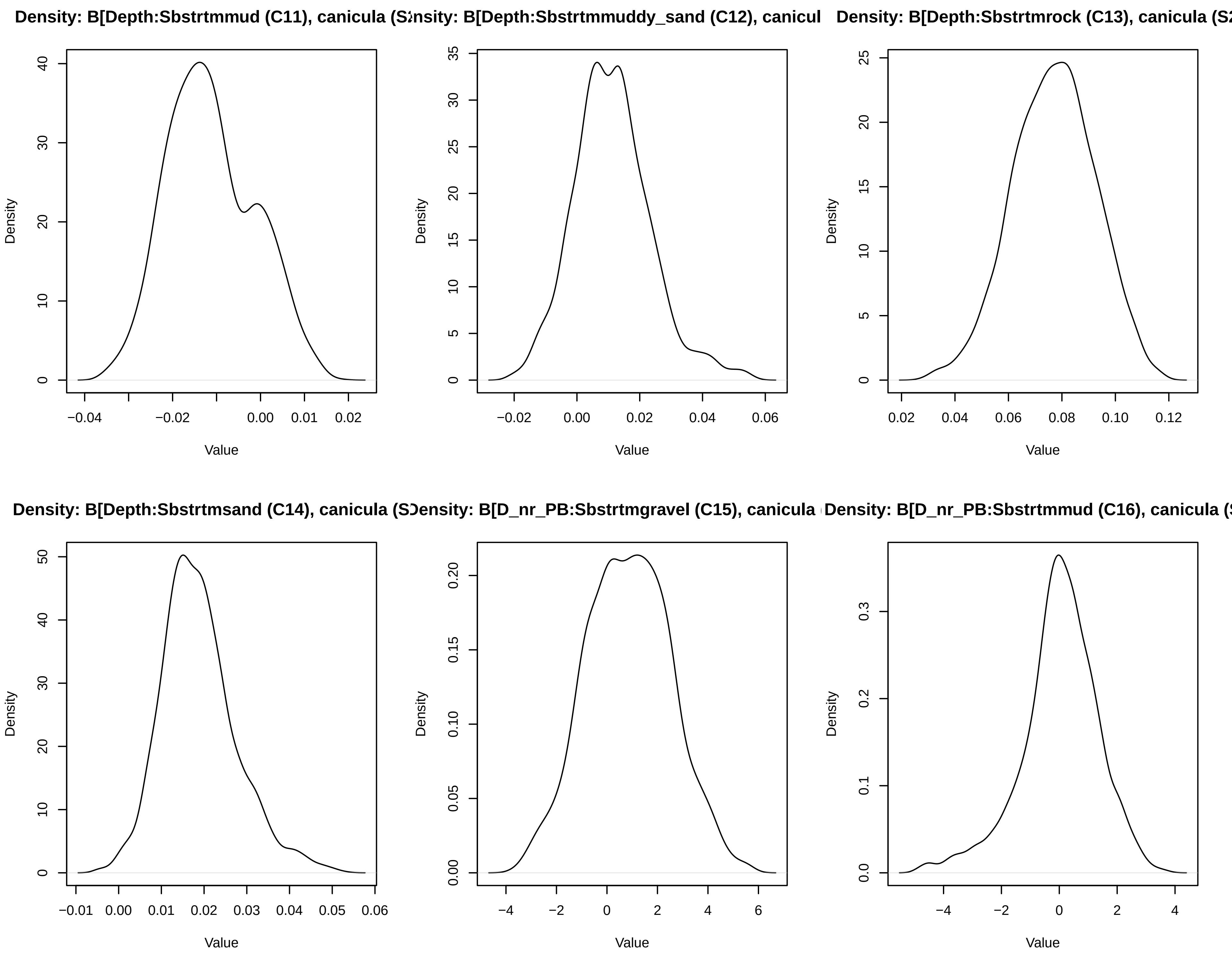

Density: B[Depth:Sbstrtmmud (C11), canicula (S
Density
Value
Density: B[Depth:Sbstrtmmuddy_sand (C12), canicul
Density
Value
Density: B[Depth:Sbstrtmrock (C13), canicula (S
Density
Value
Density: B[Depth:Sbstrtmsand (C14), canicula (S
Density
Value
Density: B[D_nr_PB:Sbstrtmgravel (C15), canicula
Density
Value
Density: B[D_nr_PB:Sbstrtmmud (C16), canicula (S
Density
Value

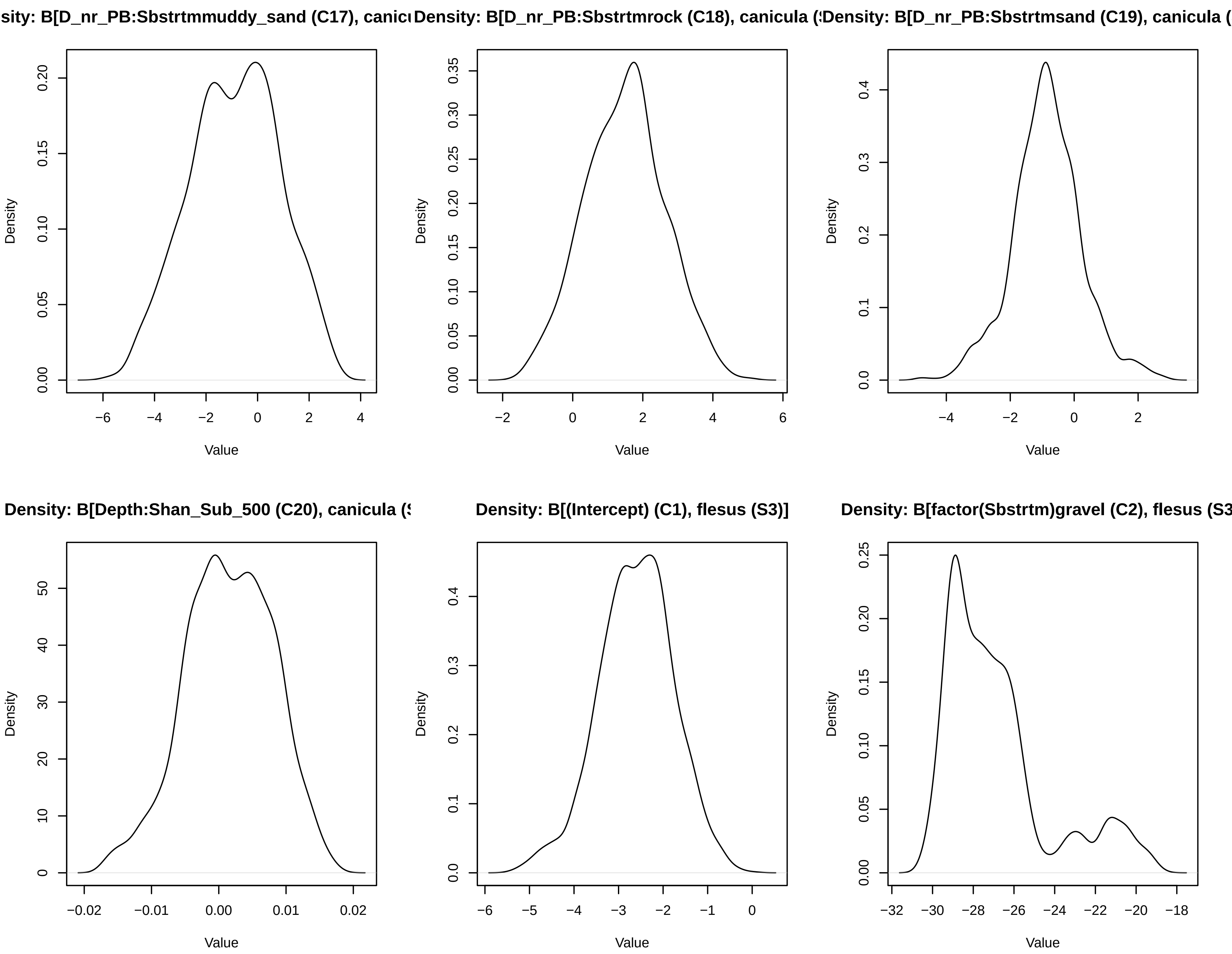
sity: B[D_nr_PB:Sbstrtmmuddy_sand (C17), canic
Density: B[D_nr_PB:Sbstrtmrock (C18), canicula (S
Density: B[D_nr_PB:Sbstrtmsand (C19), canicula (
Density: B[Depth:Shan_Sub_500 (C20), canicula (S
Density: B[(Intercept) (C1), flesus (S3)]
Density: B[factor(Sbstrtm)gravel (C2), flesus (S3
Density
Value

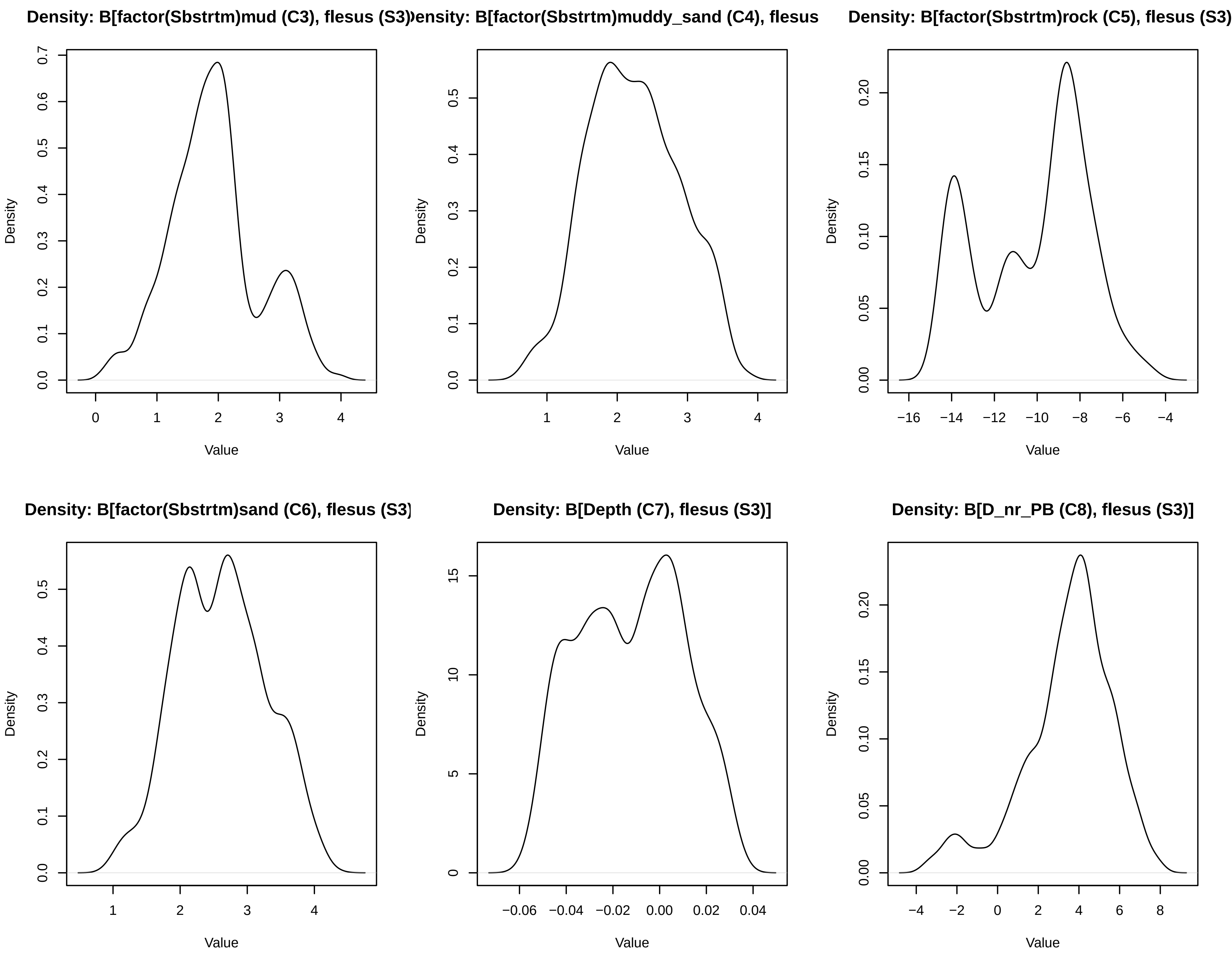
Density: B[factor(Sbstrtm)mud (C3), flesus (S3)]
Density: B[factor(Sbstrtm)muddy_sand (C4), flesus
Density: B[factor(Sbstrtm)rock (C5), flesus (S3)]
Density: B[factor(Sbstrtm)sand (C6), flesus (S3)]
Density: B[Depth (C7), flesus (S3)]
Density: B[D_nr_PB (C8), flesus (S3)]
Density
Value

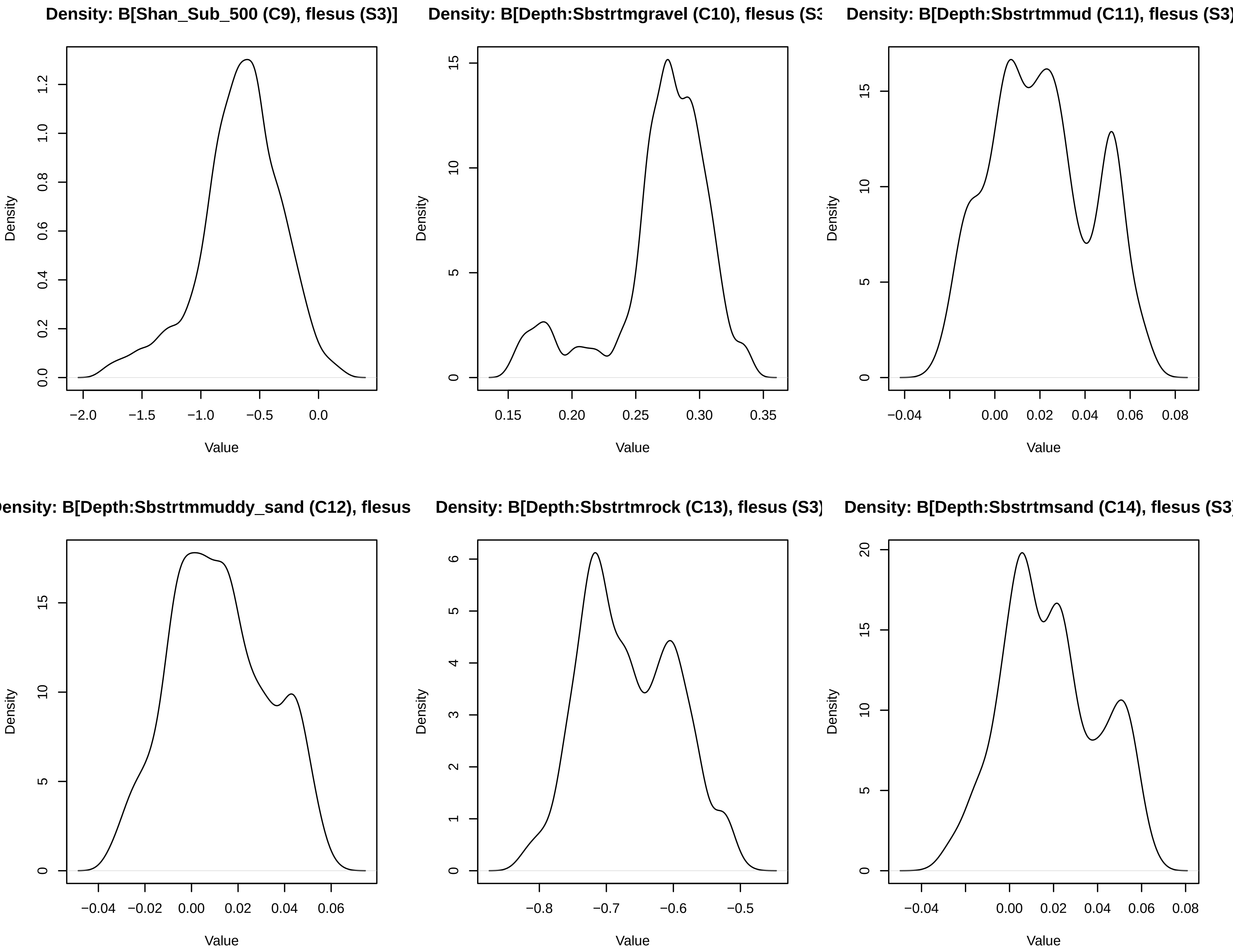

Density: B[Shan_Sub_500 (C9), flesus (S3)]
Density: B[Depth:Sbstrtmgravel (C10), flesus (S3
Density: B[Depth:Sbstrtmmud (C11), flesus (S3)
ensity: B[Depth:Sbstrtmmuddy_sand (C12), flesus
Density: B[Depth:Sbstrtmrock (C13), flesus (S3)
Density: B[Depth:Sbstrtmsand (C14), flesus (S3)
Density
Value

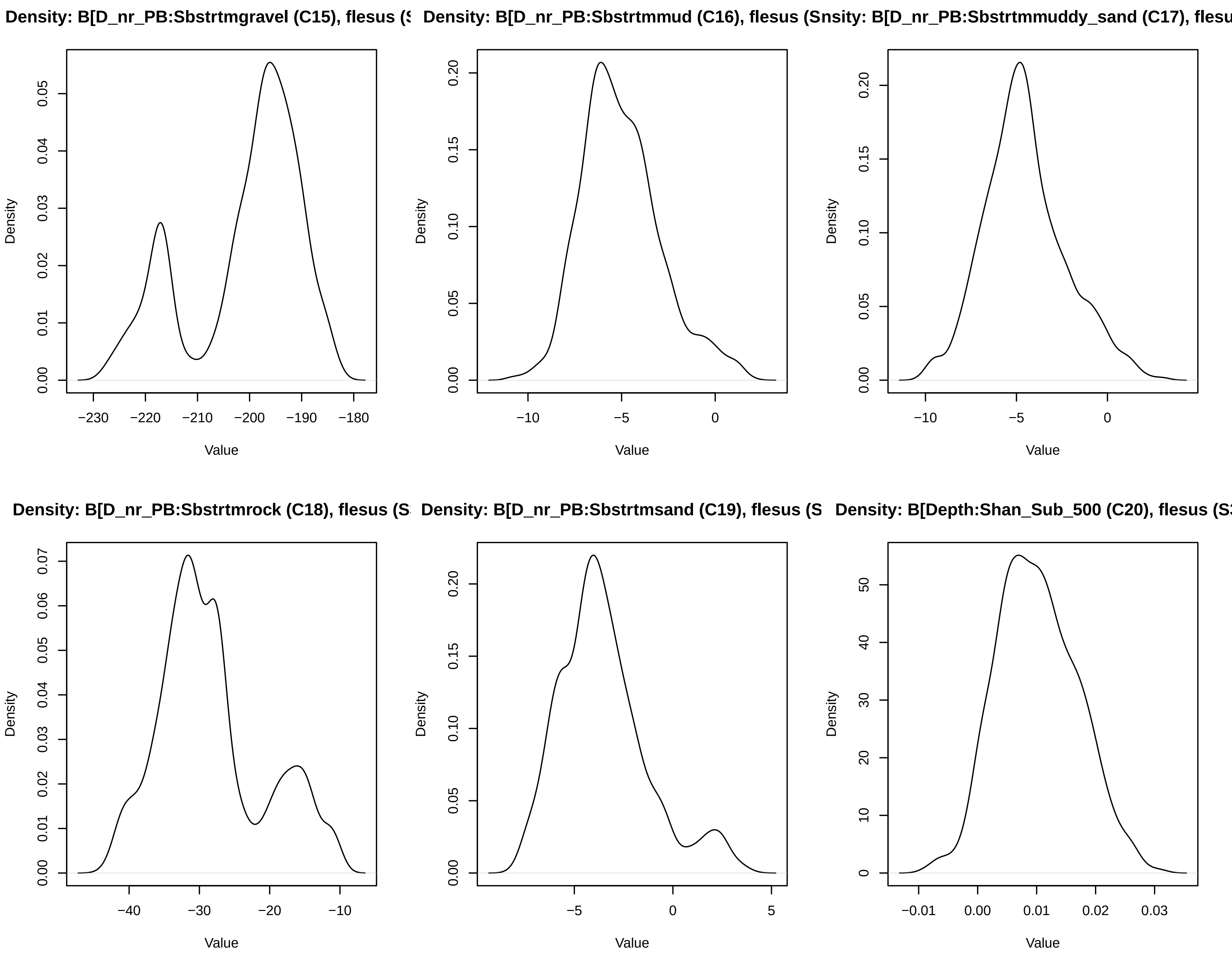

Density: B[D_nr_PB:Sbstrtmgravel (C15), flesus (S
Density: B[D_nr_PB:Sbstrtmmud (C16), flesus (S
nsity: B[D_nr_PB:Sbstrtmmuddy_sand (C17), flesu
Density: B[D_nr_PB:Sbstrtmrock (C18), flesus (S
Density: B[D_nr_PB:Sbstrtmsand (C19), flesus (S
Density: B[Depth:Shan_Sub_500 (C20), flesus (S
Density
Value
0.00
0.01
0.02
0.03
0.04
0.05
−230
−220
−210
−200
−190
−180
0.05
0.10
0.15
0.20
−10
−5
0
5
0.06
0.07
−40
−30
−20
−10
10
20
30
40
50
−0.01
0.00
0.01
0.02
0.03

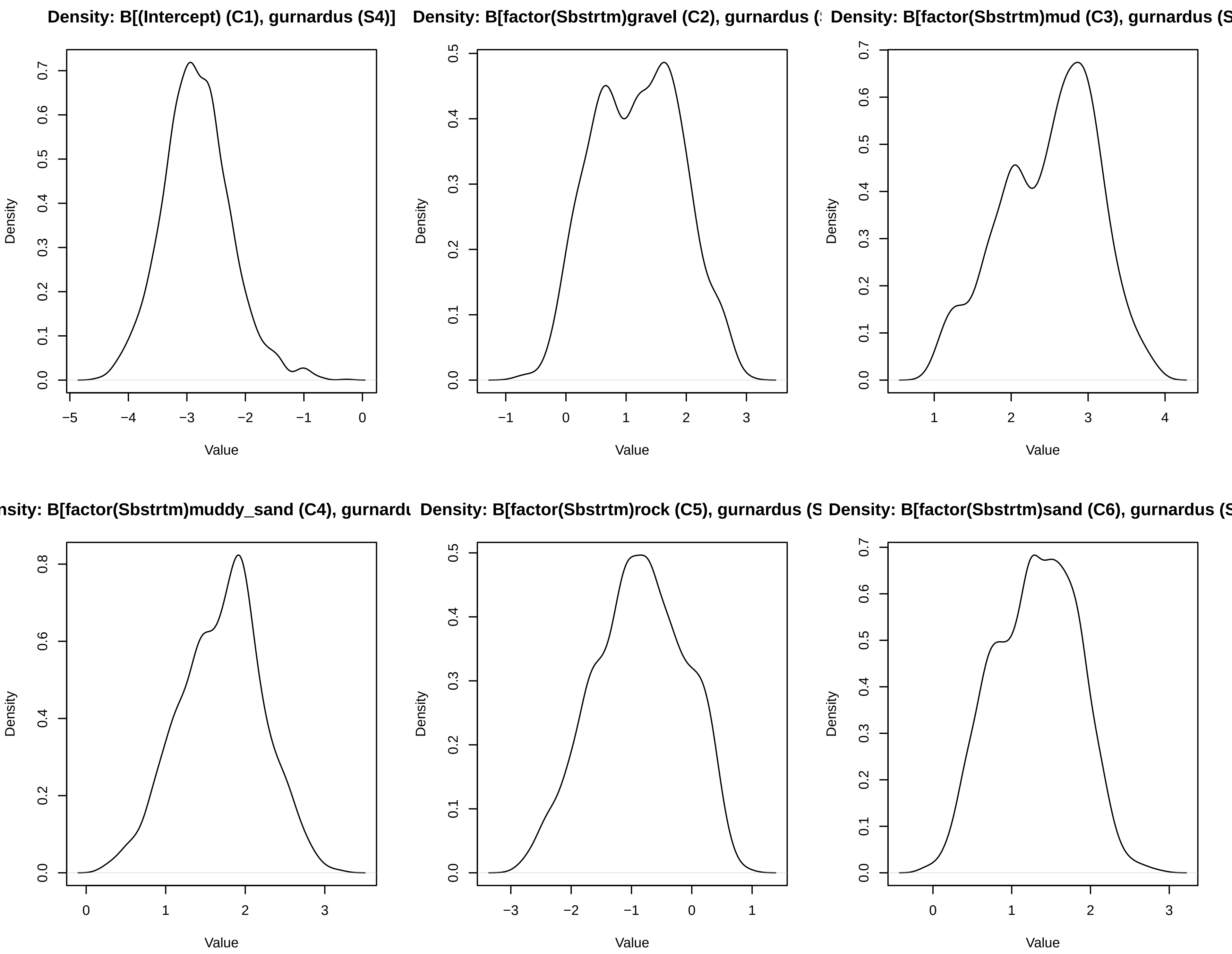
Density: B[(Intercept) (C1), gurnardus (S4)]
Density: B[factor(Sbstrtm)gravel (C2), gurnardus (
Density: B[factor(Sbstrtm)mud (C3), gurnardus (S
nsity: B[factor(Sbstrtm)muddy_sand (C4), gurnardu
Density: B[factor(Sbstrtm)rock (C5), gurnardus (S
Density: B[factor(Sbstrtm)sand (C6), gurnardus (S
Density
Value

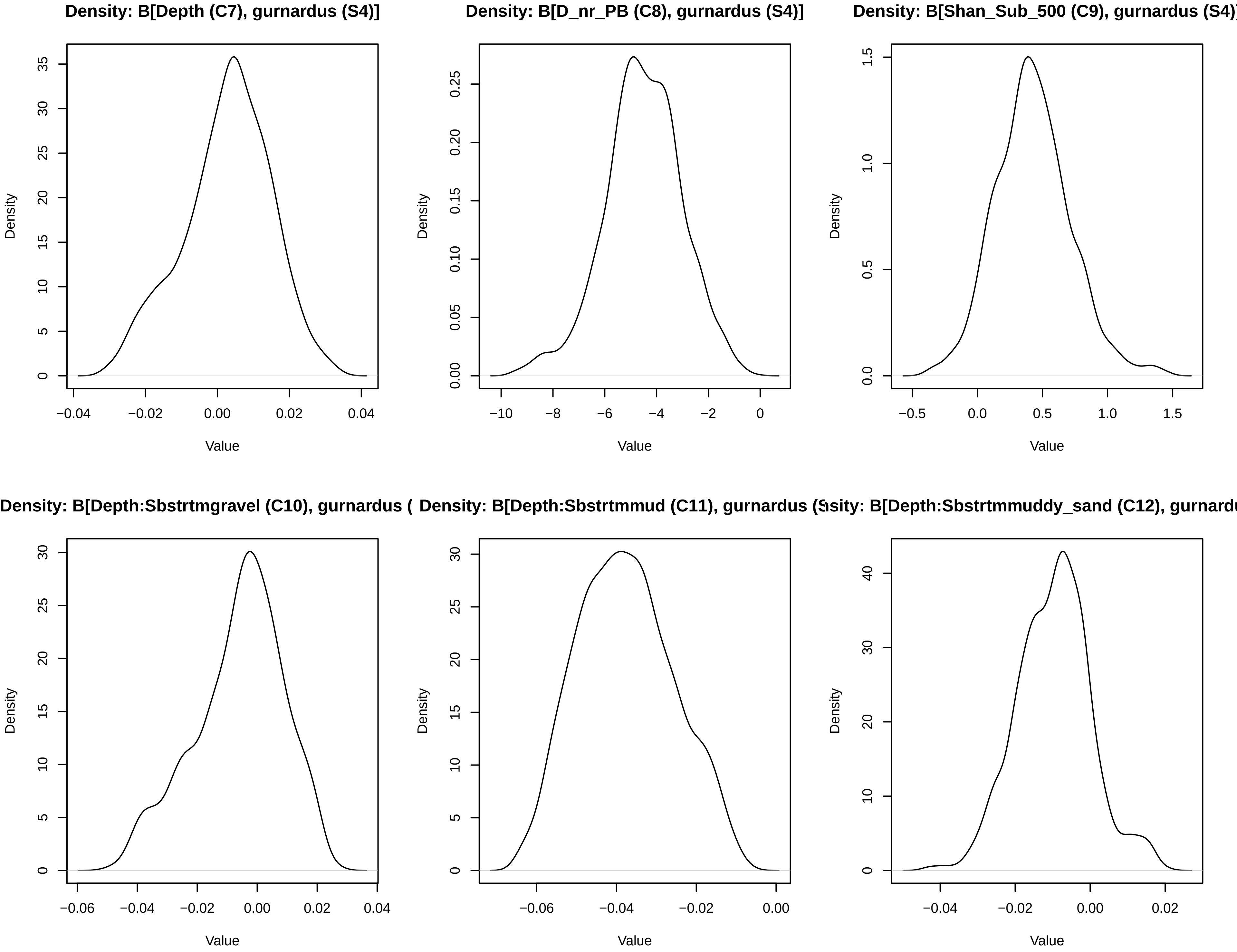
Density: B[Depth (C7), gurnardus (S4)]
Density
0 5 10 15 20 25 30 35
−0.04 −0.02 0.00 0.02 0.04
Value
Density: B[D_nr_PB (C8), gurnardus (S4)]
Density
0.00 0.05 0.10 0.15 0.20 0.25
−10 −8 −6 −4 −2 0
Value
Density: B[Shan_Sub_500 (C9), gurnardus (S4)
Density
0.0 0.5 1.0 1.5
−0.5 0.0 0.5 1.0 1.5
Value
Density: B[Depth:Sbstrtmgravel (C10), gurnardus (
Density
0 5 10 15 20 25 30
−0.06 −0.04 −0.02 0.00 0.02 0.04
Value
Density: B[Depth:Sbstrtmmud (C11), gurnardus (
Density
0 5 10 15 20 25 30
−0.06 −0.04 −0.02 0.00
Value
sity: B[Depth:Sbstrtmmuddy_sand (C12), gurnardu
Density
0 10 20 30 40
−0.04 −0.02 0.00 0.02
Value

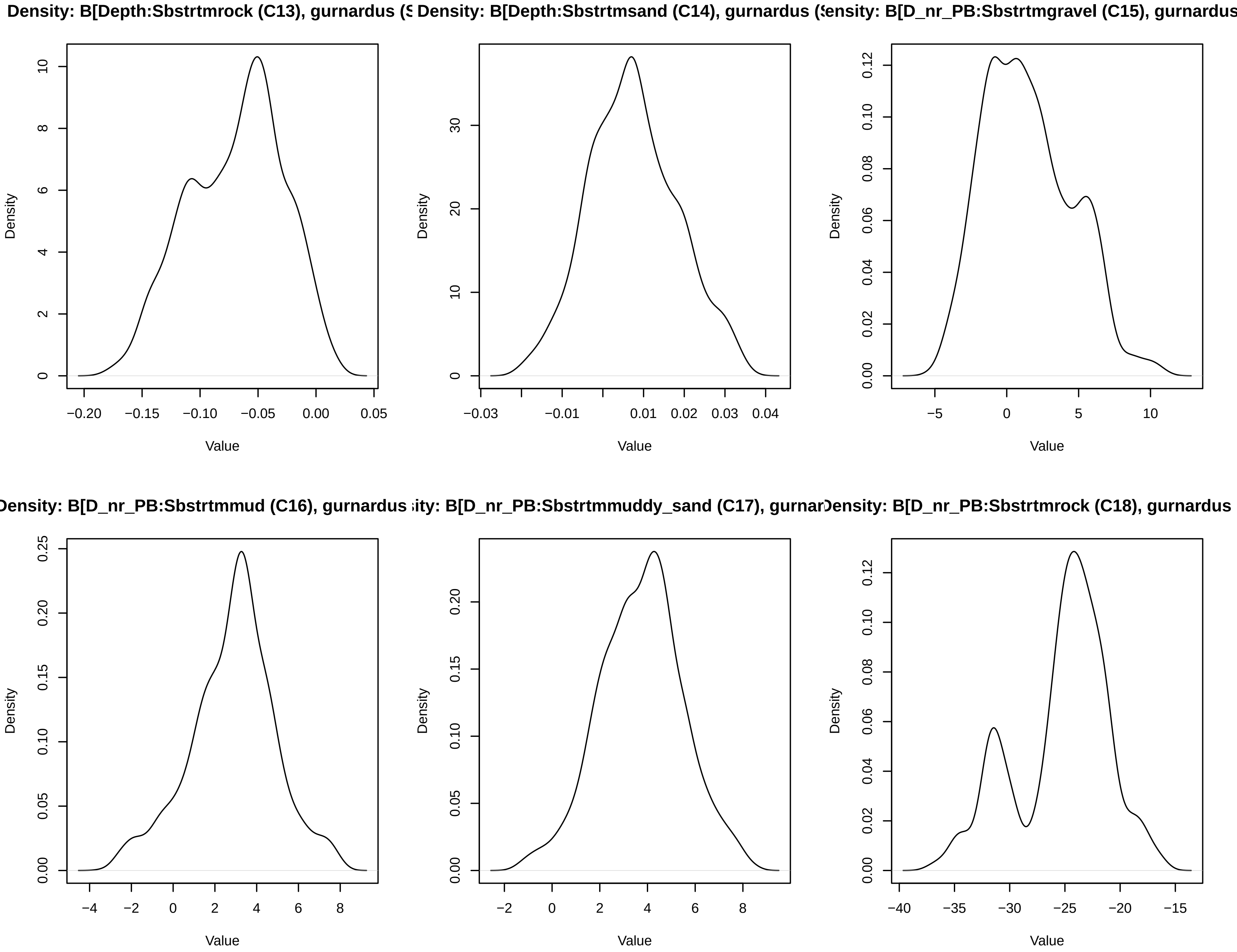
Density: B[Depth:Sbstrtmrock (C13), gurnardus (S
Density: B[Depth:Sbstrtmsand (C14), gurnardus (S
ensity: B[D_nr_PB:Sbstrtmgravel (C15), gurnardus
Density: B[D_nr_PB:Sbstrtmmud (C16), gurnardus
ity: B[D_nr_PB:Sbstrtmmuddy_sand (C17), gurnar
Density: B[D_nr_PB:Sbstrtmrock (C18), gurnardus
Density
Value
−0.20 −0.15 −0.10 −0.05 0.00 0.05
−0.03 −0.01 0.01 0.02 0.03 0.04
−5 0 5 10
−4 −2 0 2 4 6 8
−2 0 2 4 6 8
−40 −35 −30 −25 −20 −15

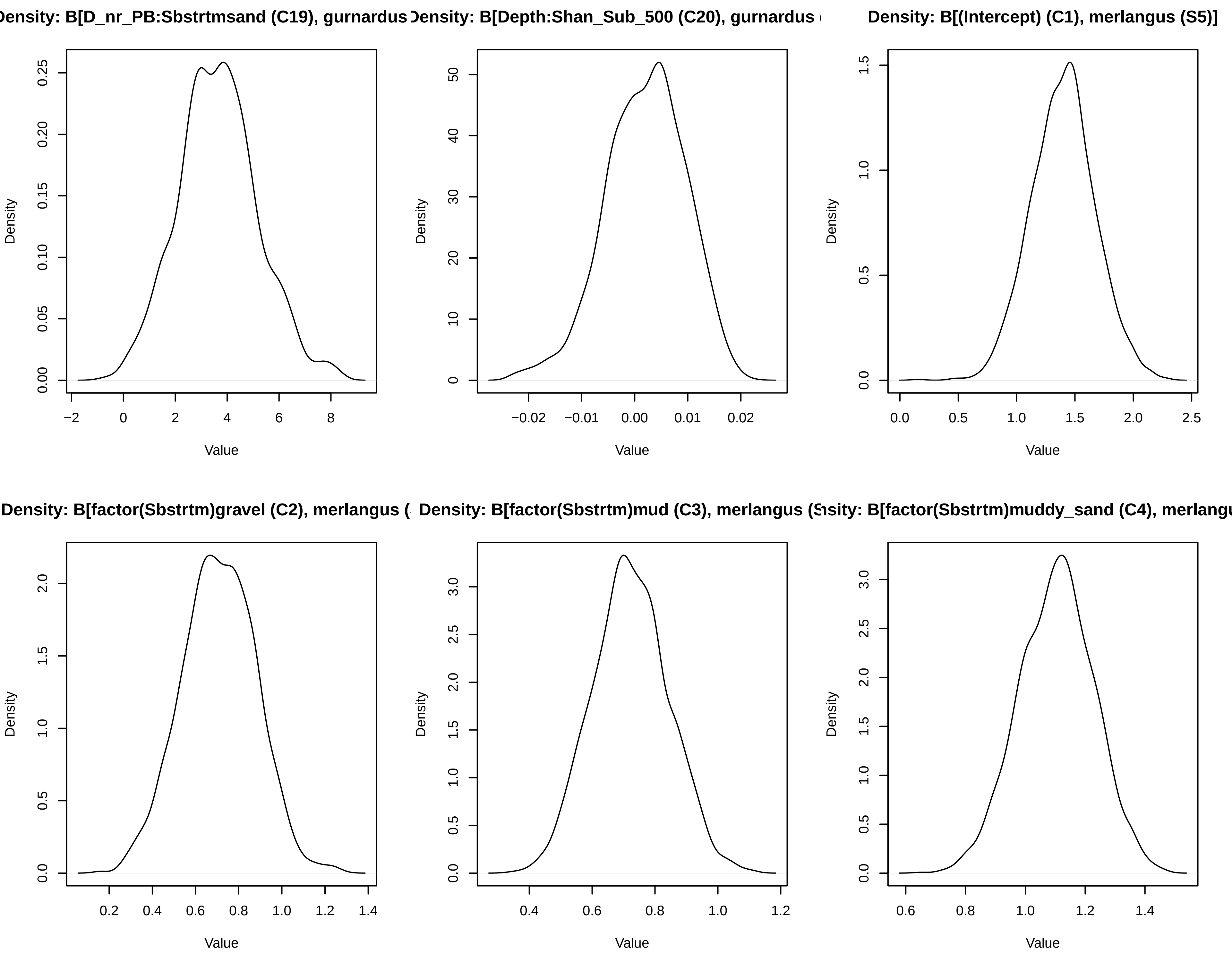
Density: B[D_nr_PB:Sbstrtmsand (C19), gurnardus
Density
Value
Density: B[Depth:Shan_Sub_500 (C20), gurnardus (
Density
Value
Density: B[(Intercept) (C1), merlangus (S5)]
Density
Value
Density: B[factor(Sbstrtm)gravel (C2), merlangus (
Density
Value
Density: B[factor(Sbstrtm)mud (C3), merlangus (S
Density
Value
nsity: B[factor(Sbstrtm)muddy_sand (C4), merlangu
Density
Value

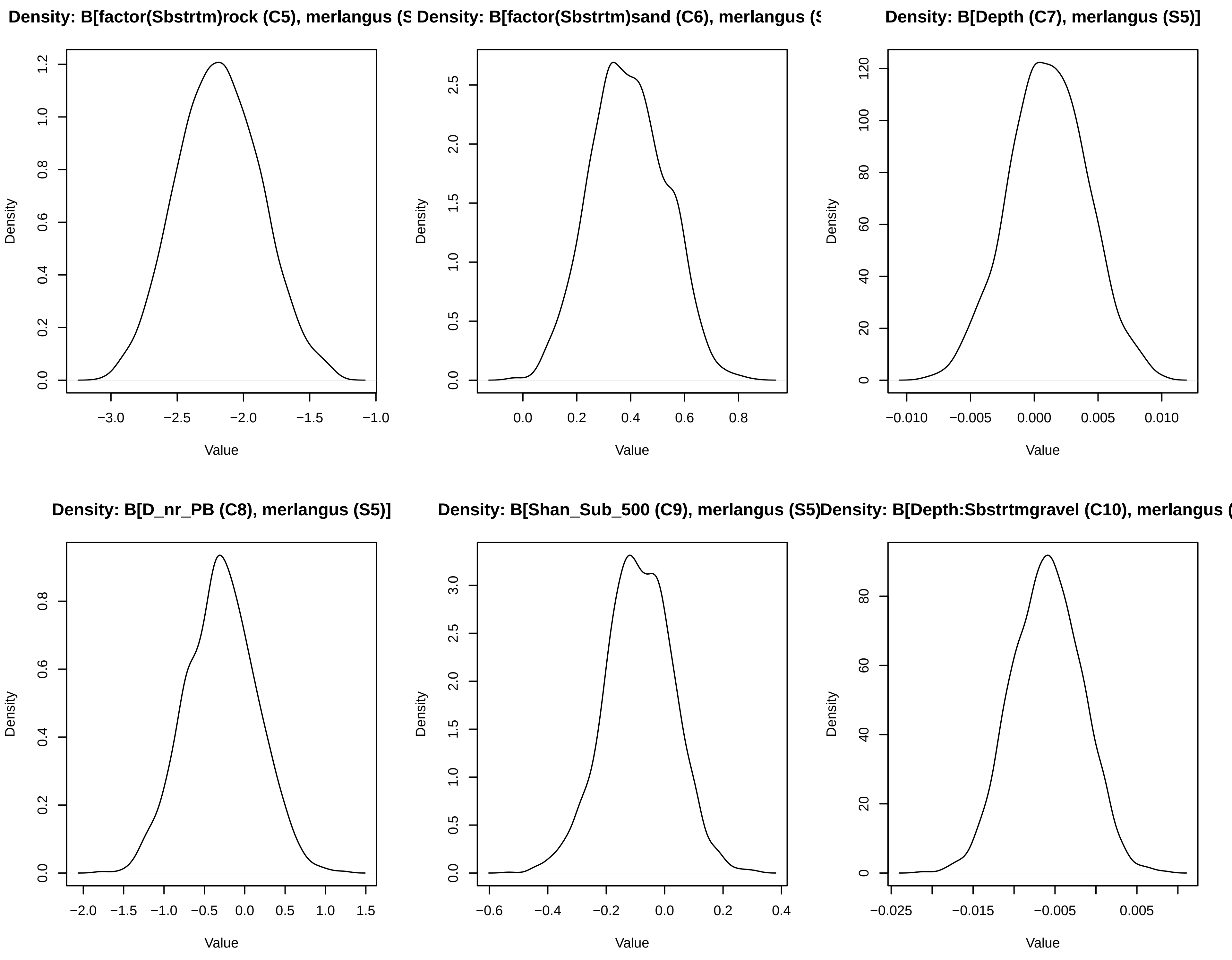
Density: B[factor(Sbstrtm)rock (C5), merlangus (S
Density: B[factor(Sbstrtm)sand (C6), merlangus (S
Density: B[Depth (C7), merlangus (S5)]
Density: B[D_nr_PB (C8), merlangus (S5)]
Density: B[Shan_Sub_500 (C9), merlangus (S5)]
Density: B[Depth:Sbstrtmgravel (C10), merlangus (
Density
Value
−3.0 −2.5 −2.0 −1.5 −1.0
0.0 0.2 0.4 0.6 0.8 1.0 1.2
0.0 0.2 0.4 0.6 0.8
0.0 0.5 1.0 1.5 2.0 2.5
−0.010 −0.005 0.000 0.005 0.010
0 20 40 60 80 100 120
−2.0 −1.5 −1.0 −0.5 0.0 0.5 1.0 1.5
−0.6 −0.4 −0.2 0.0 0.2 0.4
0.0 0.5 1.0 1.5 2.0 2.5 3.0
−0.025 −0.015 −0.005 0.005
0 20 40 60 80

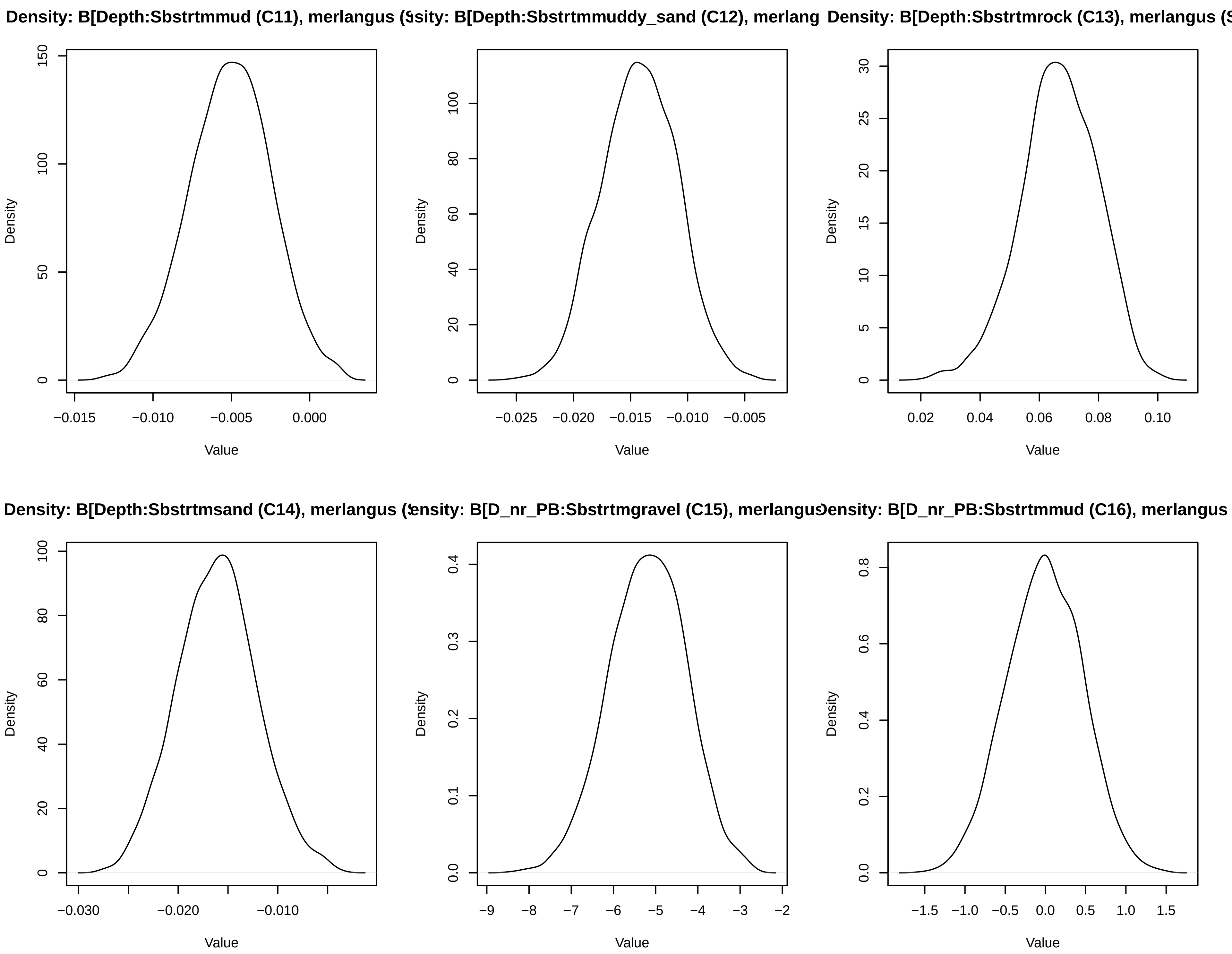
Density: B[Depth:Sbstrtmmud (C11), merlangus (S
sity: B[Depth:Sbstrtmmuddy_sand (C12), merlang
Density: B[Depth:Sbstrtmrock (C13), merlangus (S
Density: B[Depth:Sbstrtmsand (C14), merlangus (S
ensity: B[D_nr_PB:Sbstrtmgravel (C15), merlangus
ensity: B[D_nr_PB:Sbstrtmmud (C16), merlangus
Density
Value

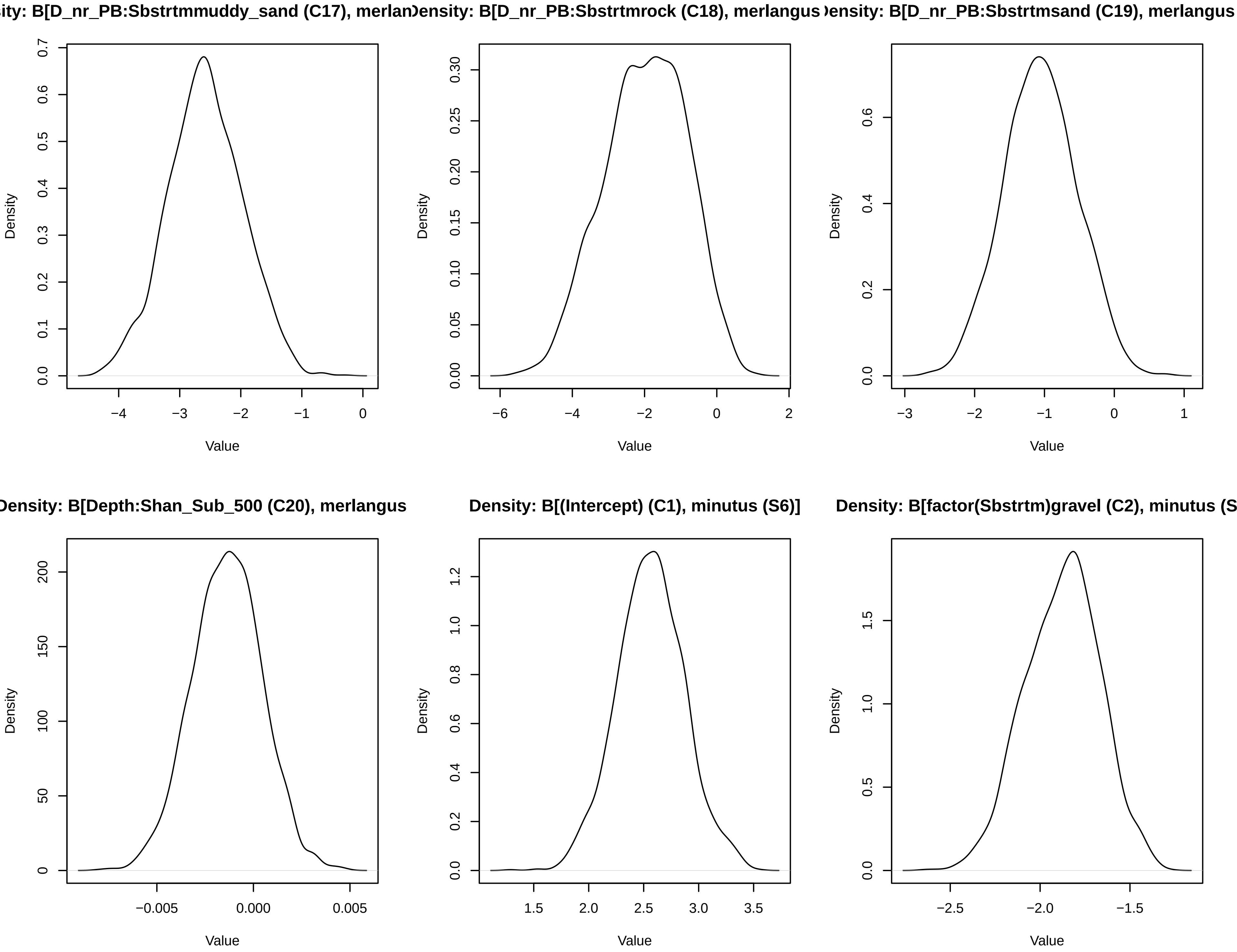
sity: B[D_nr_PB:Sbstrtmmuddy_sand (C17), merlan
ensity: B[D_nr_PB:Sbstrtmrock (C18), merlangus
ensity: B[D_nr_PB:Sbstrtmsand (C19), merlangus
Density: B[Depth:Shan_Sub_500 (C20), merlangus
Density: B[(Intercept) (C1), minutus (S6)]
Density: B[factor(Sbstrtm)gravel (C2), minutus (S
Density
Value

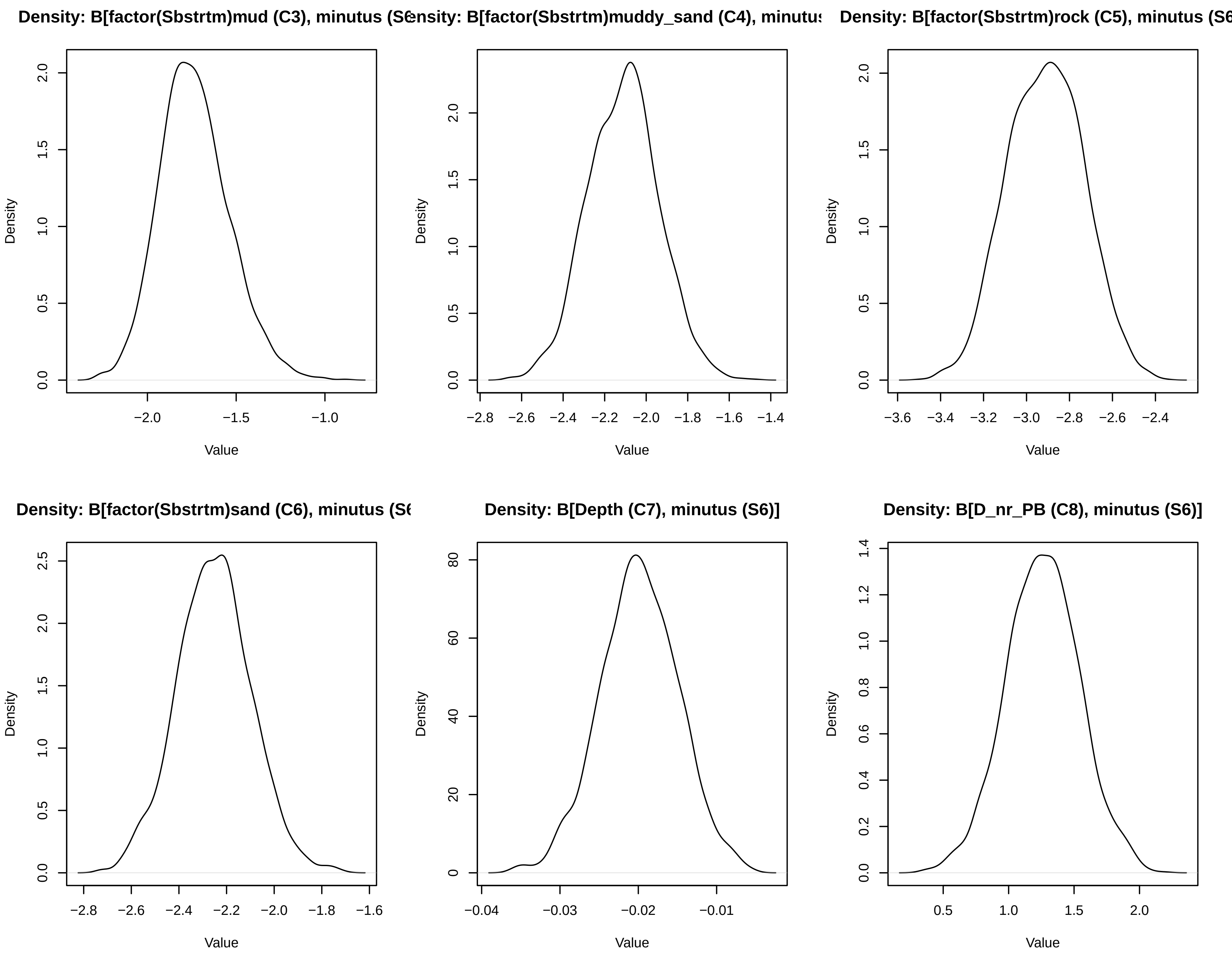

Density: B[factor(Sbstrtm)mud (C3), minutus (S6
Density
Value
ensity: B[factor(Sbstrtm)muddy_sand (C4), minutus
Density
Value
Density: B[factor(Sbstrtm)rock (C5), minutus (S6
Density
Value
Density: B[factor(Sbstrtm)sand (C6), minutus (S6
Density
Value
Density: B[Depth (C7), minutus (S6)]
Density
Value
Density: B[D_nr_PB (C8), minutus (S6)]
Density
Value

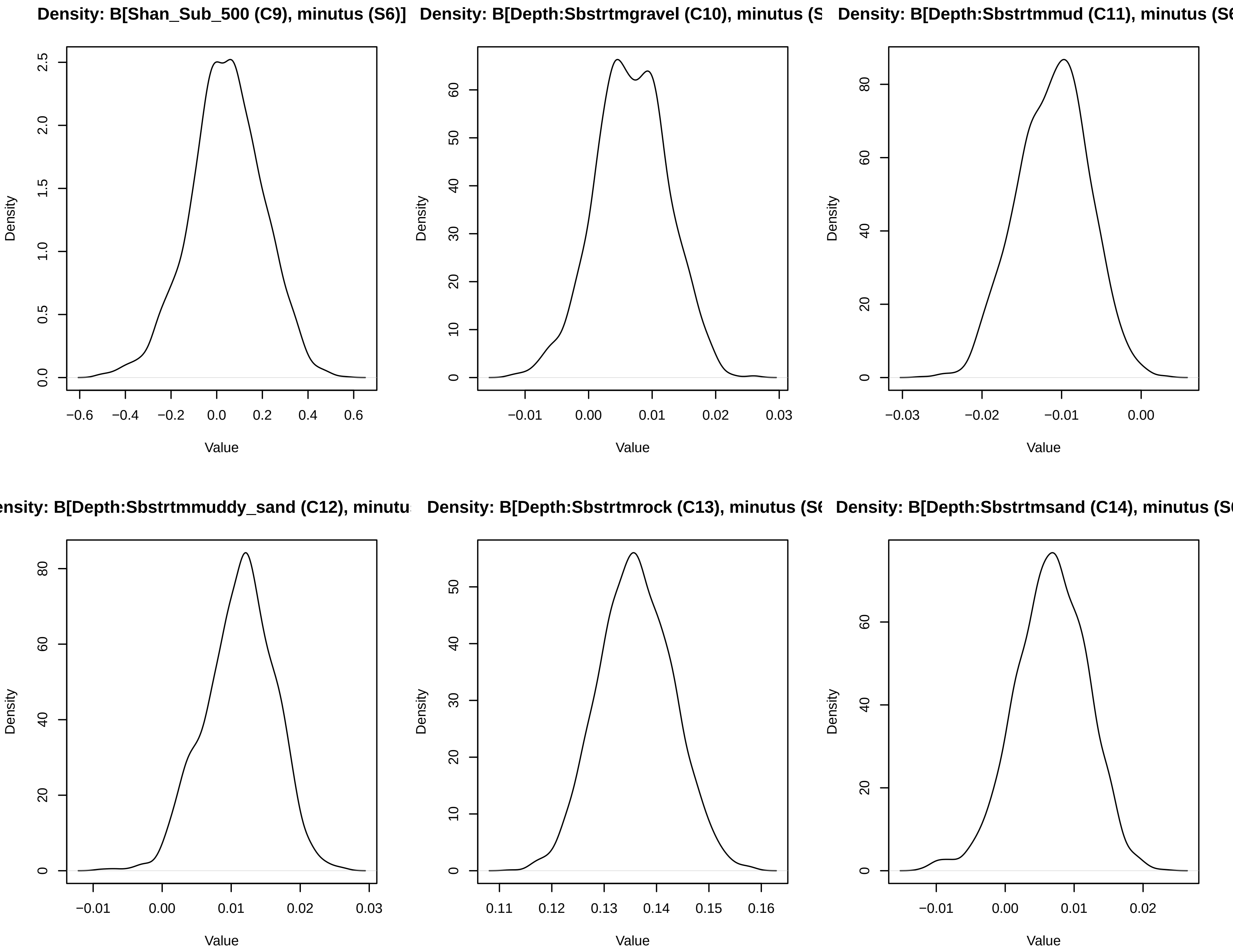

Density: B[Shan_Sub_500 (C9), minutus (S6)]
Density: B[Depth:Sbstrtmgravel (C10), minutus (S
Density: B[Depth:Sbstrtmmud (C11), minutus (S6
ensity: B[Depth:Sbstrtmmuddy_sand (C12), minutus
Density: B[Depth:Sbstrtmrock (C13), minutus (S6
Density: B[Depth:Sbstrtmsand (C14), minutus (S6
Density
Value

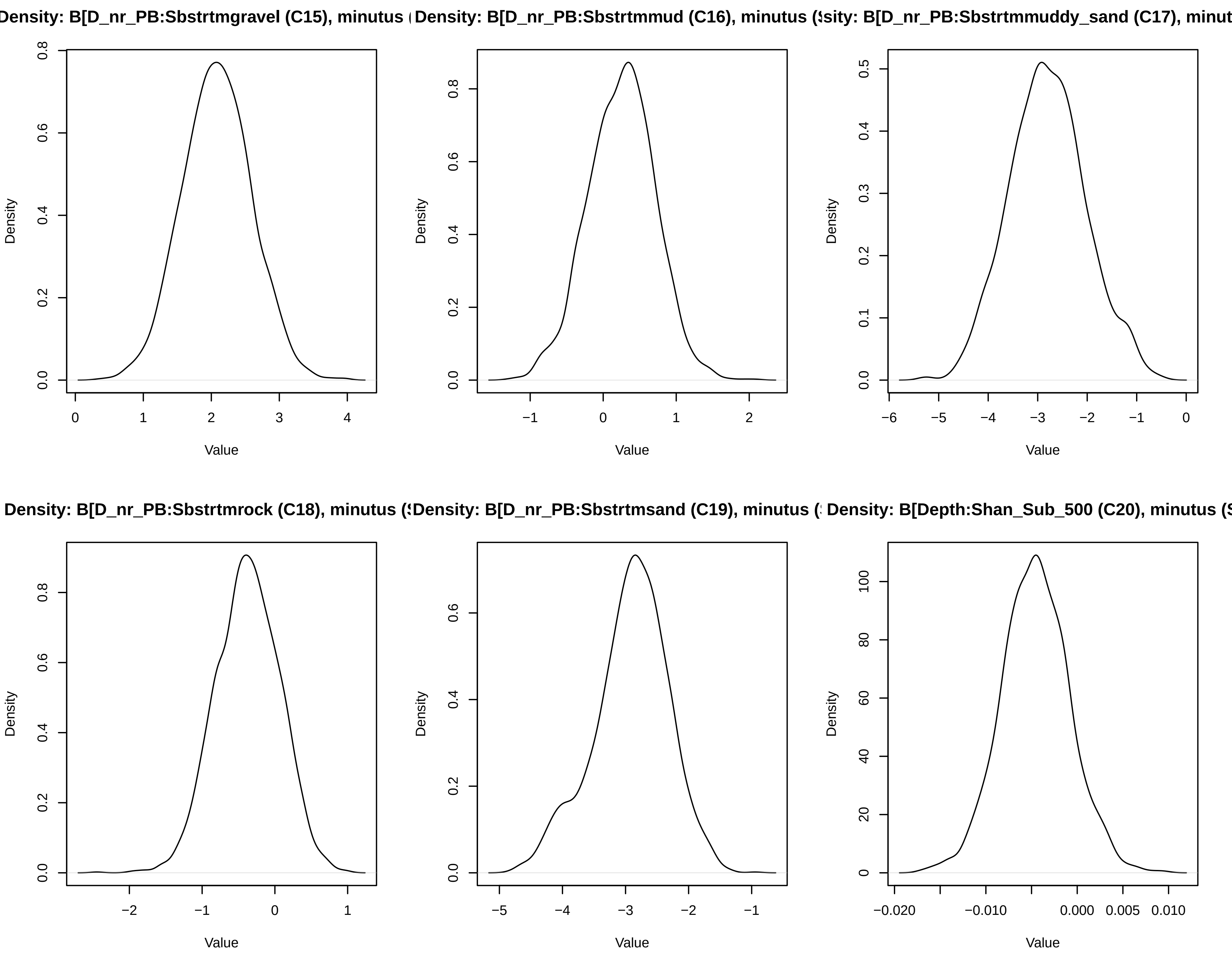

Density: B[D_nr_PB:Sbstrtmgravel (C15), minutus (
Density: B[D_nr_PB:Sbstrtmmud (C16), minutus (S
sity: B[D_nr_PB:Sbstrtmmuddy_sand (C17), minut
Density: B[D_nr_PB:Sbstrtmrock (C18), minutus (S
Density: B[D_nr_PB:Sbstrtmsand (C19), minutus (S
Density: B[Depth:Shan_Sub_500 (C20), minutus (S
Density
Value
0.0
0.2
0.4
0.6
0.8
0
1
2
3
4
−1
−6
−5
−4
−3
−2
0.1
0.3
0.5
100
80
60
40
20
−0.020
−0.010
0.000
0.005
0.010

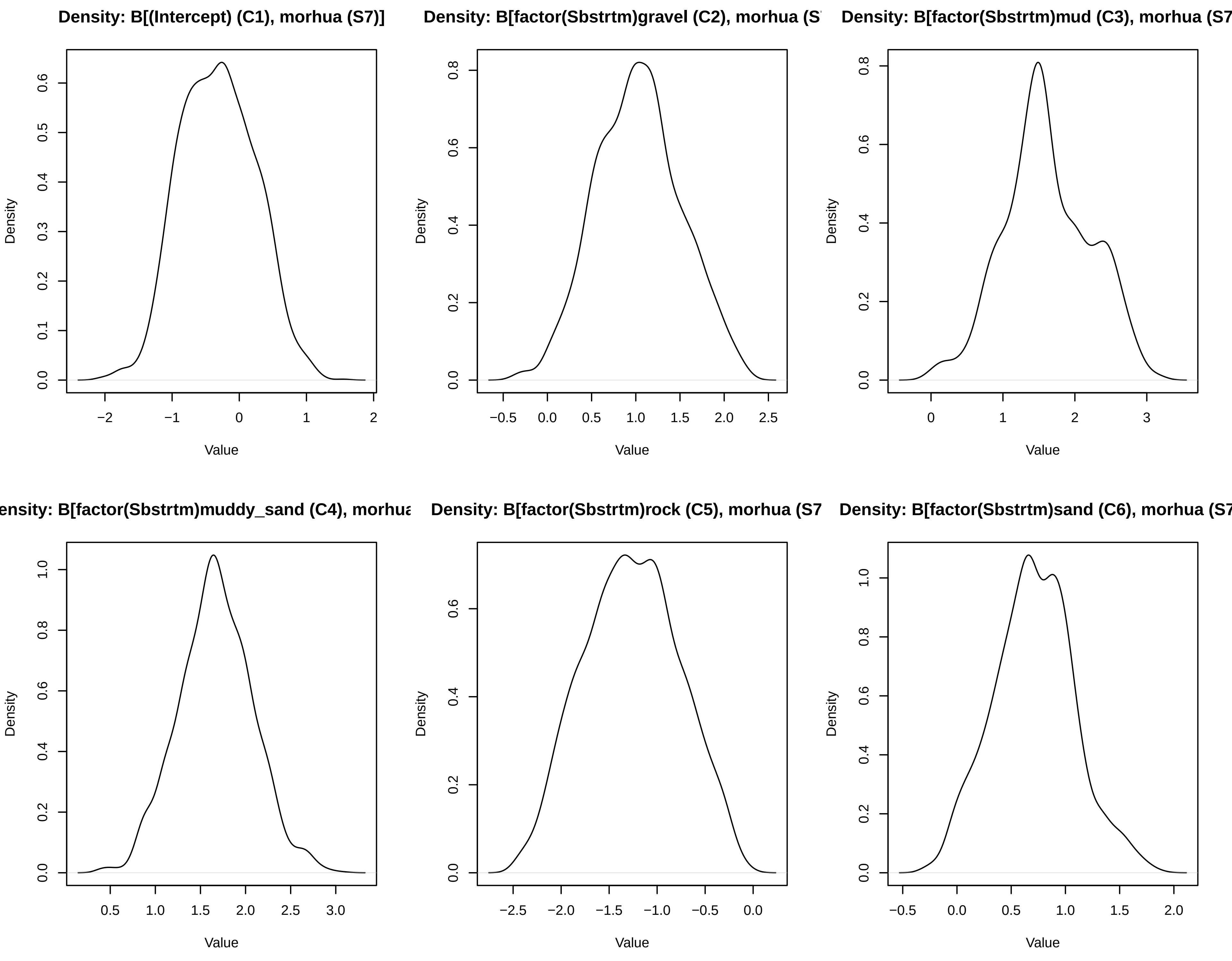

Density: B[(Intercept) (C1), morhua (S7)]
Density: B[factor(Sbstrtm)gravel (C2), morhua (S
Density: B[factor(Sbstrtm)mud (C3), morhua (S7
ensity: B[factor(Sbstrtm)muddy_sand (C4), morhua
Density: B[factor(Sbstrtm)rock (C5), morhua (S7
Density: B[factor(Sbstrtm)sand (C6), morhua (S7
Density
Value

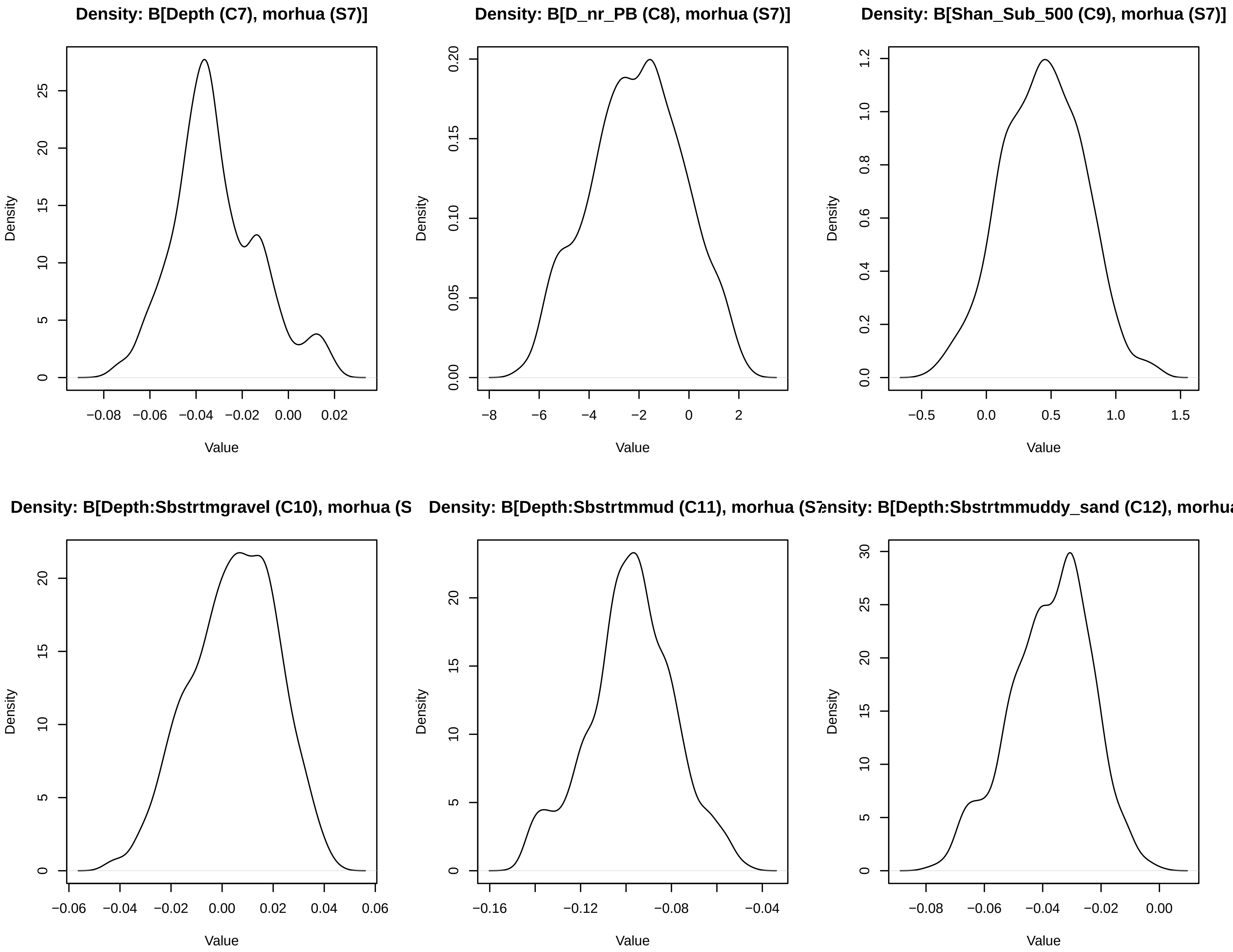
Density: B[Depth (C7), morhua (S7)]
Density: B[D_nr_PB (C8), morhua (S7)]
Density: B[Shan_Sub_500 (C9), morhua (S7)]
Density: B[Depth:Sbstrtmgravel (C10), morhua (S
Density: B[Depth:Sbstrtmmud (C11), morhua (S
ensity: B[Depth:Sbstrtmmuddy_sand (C12), morhua
Density
Value
0
5
10
15
20
25
30
−0.08
−0.06
−0.04
−0.02
0.00
0.02
0.00
0.05
0.10
0.15
0.20
−8
−6
−4
−2
2
0.0
0.2
0.4
0.6
0.8
1.0
1.2
−0.5
0.5
1.5
−0.06
0.04
0.06
−0.16
−0.12
−0.08
−0.04

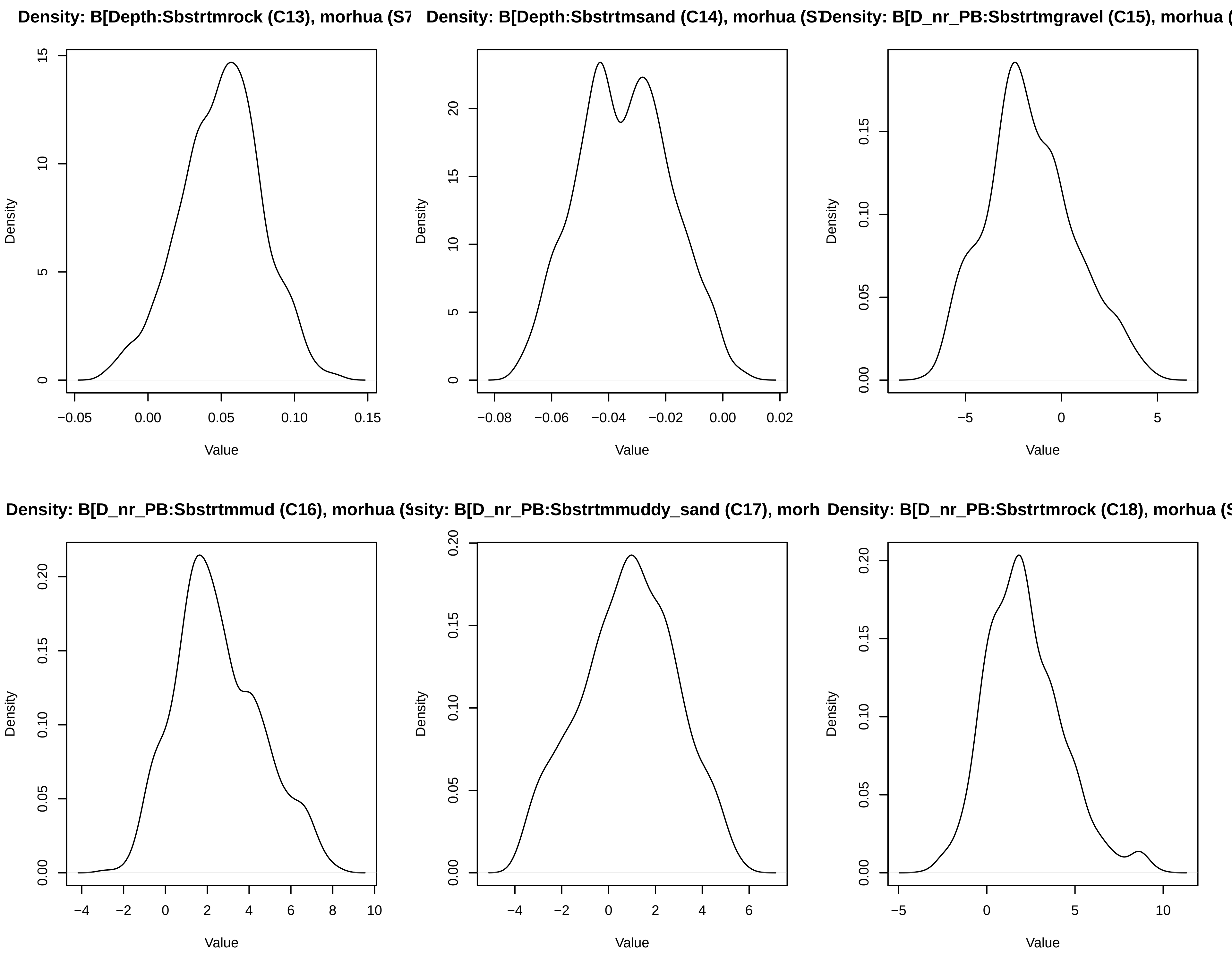
Density: B[Depth:Sbstrtmrock (C13), morhua (S7
Density: B[Depth:Sbstrtmsand (C14), morhua (S7
Density: B[D_nr_PB:Sbstrtmgravel (C15), morhua (
Density: B[D_nr_PB:Sbstrtmmud (C16), morhua (S
sity: B[D_nr_PB:Sbstrtmmuddy_sand (C17), morhu
Density: B[D_nr_PB:Sbstrtmrock (C18), morhua (S
Density
Value

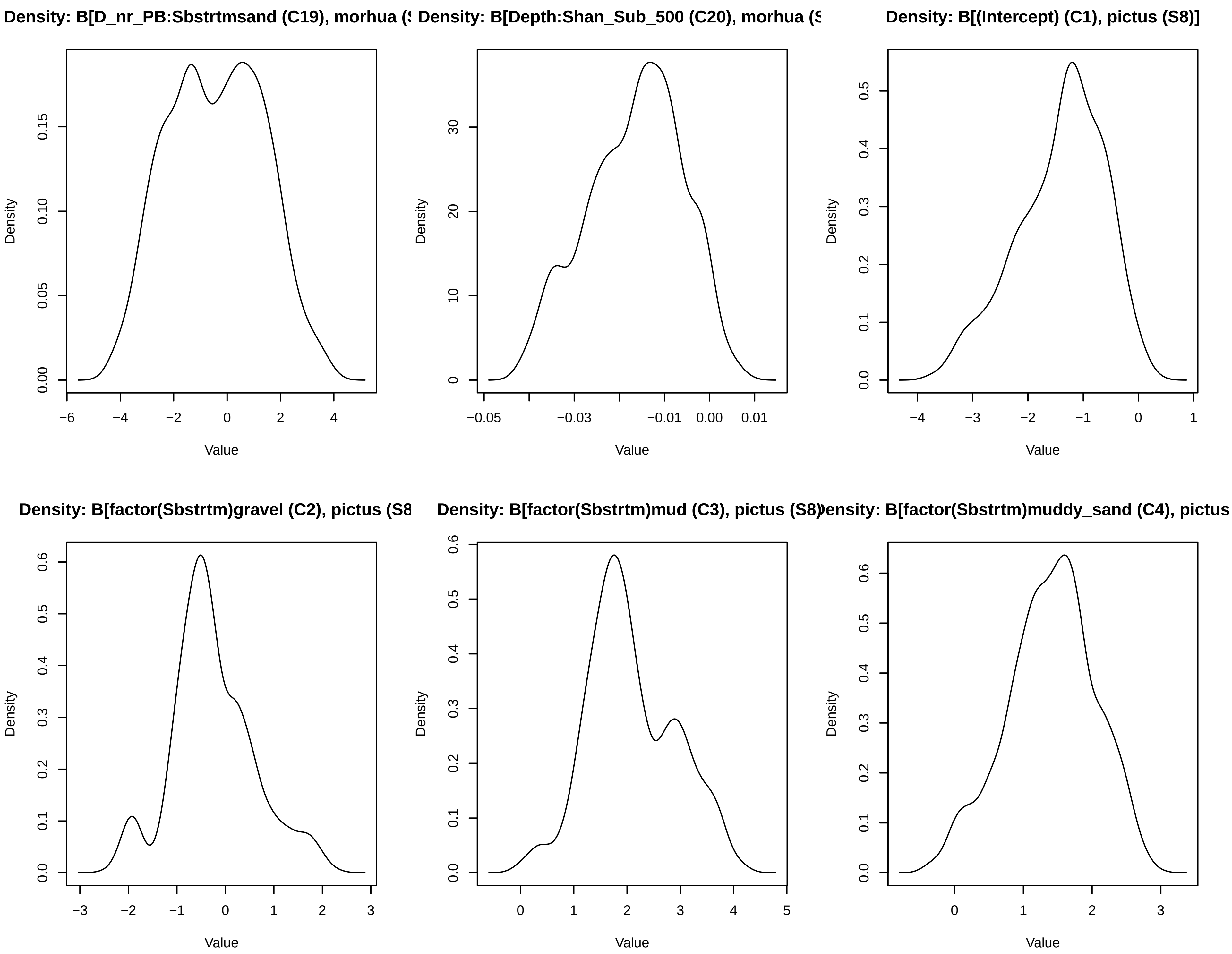
Density: B[D_nr_PB:Sbstrtmsand (C19), morhua (S
Density
Value
Density: B[Depth:Shan_Sub_500 (C20), morhua (S
Density
Value
Density: B[(Intercept) (C1), pictus (S8)]
Density
Value
Density: B[factor(Sbstrtm)gravel (C2), pictus (S8
Density
Value
Density: B[factor(Sbstrtm)mud (C3), pictus (S8)
Density
Value
ensity: B[factor(Sbstrtm)muddy_sand (C4), pictus
Density
Value

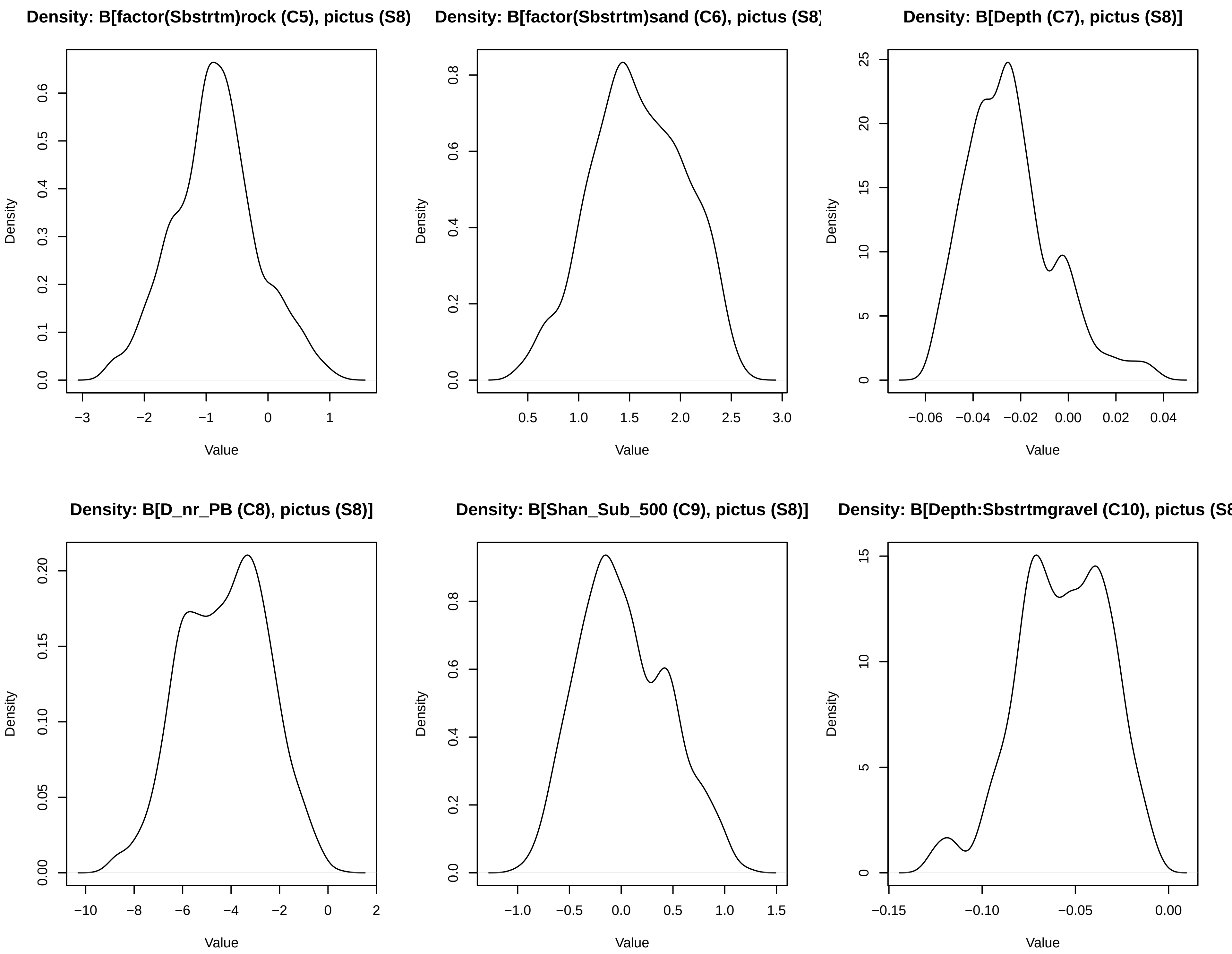

Density: B[factor(Sbstrtm)rock (C5), pictus (S8)]
Density: B[factor(Sbstrtm)sand (C6), pictus (S8]
Density: B[Depth (C7), pictus (S8)]
Density: B[D_nr_PB (C8), pictus (S8)]
Density: B[Shan_Sub_500 (C9), pictus (S8)]
Density: B[Depth:Sbstrtmgravel (C10), pictus (S8
Density
Value

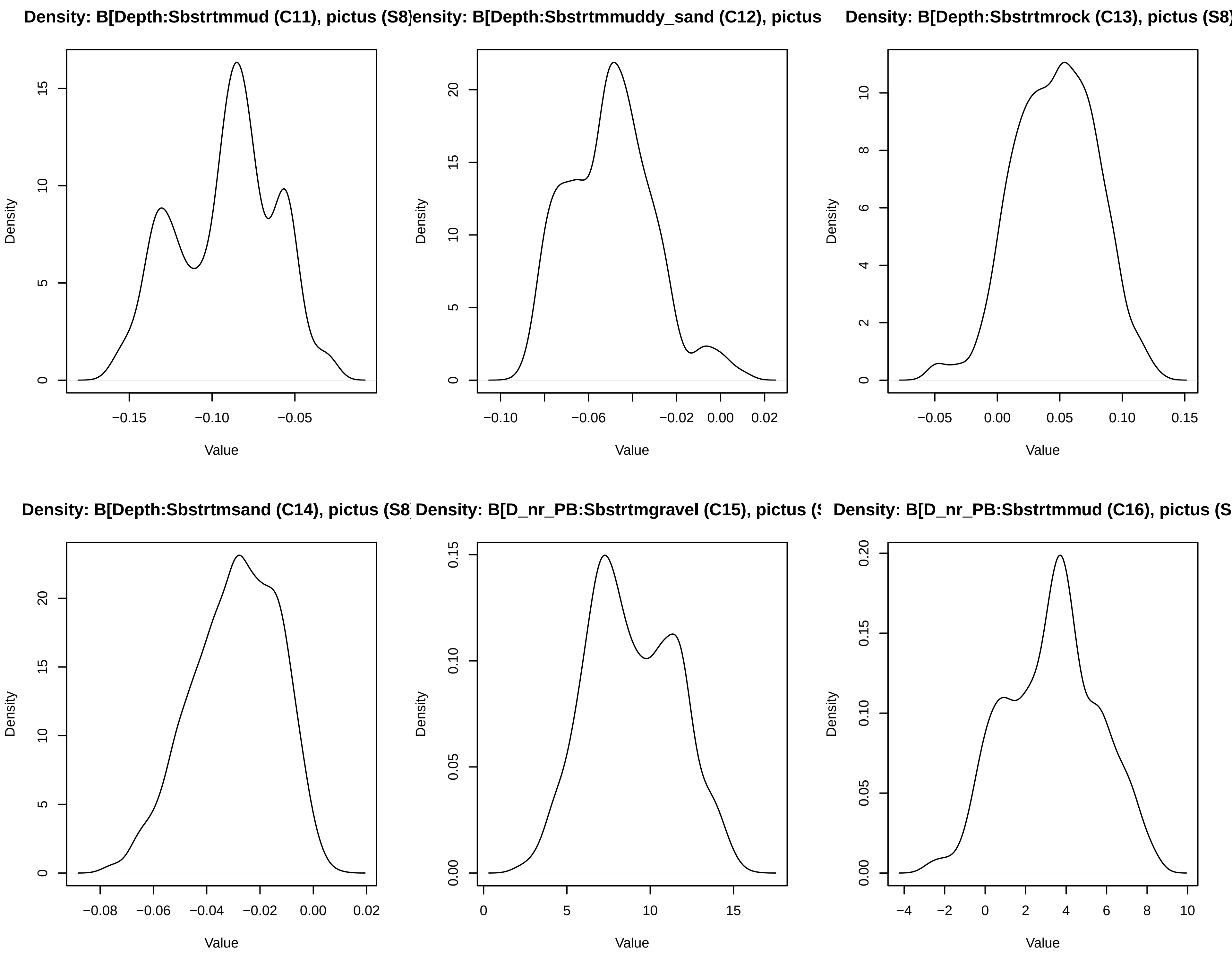
Density: B[Depth:Sbstrtmmud (C11), pictus (S8)
Density
Value
Density: B[Depth:Sbstrtmmuddy_sand (C12), pictus
Density
Value
Density: B[Depth:Sbstrtmrock (C13), pictus (S8)
Density
Value
Density: B[Depth:Sbstrtmsand (C14), pictus (S8
Density
Value
Density: B[D_nr_PB:Sbstrtmgravel (C15), pictus (S
Density
Value
Density: B[D_nr_PB:Sbstrtmmud (C16), pictus (S
Density
Value

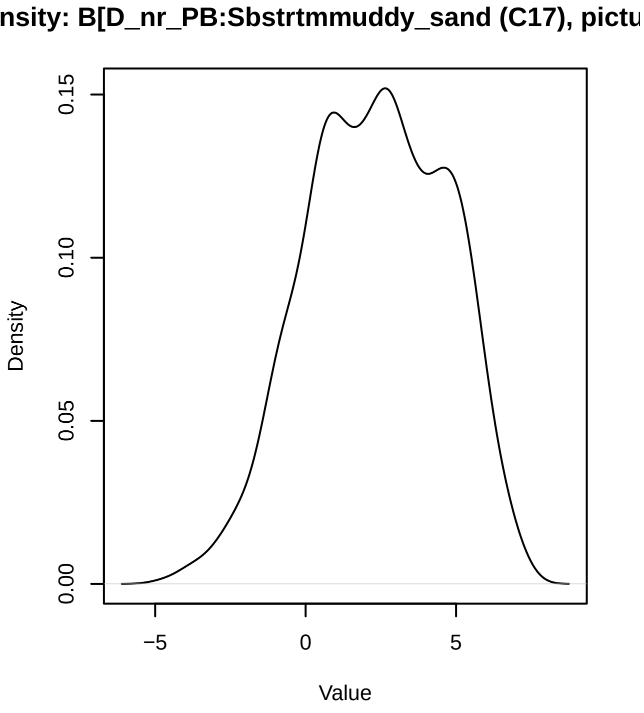
nsity: B[D_nr_PB:Sbstrtmmuddy_sand (C17), pictu
Density
Value
0.00
0.05
0.10
0.15
−5
0
5

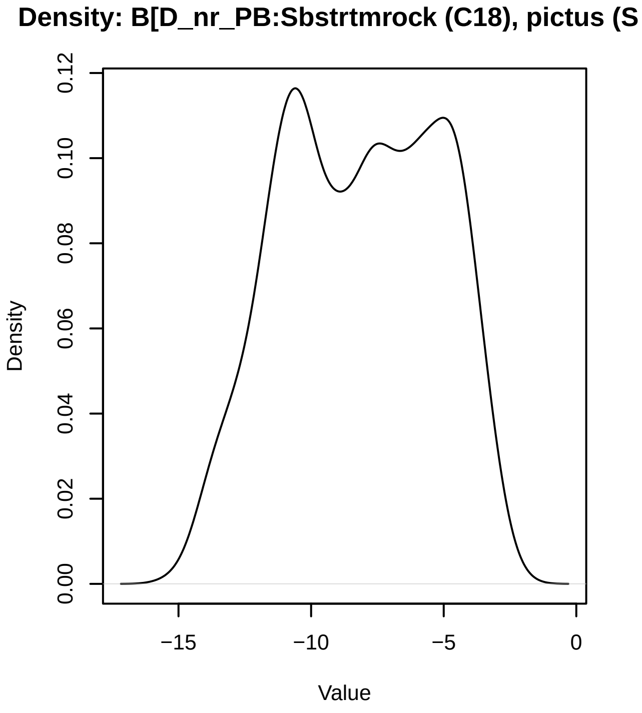
Density: B[D_nr_PB:Sbstrtmrock (C18), pictus (S
Density
Value
0.00
0.02
0.04
0.06
0.08
0.10
0.12
−15
−10
−5
0

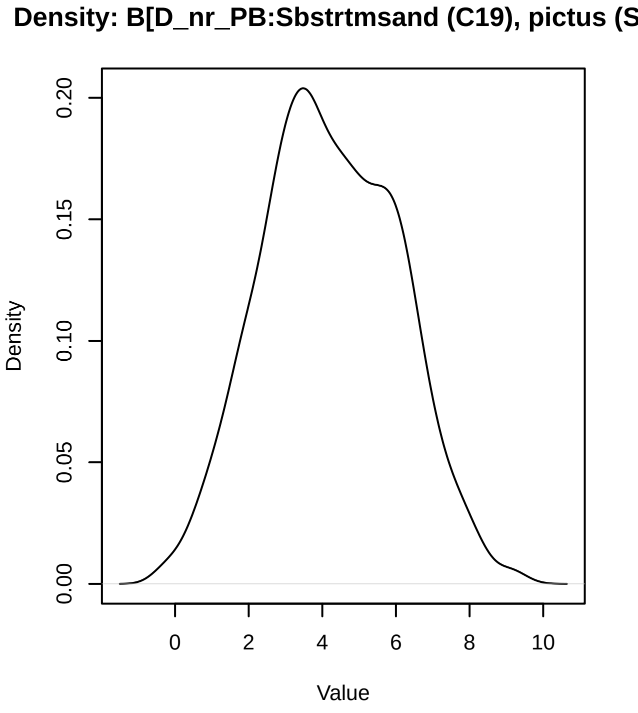
Density: B[D_nr_PB:Sbstrtmsand (C19), pictus (S
Density
Value
0.00
0.05
0.10
0.15
0.20
0
2
4
6
8
10

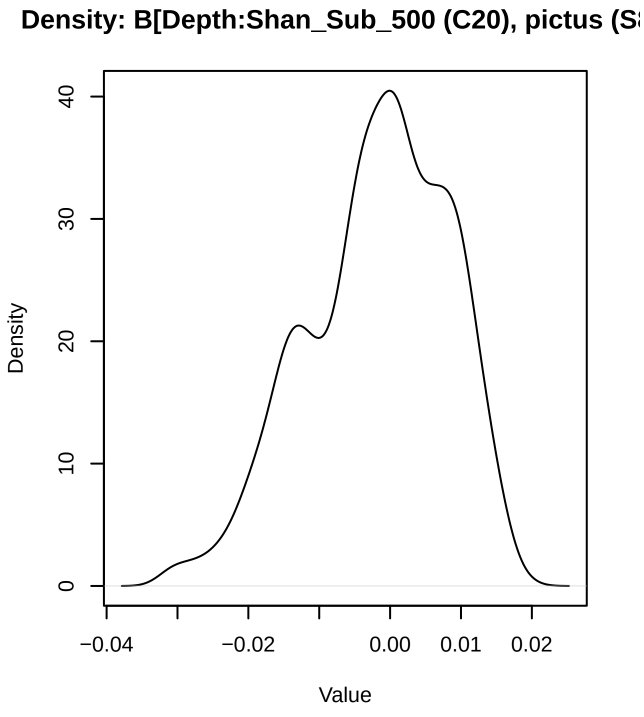
Density: B[Depth:Shan_Sub_500 (C20), pictus (S
Density
Value
0
10
20
30
40
−0.04
−0.02
0.00
0.01
0.02

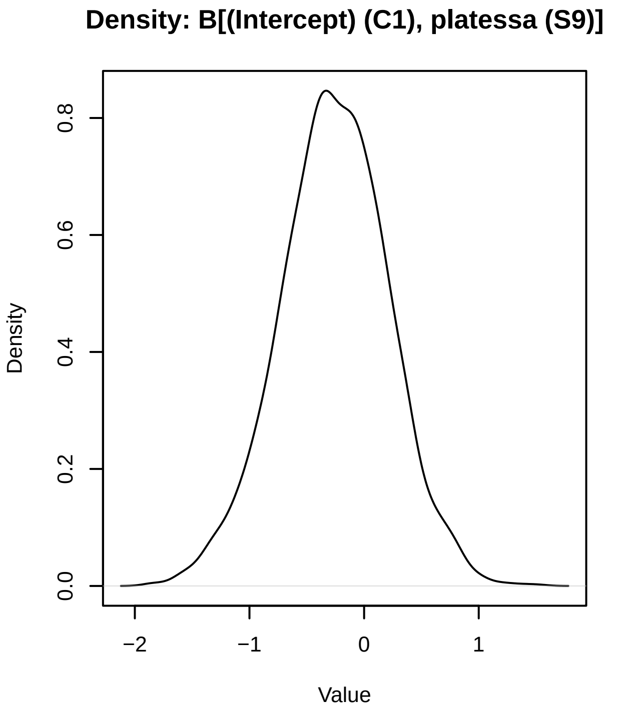
Density: B[(Intercept) (C1), platessa (S9)]
Density
Value
0.0
0.2
0.4
0.6
0.8
−2
−1
0
1

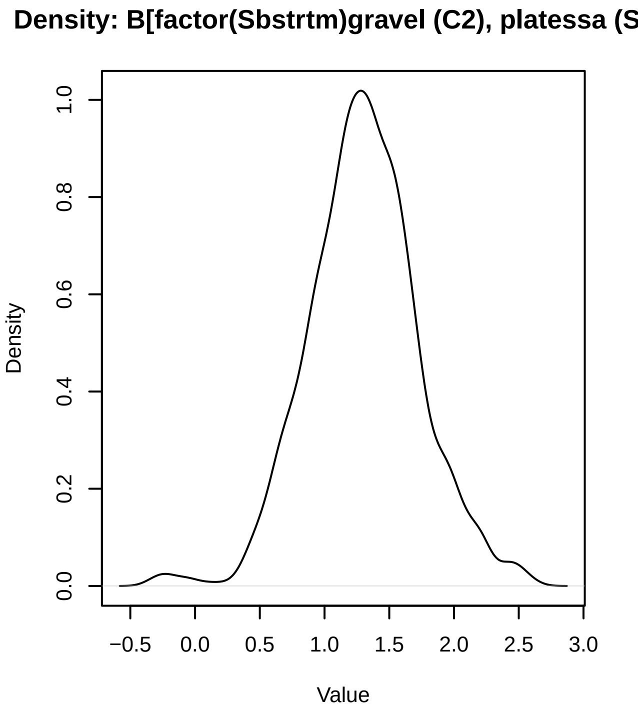
Density: B[factor(Sbstrtm)gravel (C2), platessa (S
Density
Value
0.0
0.2
0.4
0.6
0.8
1.0
−0.5
0.0
0.5
1.0
1.5
2.0
2.5
3.0

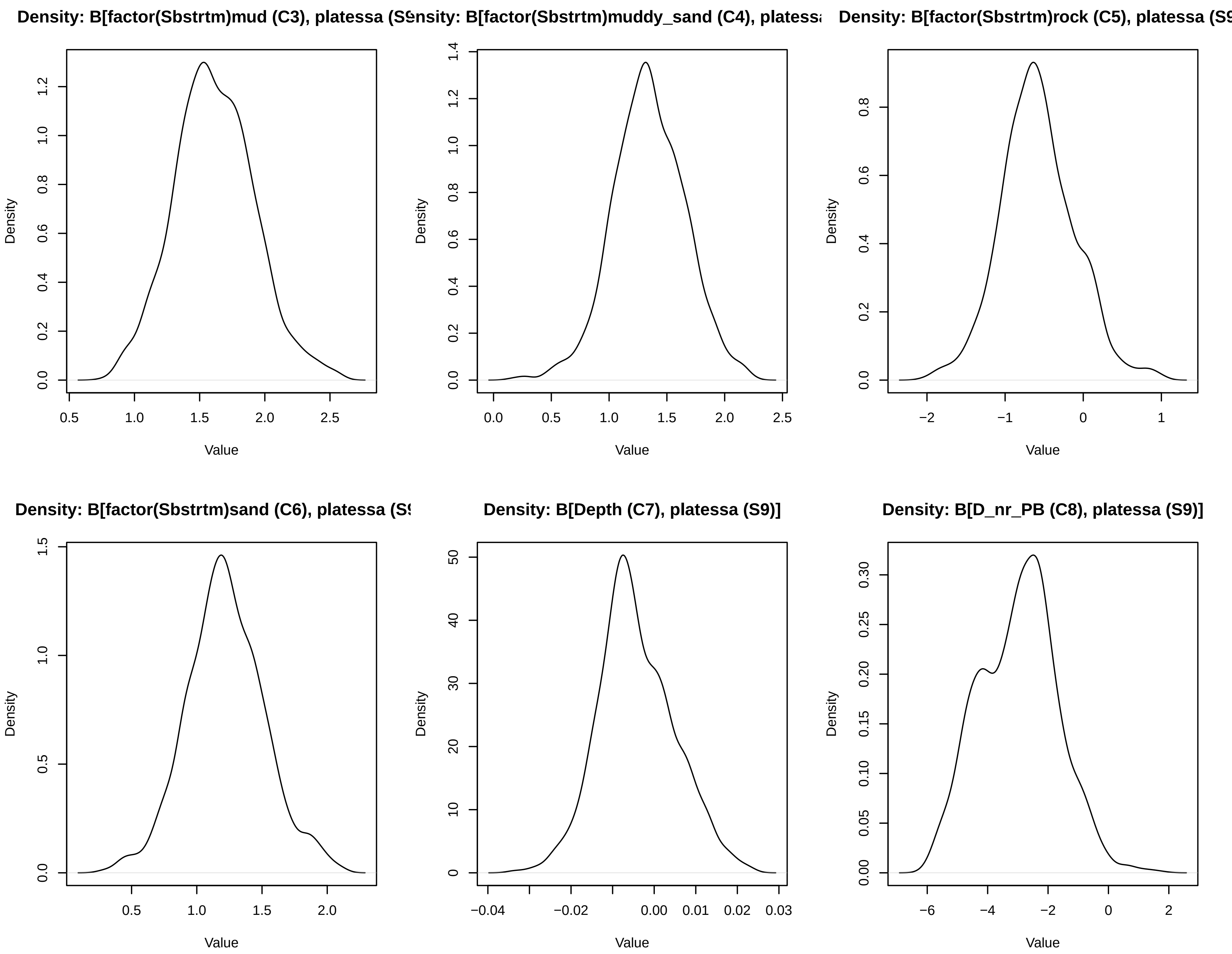
Density: B[factor(Sbstrtm)mud (C3), platessa (S9
Density: B[factor(Sbstrtm)muddy_sand (C4), platessa
Density: B[factor(Sbstrtm)rock (C5), platessa (S9
Density: B[factor(Sbstrtm)sand (C6), platessa (S9
Density: B[Depth (C7), platessa (S9)]
Density: B[D_nr_PB (C8), platessa (S9)]
Density
Value

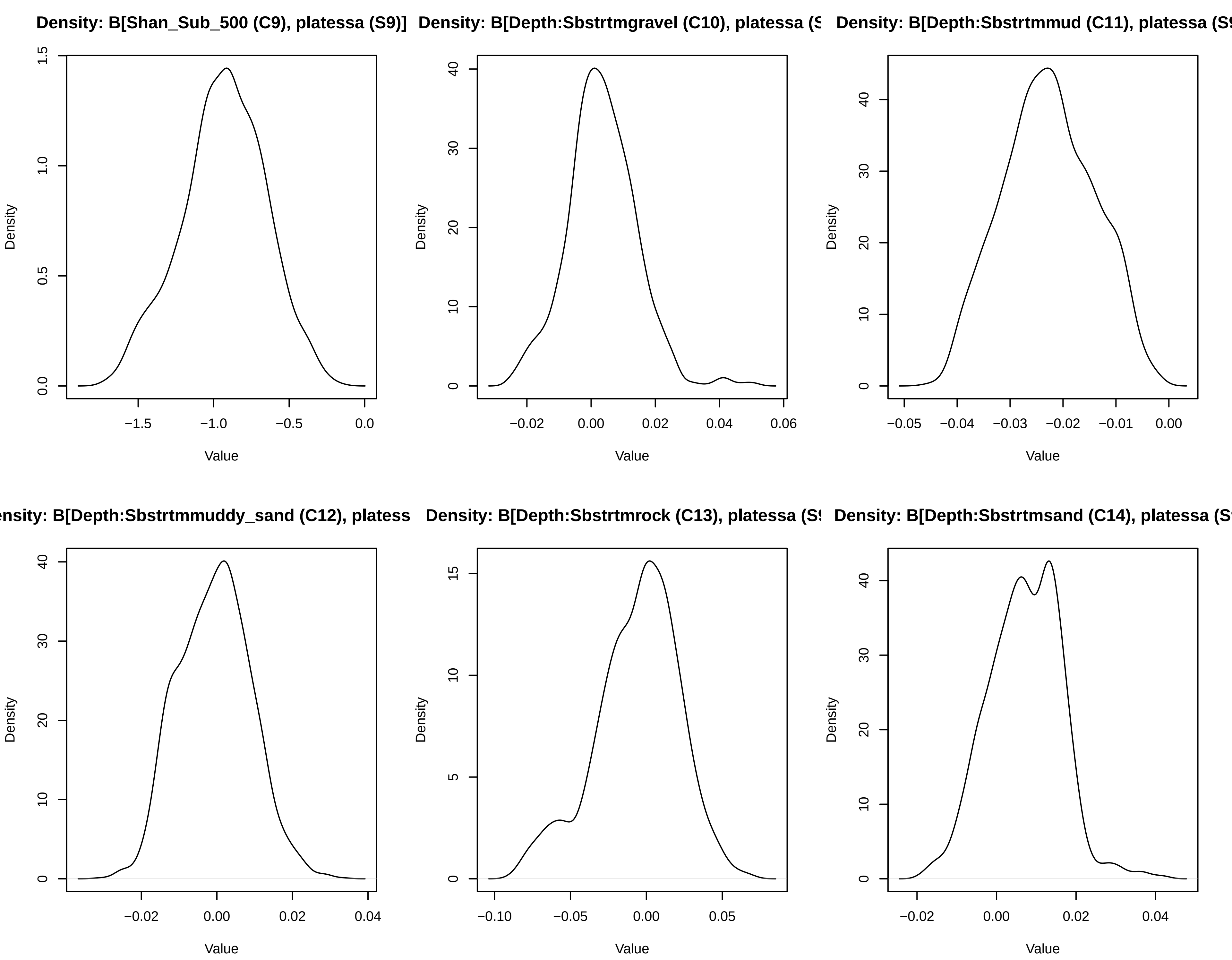
Density: B[Shan_Sub_500 (C9), platessa (S9)]
Density: B[Depth:Sbstrtmgravel (C10), platessa (S
Density: B[Depth:Sbstrtmmud (C11), platessa (S
ensity: B[Depth:Sbstrtmmuddy_sand (C12), platess
Density: B[Depth:Sbstrtmrock (C13), platessa (S
Density: B[Depth:Sbstrtmsand (C14), platessa (S
Density
Value

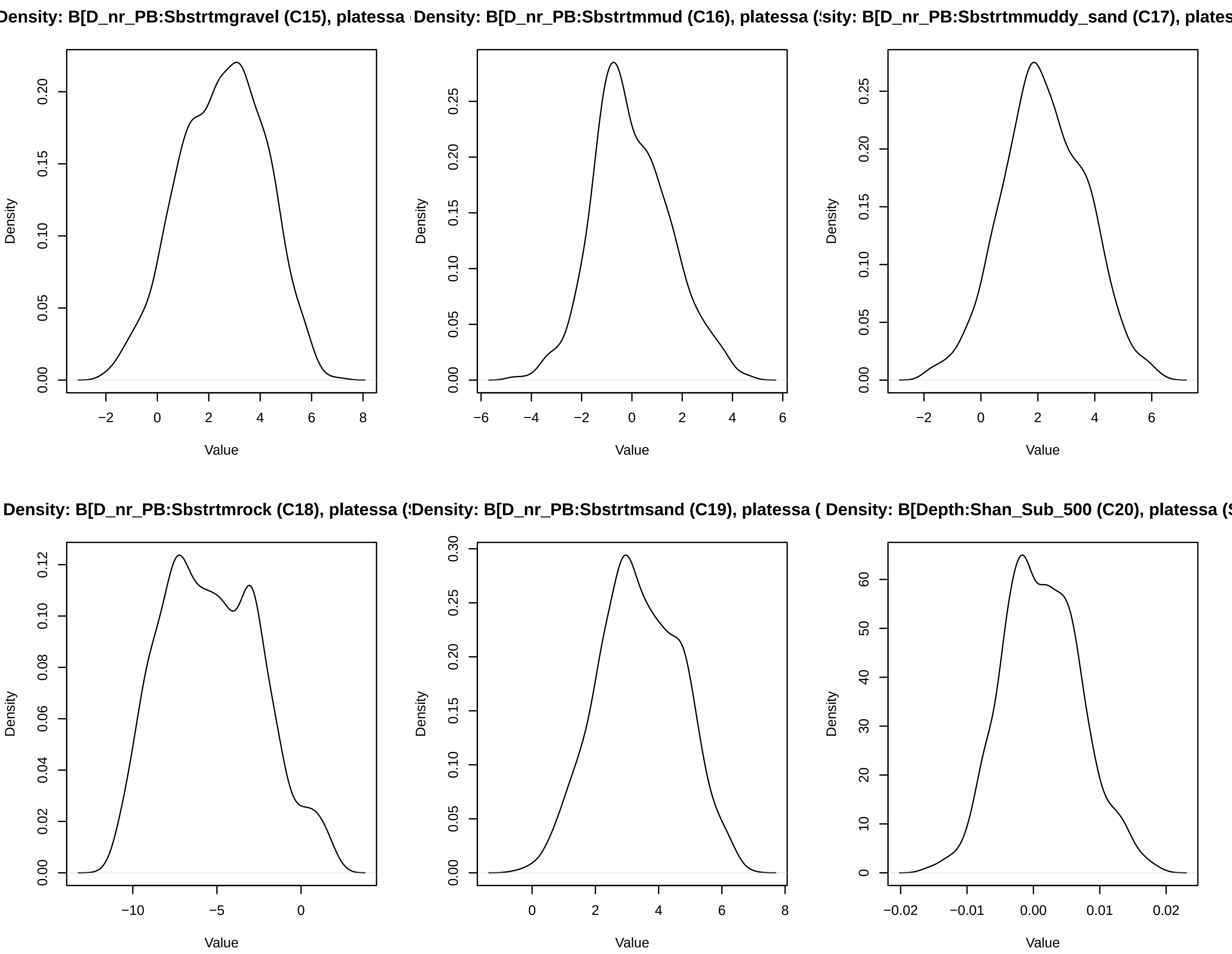

Density: B[D_nr_PB:Sbstrtmgravel (C15), platessa (
Density: B[D_nr_PB:Sbstrtmmud (C16), platessa (
sity: B[D_nr_PB:Sbstrtmmuddy_sand (C17), plates
Density: B[D_nr_PB:Sbstrtmrock (C18), platessa (
Density: B[D_nr_PB:Sbstrtmsand (C19), platessa (
Density: B[Depth:Shan_Sub_500 (C20), platessa (
Density
Value

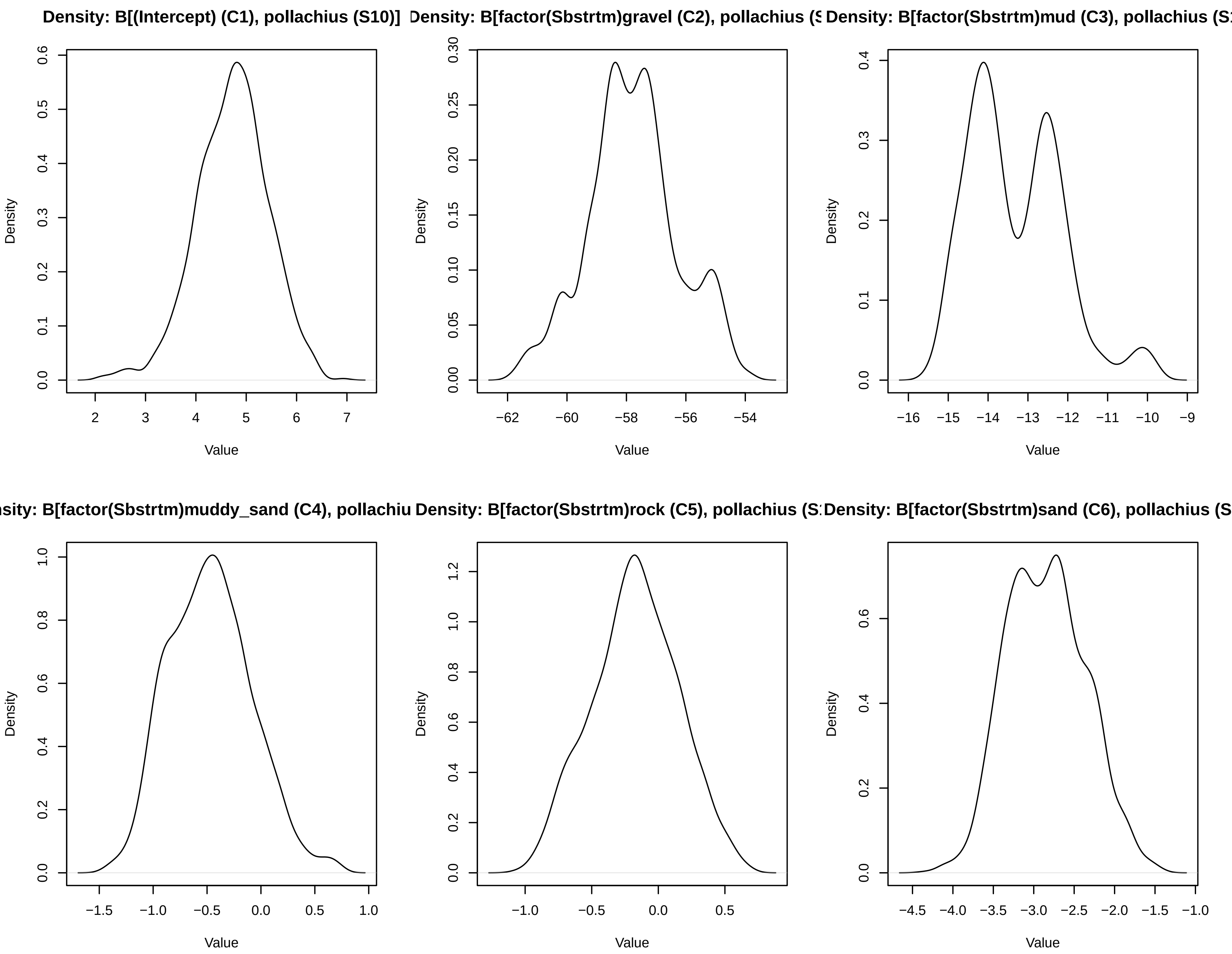

Density: B[(Intercept) (C1), pollachius (S10)]
Density: B[factor(Sbstrtm)gravel (C2), pollachius (S
Density: B[factor(Sbstrtm)mud (C3), pollachius (S
sity: B[factor(Sbstrtm)muddy_sand (C4), pollachiu
Density: B[factor(Sbstrtm)rock (C5), pollachius (S
Density: B[factor(Sbstrtm)sand (C6), pollachius (S
Density
Value

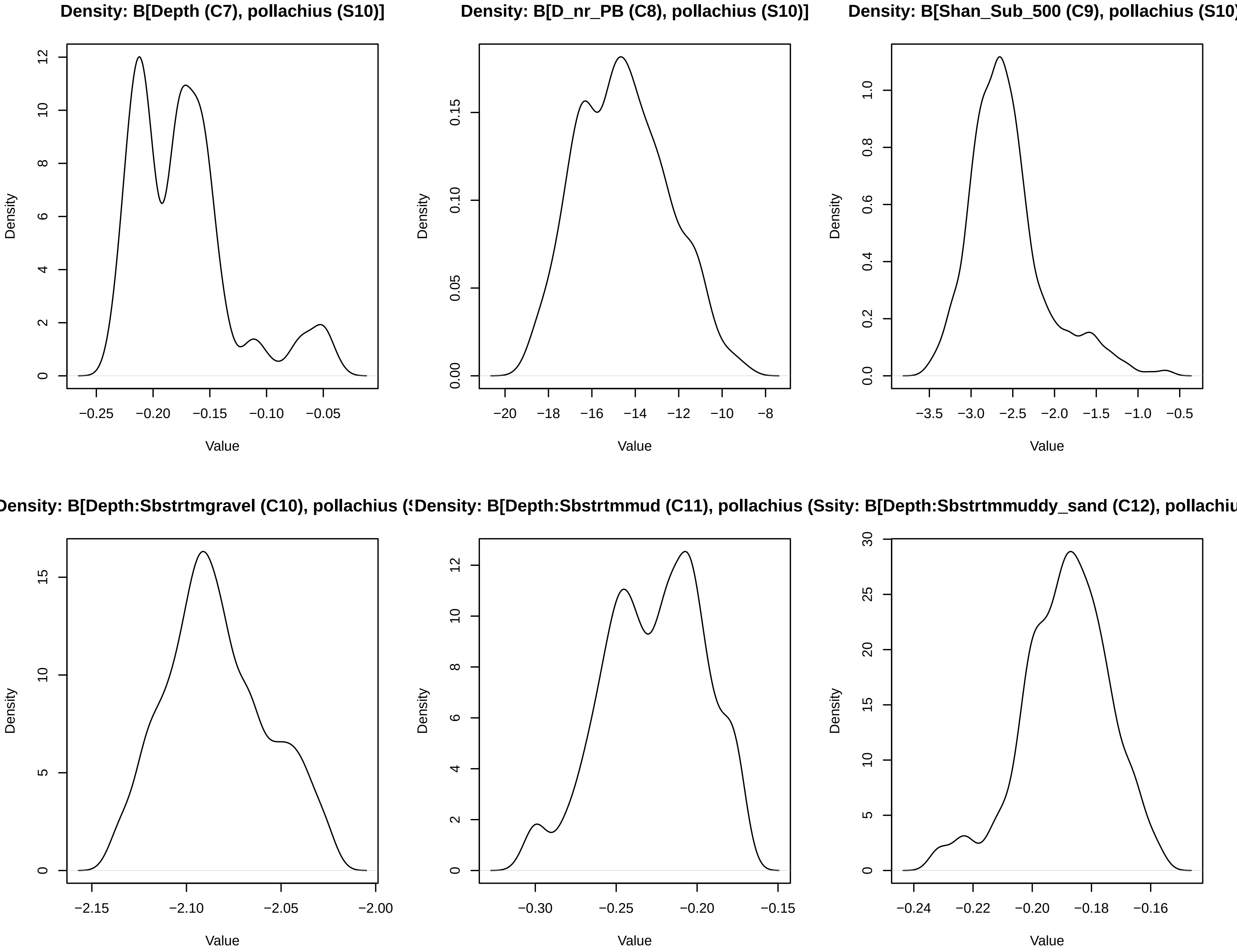
Density: B[Depth (C7), pollachius (S10)]
Density: B[D_nr_PB (C8), pollachius (S10)]
Density: B[Shan_Sub_500 (C9), pollachius (S10
Density: B[Depth:Sbstrtmgravel (C10), pollachius (S
Density: B[Depth:Sbstrtmmud (C11), pollachius (S
sity: B[Depth:Sbstrtmmuddy_sand (C12), pollachiu
Density
Value
0
2
4
6
8
10
12
−0.25
−0.20
−0.15
−0.10
−0.05
0.00
0.05
0.10
0.15
−20
−18
−16
−14
−12
−10
−8
0.0
0.2
0.4
0.6
0.8
1.0
−3.5
−3.0
−2.5
−2.0
−1.5
−1.0
−0.5
5
15
−2.15
−2.10
−2.05
−2.00
−0.30
−0.15
20
25
30
−0.24
−0.22
−0.20
−0.18
−0.16

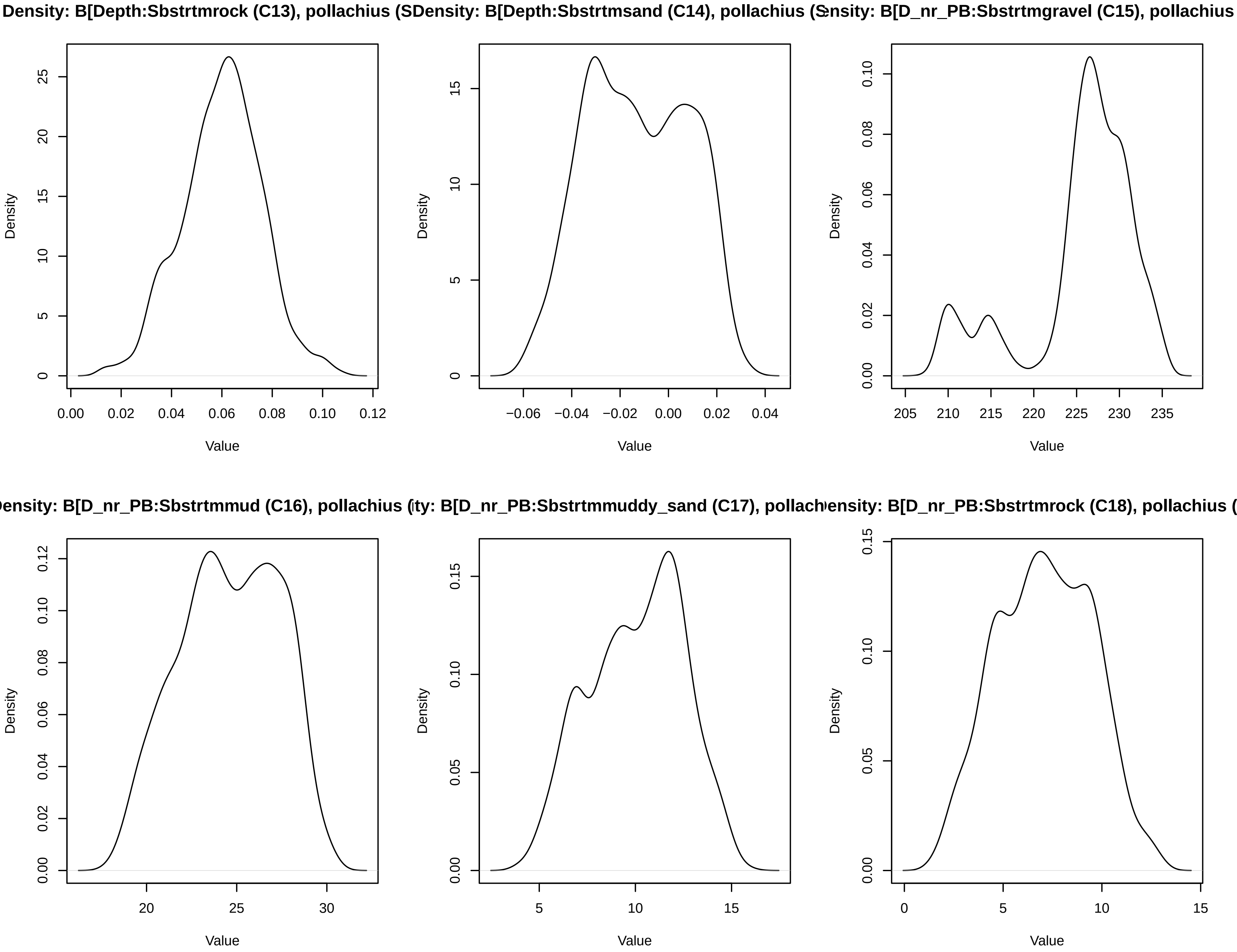
Density: B[Depth:Sbstrtmrock (C13), pollachius (S
Density: B[Depth:Sbstrtmsand (C14), pollachius (S
nsity: B[D_nr_PB:Sbstrtmgravel (C15), pollachius
ensity: B[D_nr_PB:Sbstrtmmud (C16), pollachius (
ty: B[D_nr_PB:Sbstrtmmuddy_sand (C17), pollach
ensity: B[D_nr_PB:Sbstrtmrock (C18), pollachius (
Density
Value

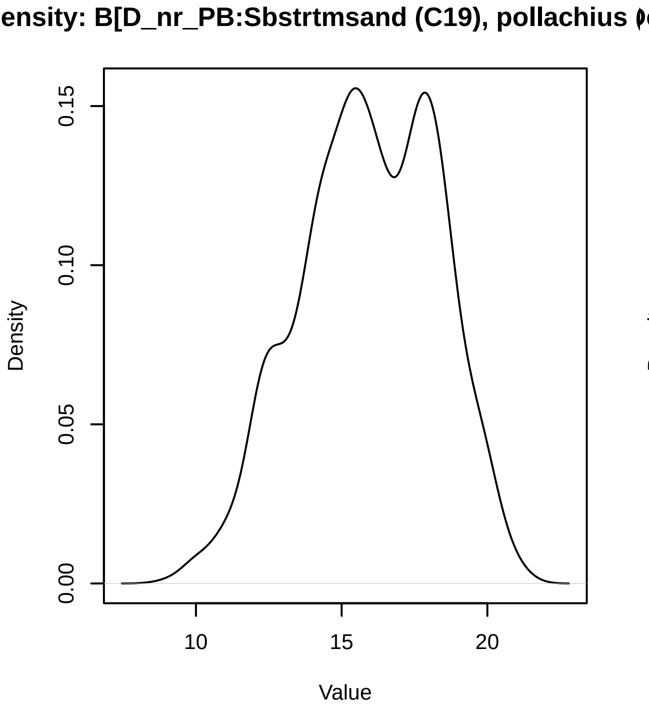
ensity: B[D_nr_PB:Sbstrtmsand (C19), pollachius
Density
0.00
0.05
0.10
0.15
10
15
20
Value

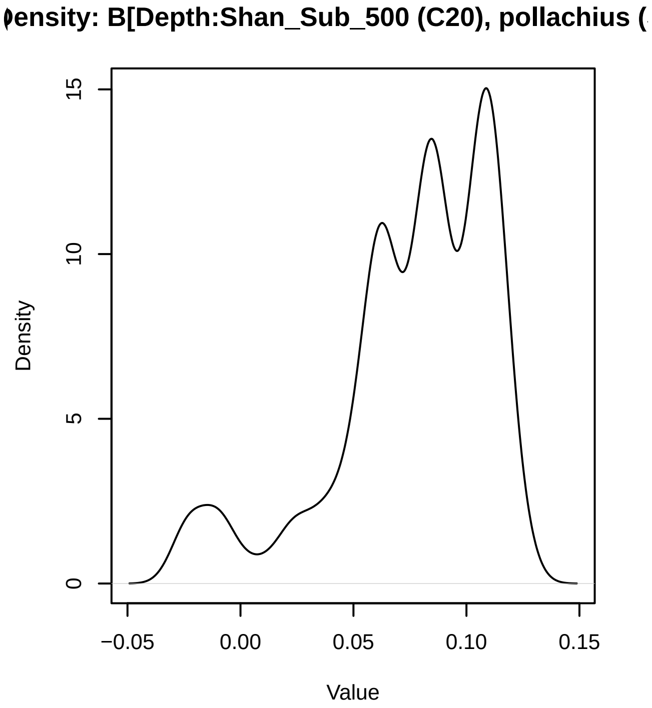
ensity: B[Depth:Shan_Sub_500 (C20), pollachius (
Density
0
5
10
15
−0.05
0.00
0.05
0.10
0.15
Value
